# Pan-Genomic Study of *Mycobacterium tuberculosis* Reflecting the Primary/Secondary Genes, Generality/Individuality, and the Interconversion Through Copy Number Variations

**DOI:** 10.3389/fmicb.2018.01886

**Published:** 2018-08-17

**Authors:** Tingting Yang, Jun Zhong, Ju Zhang, Cuidan Li, Xia Yu, Jingfa Xiao, Xinmiao Jia, Nan Ding, Guannan Ma, Guirong Wang, Liya Yue, Qian Liang, Yongjie Sheng, Yanhong Sun, Hairong Huang, Fei Chen

**Affiliations:** ^1^CAS Key Laboratory of Genome Sciences and Information, Beijing Institute of Genomics, Chinese Academy of Sciences, Beijing, China; ^2^University of Chinese Academy of Sciences, Beijing, China; ^3^National Clinical Laboratory on Tuberculosis, Beijing Key Laboratory on Drug-Resistant Tuberculosis Research, Beijing Chest Hospital, Capital Medical University, Beijing Tuberculosis and Thoracic Tumor Institute, Beijing, China; ^4^BIG Data Center, Beijing Institute of Genomics, Chinese Academy of Sciences, Beijing, China; ^5^Peking Union Medical College Hospital, Chinese Academy of Medical Sciences and Peking Union Medical College, Beijing, China; ^6^Key Laboratory for Molecular Enzymology and Engineering of Ministry of Education, Jilin University, Changchun, China; ^7^Collaborative Innovation Center for Genetics and Development, Beijing, China

**Keywords:** *Mycobacterium tuberculosis* (Mtb), pan-genome, core gene, selection pressure, adaptive evolution, infectious disease, copy number variation (CNV), host-adaptation

## Abstract

Tuberculosis (TB) has surpassed HIV as the leading infectious disease killer worldwide since 2014. The main pathogen, *Mycobacterium tuberculosis* (Mtb), contains ~4,000 genes that account for ~90% of the genome. However, it is still unclear which of these genes are primary/secondary, which are responsible for generality/individuality, and which interconvert during evolution. Here we utilized a pan-genomic analysis of 36 Mtb genomes to address these questions. We identified 3,679 Mtb core (i.e., primary) genes, determining their phenotypic generality (e.g., virulence, slow growth, dormancy). We also observed 1,122 dispensable and 964 strain-specific secondary genes, reflecting partially shared and lineage-/strain-specific individualities. Among which, five L2 lineage-specific genes might be related to the increased virulence of the L2 lineage. Notably, we discovered 28 Mtb “Super Core Genes” (SCGs: more than a copy in at least 90% strains), which might be of increased importance, and reflected the “super phenotype generality.” Most SCGs encode PE/PPE, virulence factors, antigens, and transposases, and have been verified as playing crucial roles in Mtb pathogenicity. Further investigation of the 28 SCGs demonstrated the interconversion among SCGs, single-copy core, dispensable, and strain-specific genes through copy number variations (CNVs) during evolution; different mutations on different copies highlight the delicate adaptive-evolution regulation amongst Mtb lineages. This reflects that the importance of genes varied through CNVs, which might be driven by selective pressure from environment/host-adaptation. In addition, compared with *Mycobacterium bovis* (Mbo), Mtb possesses 48 specific single core genes that partially reflect the differences between Mtb and Mbo individuality.

## Introduction

Tuberculosis (TB) is a major global health threat, and surpassed HIV as the number-one infectious disease killer since 2014. According to the World Health Organization's (WHO's) “2017 Global Tuberculosis Report,” in 2016 there were ~10.4 million new TB cases and ~1.7 million people died from TB worldwide (World Health Organization, 2017). Moreover, it is also estimated that about one-third of the world's population (~2 billion people) has latent TB, and that 5-10% of these people will develop active TB disease at some time during their life (Hauck et al., 2009; Lv et al., 2017). Human TB is mainly caused by *Mycobacterium tuberculosis* (Mtb), which includes five lineages (L1–L4 and L7): L1 (The Philippines and Indian Ocean), L2 (East Asia), L3 (India and East Africa), L4 (Europe and Americas), and L7 (Ethiopia) (Gonzalo-Asensio et al., 2014; Zhu et al., 2016). Approximately 4,000 genes account for ~90% of the Mtb genome (~4.4 Mb). However, it is still unclear which of these genes are primary and which are secondary, which are responsible for generality and individuality, and which interconvert during evolution.

Pan-genomic analyses provide a powerful tool for determining primary and secondary genes, assessing the generality and individuality of Mtb strains, and exploring gene interconversion. The pan-genome is defined as the entire genomic repertoire of a given species, including “core genes” shared by all strains and “accessory genes” that are not present in all strains (Vernikos et al., 2015). The accessory genes consist of both “dispensable genes” (i.e., genes present in two or more strains) and “strain-specific genes” (i.e., genes specific to a single strain). The core genes are considered primary genes; they determine the generality of all strains of a species and contain the majority of genes essential for survival. They are closely related to the most basic and important biological characteristics of the species (i.e., generality) (Vernikos et al., 2015). For example, Choo et al. (2014) found that most of the core genes of 40 *Mycobacterium abscessus* were related to survival, demonstrating the generality of these strains. Dispensable genes and strain-specific genes are considered secondary genes, determining the partially shared and strain-specific features of a species (i.e., characteristics that distinguish strains from one another) and are not essential for survival (Vernikos et al., 2015). These partially shared or strain-specific genes are also important for some lineage-strains/strains to adapt to certain circumstances. In a pan-genomic analysis of 21 commensal and nine nosocomial *Staphylococcus epidermidis*, Conlan et al. (2012) discovered that the formate dehydrogenase gene was found only in commensals; this demonstrates the individuality of commensal strains and distinguishes them from nosocomial strains.

Since core genes are essential for survival and determine the generality of all strains of a species, multi-copy core genes are particularly important and reflect “super phenotype generality.” Related research has revealed that the more copies of a gene with important function, the greater the contribution that gene made to a species survival (Yu et al., 2015). Multiple copies serve to assure a high expression level and further reduces the risk of protein folding errors caused by mistranslation of mRNA (which might be toxic to cells) (Yang et al., 2010; Kiwuwa et al., 2013). In some cases, the increased gene copies with various mutations could serve as an evolutionary force for new gene functions (e.g., bacterial drug resistance, virulence) (Bennett, 2004; Sandegren and Andersson, 2009; Galagan, 2014).

To date, we have found no examples of pan-genomic analysis of Mtb. Determining the set of Mtb primary genes will help to uncover the genetic basis for Mtb phenotype generality (e.g., virulence, slow growth, latency). Meanwhile, determining the set of Mtb accessory genes (both dispensable and strain-specific secondary genes) will show the genetic basis of partially shared and strain-specific phenotype features. It is also important to be able to distinguish between different Mtb lineages. For example, the analysis of dispensable genes may reveal the genetic reason for the increased virulence of the Beijing strains (L2 lineage) (Parwati et al., 2010). Most importantly, pan-genomic analysis provides a tool for investigating multi-copy core genes, which are of increased importance and reflect the key genetic factors of “super generality” of Mtb phenotypes.

In this study, we performed a pan-genomic analysis of 36 complete Mtb genomes (three L1, fifteen L2, one L3, and seventeen L4 strains) to investigate the primary and secondary genes, the generality and individuality of Mtb strains, and the interconversion of genes. As controls, we performed a pan-genomic analysis of 13 *Mycobacterium bovis* (Mbo) genomes (four *M. bovis* and nine *M. bovis* BCG strains). The results identified 3,679 Mtb core (primary) genes as well as 1,122 dispensable and 964 strain-specific genes (secondary genes).

Most notably, we discovered 28 core genes with two or more copies in more than 90% strains (defined as “Super Core Genes”; SCGs), which are likely of increased importance and reflect the key genetic factors of “super generality” of Mtb phenotypes. Most SCGs have been experimentally verified to encode for important proteins, such as PE/PPE proteins, virulence factors (VFs), antigens, or transposases. Further investigation of the 28 SCGs indicated that their copy numbers changed during evolution: SCGs, core genes, dispensable genes, and strain-specific genes can be converted between one other. This demonstrates that the importance of genes varied through the copy number variations at different stages of evolution, facilitating strains to adapt to various circumstances. Selection pressure was proven to be one of the driving forces for these interconversions.

In summary, a pan-genomic analysis of Mtb revealed the primary and secondary genes, the generality and individuality of Mtb strains, and the possibility for interconversion by CNVs. This study serves to develop an important theoretical basis for TB research, as well as provide a new research paradigm for studying organisms with a pan-genomic approach.

## Materials and methods

### Bacterial genomes and phylogeny

The 36 assembled complete genomes of human-adapted Mtb strains were obtained from NCBI, and included three L1, 15 L2, one L3, and 17 L4 strains (Supplementary Table S1). For comparison, 13 complete genomes of animal-adapted Mbo strains (4 *M. bovis* and 9 *M. bovis* BCG strains; Supplementary Table S2) downloaded from NCBI were also analyzed.

### Phylogenetic analysis

The phylogenetic analysis of the Mtb and Mbo strains was based on the 43,878 whole-genome single nucleotide polymorphisms (SNPs) detected by MUMmer 3.23 (Kurtz et al., 2004) using *Mycobacterium canettii* CIPT 140010059 (one member of smooth tubercle bacilli: STB) as the reference. The MAFFT were adopted to align the concatenated SNP sequences from the 36 Mtb strains, 13 Mbo strains, and *M. canettii* CIPT 140010059 (as an out-group) (Katoh et al., 2002); the phylogenetic relationships between these strains were analyzed using the maximum likelihood method with MEGA 6.06 (Tamura et al., 2013). Bootstraps were performed with 500 replicates.

### Genome re-annotation

All of the 36 Mtb and 13 Mbo genome sequences were re-annotated with the “Rapid Annotation using Subsystem Technology (RAST)” pipeline (Aziz et al., 2008). The protein functions were annotated with the Clusters of Orthologous Groups (COG) (Tatusov et al., 1997; Wu et al., 2011).

Several BLAST surveys against reference sequences (50% coverage and 90% identity) were performed to search for PE/PPE proteins, VFs, and antigens in the 36 Mtb strains and 13 Mbo strains. 157 PE/PPE were from the annotated proteins in the *M. tuberculosis* H37Rv (NC_000962); 255 predicted VFs were from the Virulence Factor database (VFDB) (Chen et al., 2016); 469 antigens were manually downloaded from NCBI based on the GI numbers of epitopes from the Immune Epitope Database (IEDB) (Vita et al., 2015).

### PAN-genome analyses

All proteins from the 36 Mtb and the 13 Mbo strains were clustered by MP method in the pan-genome analysis pipeline (PGAP) (Zhao et al., 2012). The characteristic curves of the Mtb/Mbo pan-genome, the core-genome, and the new genes were depicted using the Pan-Genome Profile Analyze Tool (PanGP) (Zhao et al., 2014) with DG sampling algorithms. The COG functional enrichment analyses of Mtb/Mbo core and dispensable genes were conducted by PGAP with the parameter “-function.” Similarly, the pan-genome analyses of PE/PPE proteins, VFs, and antigens were also performed using the aforementioned methods.

### Identification of multi-copy core genes and species/lineage specific single-copy core genes

The multi-copy core genes were defined as core genes containing more than one copy in at least one strain from this study. SCGs, HCGs, and MCGs were identified according to the proportion of Mtb strains containing the multi-copy core genes. The Mtb specific single-copy core genes were defined as those present in all Mtb strains with one copy and absent from all Mbo strains. The L2/L4 specific genes were identified using similar parameters. Heatmaps showing the CNVs of multi-copy core genes of the Mtb and Mbo strains were created by the “pheatmap” program in R 3.2.1 using “RColorBrewer” as the color scheme. The species/lineage specific genes were also displayed with the “pheatmap” program in R 3.2.1.

### Evolution of SCGs

To investigate the evolutionary processes of the two Mtb SCGs (*PE_PGRS33* and *fadD15*) deeply, 29 complete mycobacterial reference genomes were downloaded from NCBI. The 16S rRNA sequences of the 29 strains were used to construct a mycobacterial species tree (Lawson et al., 1993). The copy numbers and genotypes of the two Mtb SCGs in each of the MTBC/mycobacterial strains were mapped to the phylogenetic tree to illuminate the evolutionary process.

### Ka/Ks calculation

Ka/Ks values were calculated using the orthologs between each Mtb strain and *M. canettii* CIPT 140010059, as identified by the InParanoid program (O'Brien et al., 2005). For each kind of gene (i.e., all-genes, SCGs, HCGs, MCGs, single-copy core genes, or dispensable genes), Ka/Ks values were calculated with ParaAT1.0 (Zhang et al., 2012). Genes with abnormal Ka/Ks values (Ka/Ks < 0.01 or Ka/Ks > 10) were excluded when calculating the average Ka/Ks values for each type of gene.

## Results

### Open PAN-genome of Mtb strains

The 36 Mtb complete genomes (Supplementary Figure S1) were obtained from NCBI and re-annotated RAST pipeline (Aziz et al., 2008). They consist of a ~4.4 Mb genome (~65.6 GC-content) and ~4,400 predicted protein-coding sequences (CDSs), which account for ~90.4% of the genome (Supplementary Table S1).

**Figure 1:**
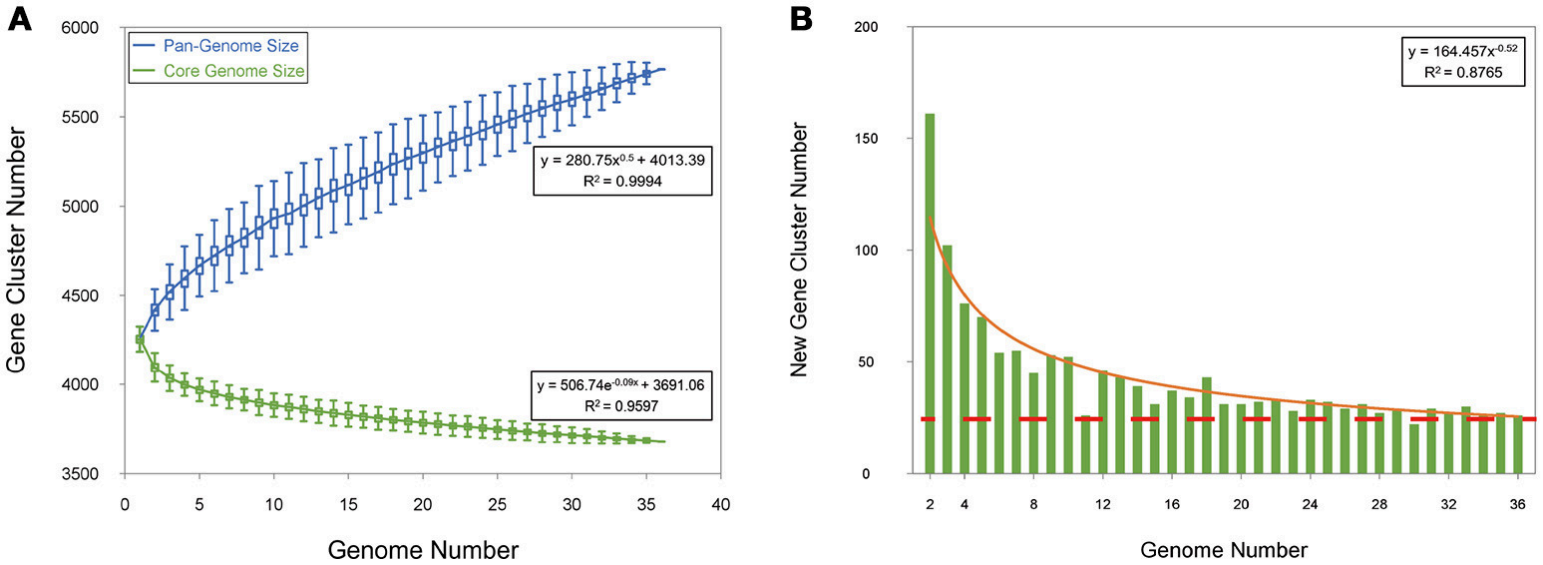
Pan-genome of Mtb. **(A)** Gene accumulation curves of the pan-genome (blue) and core-genome (green). The blue boxes denote the Mtb pan-genome size for each genome for comparison. The green boxes show the Mtb core genome size for each genome for comparison. The curve is the least squares fit of the power law for the average values. **(B)** Curve (red) for the number of new genes with an increase in the number of Mtb genomes.

**Figure 2:**
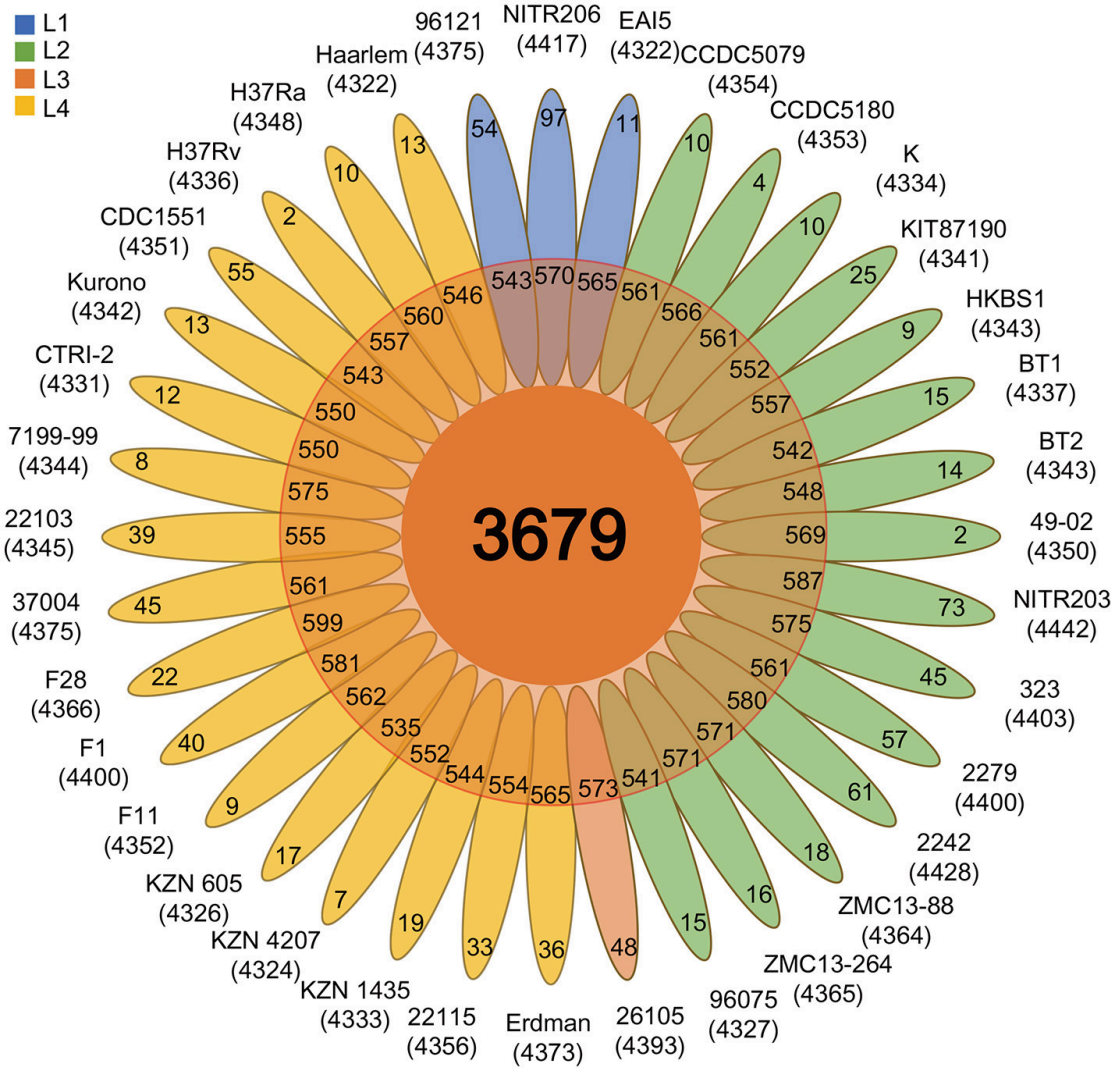
Flower plot showing the core, dispensable, and strain-specific genes of the 36 Mtb strains. The flower plot displays the core gene number (in the center), the dispensable gene number (in the annulus), and the strain-specific gene number (in the petals) for the 36 Mtb strains. The numbers under the strain name denote the total number of related genes. Different colors indicate different lineage strains: L1 strains, blue; L2 strains, green; L3 strains, salmon; L4 strains, gold.

To analyze the Mtb pan-genome, we used PGAP software (Zhao et al., 2012) to identify 5,765 orthologous genes, including 3,679 core genes (~ 86.6%), 1,122 dispensable genes, and 964 strain-specific genes (Figures 1, 2 and Supplementary Table S3). Contrary to the traditional opinion about the conservative Mtb genome (Medini et al., 2005), the Mtb pan-genome curve indicated an open pan-genome (Figure 1A). About 25 new Mtb genes were added to the pan-genome by each genome (Figure 1B); this suggests that there is some exchange of genetic material amongst Mtb strains (Medini et al., 2005; Vernikos et al., 2015).

As controls, the Mbo pan-genome curve also indicated an open pan-genome (Supplementary Figure S2). We identified 4,914 orthologous genes in the 13 Mbo strains, including 3,904 core genes (~91% of Mbo CDSs), 567 dispensable genes, and 443 strain-specific genes (Supplementary Figure S3 and Supplementary Table S4).

### Core genes are primary genes and determine the generality of Mtb strains

To uncover the genetic basis for Mtb phenotype generality, the 3,679 Mtb core genes were classified by COG analysis (Tatusov et al., 1997). The top five enriched COG categories reflected the primary function and generality of Mtb strains (category I: Lipid transport and metabolism; category C: Energy production and conversion; category K: Transcription; category Q: Secondary metabolites biosynthesis, transport and catabolism; category E: Amino acid transport and metabolism) (Supplementary Figure S4).

About 10% of core genes with known COG functions (242 genes) were involved in “lipid transport and metabolism”, which ranked first amongst the COG categories. Lipid enrichment is known to be one of the important Mtb generalities, since most lipids are located in the Mtb cell envelope and account for ~40% of cellular dry mass (Forrellad et al., 2013; Jackson, 2014). They have also been shown to be closely related to some important Mtb generalities, such as high virulence, immune escape, cell invasion, and slow-growth (Forrellad et al., 2013). Approximately 9% of core genes (215 genes) were related to “energy production and conversion” (ranked second amongst COG categories). The energy-core-genes are associated with some Mtb generalities, such as the usage of host fatty acid as energy source, slow-growth, survival in the macrophage, and dormancy (Forrellad et al., 2013). “Secondary metabolite biosynthesis, transport, and catabolism” genes accounted for about 8% of the core genes; these mainly reflect the generality of intracellular Mtb “metabolic adaptations” (Jackson, 2014). In general, the core genes were primary genes, and are responsible for the generality of Mtb strains: lipid-rich cell envelopes, intracellular pathogenesis, slow-growth, and dormancy (Cole et al., 1998). Similarly, the 3,904 Mbo core genes were also enriched in similar COG categories (Supplementary Figure S4); this demonstrates some common features between the Mtb and Mbo strains (Forrellad et al., 2013).

### Core PE/PPE, VFs, and antigens determine some generalities of Mtb pathogenic phenotype (e.g., virulence, immune response)

Previous work has shown that PE/PPE, VFs, and antigens are associated with pathogenic Mtb phenotypes, such as virulence and immune response (Araujo et al., 2008; Akhter et al., 2012; Forrellad et al., 2013). Our pan-genomic analyses revealed 79 core PE/PPE genes (50% of total Mtb PE/PPE genes), 224 core virulence genes (77% of total Mtb virulence genes), and 380 core antigen genes (81% of total Mtb antigen genes) (Supplementary Figures S5A–C and Supplementary Table S5). Among these, 50 core virulence genes and 28 core antigen genes were enriched in the “Secondary metabolites biosynthesis, transport and catabolism” COG category; 21 core virulence genes and 21 core antigens were linked to the “Lipid transport and metabolism” COG category (Supplementary Figure S6); 14 core virulence genes and 25 core antigen genes were related to the “energy production and conversion” COG category (Supplementary Figure S6), and; 44 core PE/PPE genes were enriched in the “cell motility” COG category (Supplementary Figure S6). These COG functional enrichments reflect the main function/mechanism of pathogenic phenotype generality in Mtb. The control genomes (Mbo) showed almost identical patterns (Supplementary Figures S5D–F).

Further investigation revealed several important generalities of Mtb pathogenic phenotypes (Supplementary Table S6). First, 81 core PE/PPE, VF, and antigen genes are related to the thick, lipid-rich cell envelope phenotype of Mtb, including seven genes involved in maintaining the cell wall integrity and cell morphtype (*pknA, fbpC, hadC*, et al), 16 genes for host-cell entry (*pks1, Rv2954c, Rv2955c*, et al), and 32 genes associated with Mtb hyper virulence (*esxA, esxB, fadD29*, et al). “Thick, lipid-rich cell envelope” is known to be an important phenotypic characteristics of Mtb different from other bacterial pathogens (Forrellad et al., 2013). Secondly, 112 core PE/PPE, VF, and antigen genes are related to intracellular survival phenotype of Mtb, including 21 genes involved in stress response (*hsp, PE_PGRS11, devS*, et al), 18 genes affecting the antimicrobial activity of the phagosome (*ptpA, sapM, PPE10*, et al), 16 genes for host-cell entry (e.g., *mce1*A, *mce1B, mce1C*), and 16 genes involved in nutrient absorption (*Rv0203, pstS3, kdpE*, et al). Thirdly, 63 genes are relevant to the immune response phenotype of Mtb, such as five genes involved in host cytokine production (*lprA, lipC, PPE27*, et al), three genes associated with protective immunity (*PPE14, PE13, apa*). Fourthly, 8 core PE/PPE, VF, and antigen genes are related to dormancy phenotype of Mtb (e.g., *Rv0890c, Rv1734c, nuoG*).

### Super core genes (SCG) are of increased importance and reflect “super phenotype generality”

**Figure 3:**
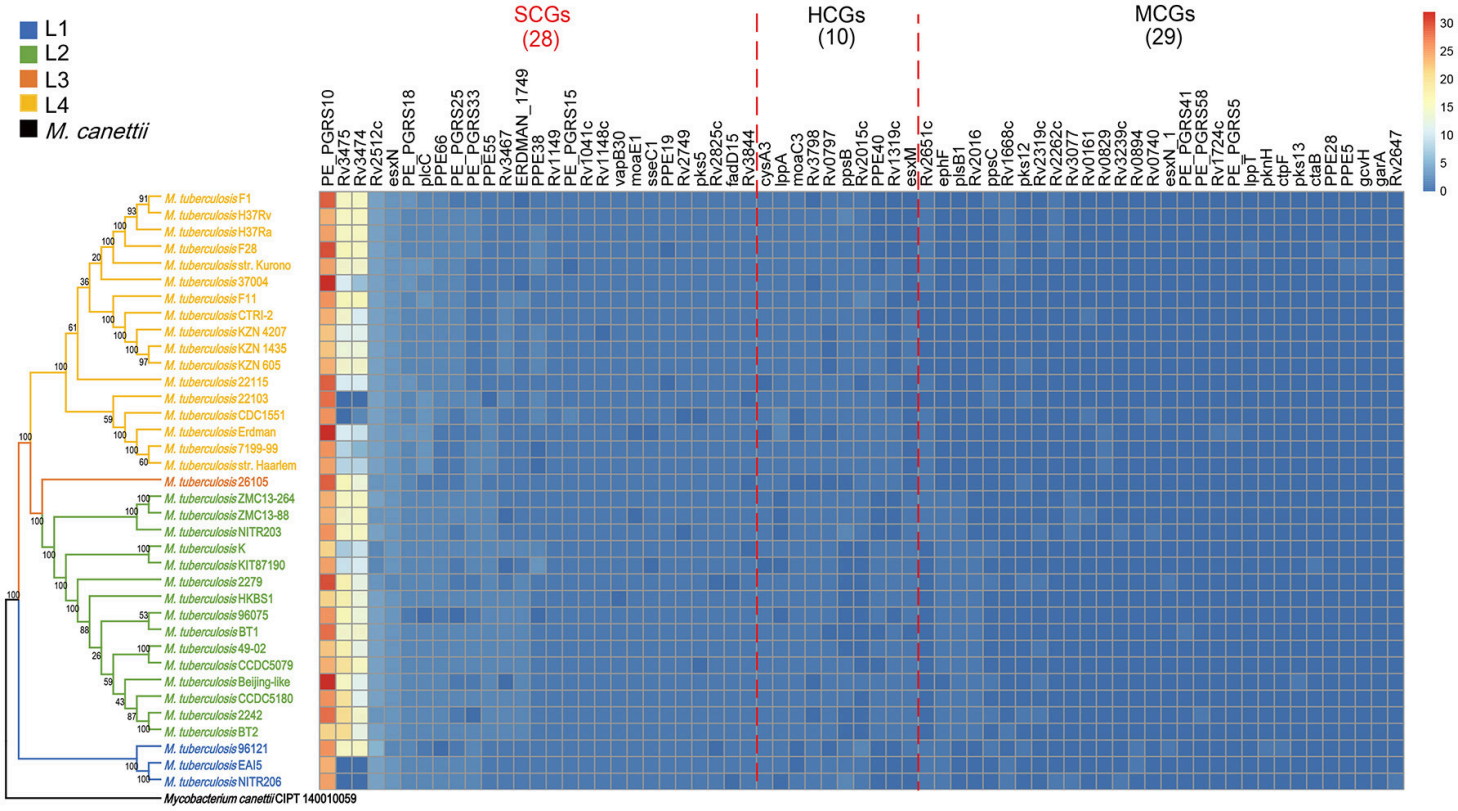
The three levels of multi-copy core genes in the 36 Mtb strains. Rows represent the 36 Mtb strains and columns represent the “multi-copy core genes” (i.e., core genes with more than one copy in at least one strain). The “multi-copy core genes” were classified into one of three levels: “super-core genes (SCGs)”, “high-level core genes (HCGs)”, or “middle-level core genes (MCGs)”. SCGs were defined as those core genes containing more than one copy in more than 90% of strains; HCGs were defined as those core genes containing more than one copy in 50–90% of strains, and; MCGs were defined as those core genes containing more than one copy in at least one strain. The color intensity indicates the copy number for each gene.

Since core genes are essential for survival and determine the generality of all strains of a species, multi-copy core genes are likely of increased importance and reflect “super generality”. By investigating the copy number of 3,679 Mtb core genes, we found 67 core genes that contained more than one copy in at least one strain (defined as “multi-copy core genes”). The 67 “multi-copy core genes” contained 28 “Super Core Genes” (SCGs: more than one copy in at least 90% strains), 10 “High-level Core Genes” (HCGs: more than one copy in 50%-90% strains), and 29 “Middle-level Core Genes” (MCGs: more than one copy in 0-50% strains) (Figure 3).

The 28 SCGs are likely of paramount importance and represent the key genetic factors of “super generality” of the Mtb phenotype. Most of the SCGs encode for PE/PPE, VFs, antigens, or transposases, which are known to play crucial roles in Mtb pathogenicity (Supplementary Table S7). First, nine SCGs encode for PE/PPE, most of which are secreted proteins that are closely related to Mtb virulence, immune response, and/or host-adaptation (Araujo et al., 2008; Akhter et al., 2012; Forrellad et al., 2013). Most importantly, *PE_PGRS33* (~3 copies per Mtb strain) is pleiotropic (i.e., it influences two or more phenotypic traits) (Mukhopadhyay and Balaji, 2011; Akhter et al., 2012). It is an antigen gene that is associated with Mtb immune response (Mukhopadhyay and Balaji, 2011; Forrellad et al., 2013), and also plays a role in Mtb cell structure for regulating growth status in macrophages (Akhter et al., 2012). *PPE19* (~2 copies per Mtb strain) is selectively highly expressed in macrophages; this was verified to facilitate the intracellular survival of Mtb (Dubnau and Smith, 2003). Secondly, two SCGs encoding for VFs (EsxN and PlcC) were proven to influence Mtb virulence. *esxN* (~ four copies per Mtb strain) is a core component of the ESX-5 secretion system, which is required for the secretion of most PE/PPE proteins and is critical for Mtb virulence (Ekiert and Cox, 2014). *plcC* (~3 copies per Mtb strain) can help Mtb escape from phagosomal vacuoles by disrupting the host membrane; this helps to improve survival in the host (Goudarzi et al., 2010; Forrellad et al., 2013). Thirdly, there were seven antigen SCGs, included four PE/PPE genes (*PE_PGRS10, PE_PGRS33, PPE19*, and *PPE55*) and one virulence gene (*esxN*); this suggests that these genes are pleiotropic. Fourthly, there were six SCGs that encode for transposases; these enhance the plasticity of the Mtb genome and contribute to pathogenesis through intra-/inter-species gene transfer (e.g., acquisition of a new virulence gene) (Bennett, 2004). In summary, these multiple functional PE/PPE, virulence, antigen, and transposase SCGs likely play extremely important roles in Mtb pathopoiesis and determine the “super phenotype generality” of Mtb strains.

Furthermore, the transcriptome expression data of SCGs downloaded from NCBI (Aguilar-Ayala et al., 2017) showed that almost all the SCGs (26) expressed both in exponential phase and stationary phase in H37Rv strains (Supplementary Table S8), indicating the importance of SCGs on the other hand.

**Figure 4:**
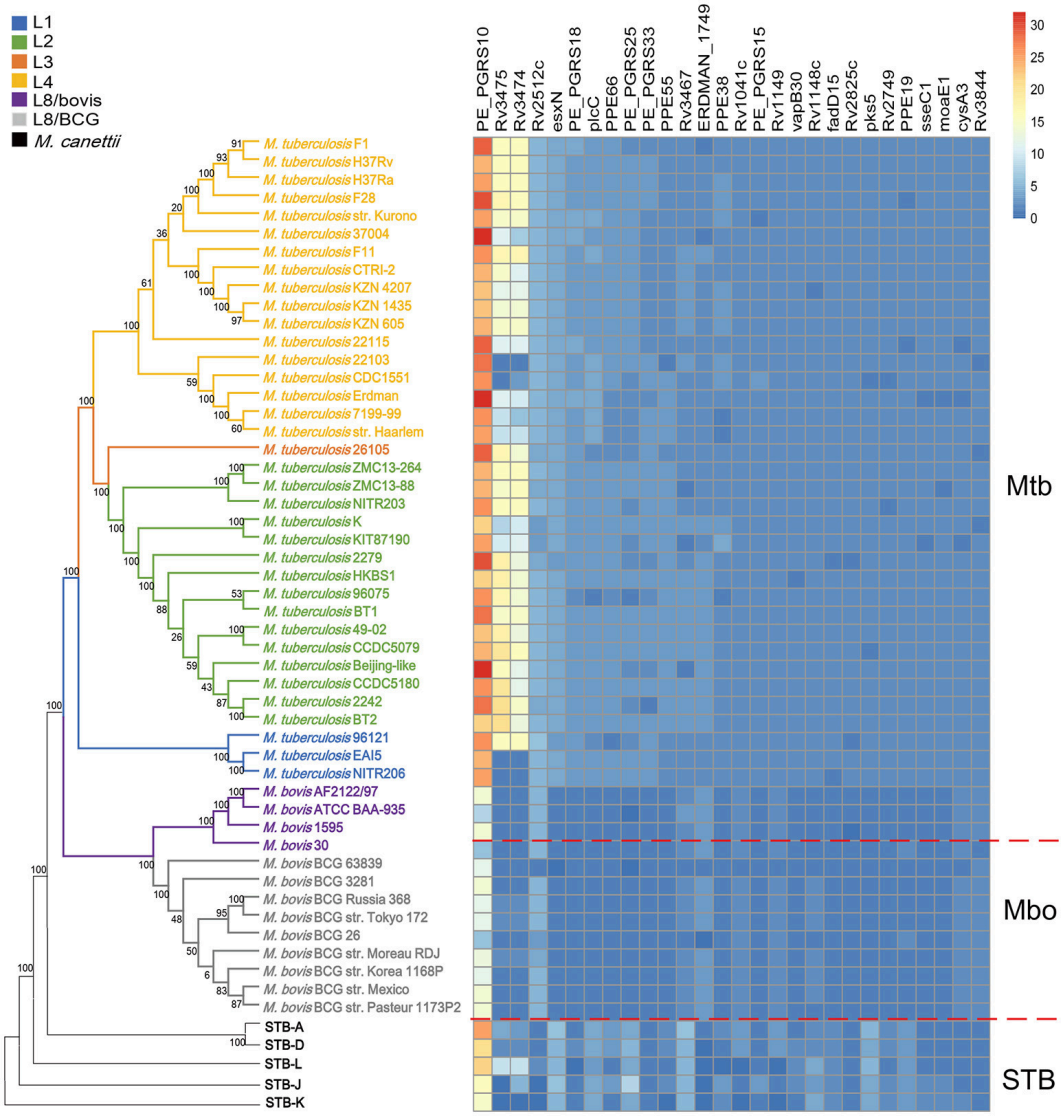
Schematic diagram showing the interconversion of the 28 Mtb SCGs during evolution. Rows represent the 36 Mtb strains, 13 Mbo strains, and 5 STB strains; columns represent the 28 Mtb SCGs. The color intensity indicates the copy number of each SCG.

In addition, we also found that there were more copies of most SCGs in Mtb than Mbo (20 out of 28) (Figure 4 and Supplementary Table S9). This might confer an advantage on Mtb strains in regards to adaptations to a human host, and determine some phenotypic differences between Mtb and Mbo strains. These differences might be attributed to natural selection during the long-term co-evolution of strains with human and cattle (Brosch et al., 2002; Galagan, 2014). Most notably, the average copy number of the super core antigen gene *PE_PGRS10* was ~26 per Mtb strain, but only ~12 per Mbo strain (Supplementary Figure S7). These differences in average copy numbers of SCGs might contribute to the differences in phenotype between Mtb and Mbo strains.

### Mtb-specific core-genes determine its individuality

We compared the core genes of Mtb and Mbo to explore the genetic basis for differences in individuality between Mtb and Mbo. We identified 48 Mtb and 19 Mbo specific single-copy core genes that to some extent determine the differences in individuality between Mtb and Mbo (Supplementary Figure S8, Supplementary Tables S10, S11).

The 48 Mtb-specific single-copy core genes contained 23 PE/PPE, virulence, and antigen genes (19 in RDs) (Supplementary Table S10 and Supplementary Figure S9), reflecting the core individuality of Mtb different from Mbo. Importantly, most of Mtb specific single-copy core genes (40/48) were located in the “region of difference (RD)”, which has been showed to result in the phenotypic difference between Mtb and Mbo (Jia et al., 2017). For example, there were 10 Mtb specific RD4 core genes; RD4 has been proven to play an important role in mycobacterial virulence (Ru et al., 2017). The 13 Mtb-specific RD7 core genes contained eight mammalian cell entry 3 (mce3) family genes. RD7 is important for bacterial entry and survival in the host, and is closely related to the adaptation to human hosts (Jia et al., 2017). Five Mtb-specific RD11 core genes contained a T cell antigen gene (*Rv2654c*), which encodes a strongly recognized T cell antigen that is highly specific for human TB (Aagaard et al., 2004). Four Mtb-specific RD8 core genes contained co-transcribed and co-translated PE32 and PPE65 antigens; these antigens modulate host protective immune responses by suppressing Th1 cells and cytokines, and result in adaptations to human hosts (Khubaib et al., 2016). In addition, an Mtb-specific core gene, *pknD*, was not located in any RDs; this gene is required for Mtb survival and persistence inside human hosts (Forrellad et al., 2013). It plays an essential role in the invasion of the brain endothelia, and could transduce environmental signals during infections by regulating the expression of some adaptive genes (Skerry et al., 2013). In summary, the 48 Mtb-specific single-copy core genes reflect the core individuality of Mtb different from Mbo. The different individualities are considered to be the results of long-term co-evolution of *Mycobacterium tuberculosis* complex (MTBC) strains with humans and cattle (Brosch et al., 2002); thus, the absence of these Mtb-specific PE/PPE, virulence, and/or antigen genes in Mbo strains could partially explain the differences in host-adaptation individualities.

### Dispensable genes are secondary genes and determine the partially shared features of Mtb

To investigate the genetic basis of the partially shared features of Mtb strains, the 1,122 Mtb dispensable genes were also assigned to COG categories (Tatusov et al., 1997). Compared to the Mbo strains, Mtb strains contained higher proportion of genes in six COG categories that reflect the secondary and partially shared features of Mtb strains (category I: Lipid transport and metabolism; category M: cell wall/membrane/envelope biogenesis; category T: signal transduction mechanisms; category Q: Secondary metabolites biosynthesis, transport and catabolism; category H: coenzyme transport and metabolism; category P: inorganic ion transport and metabolism) (Supplementary Figure S10). Notably, the higher proportion of dispensable genes in the “cell wall/membrane/envelope biogenesis” and “lipid transport and metabolism” categories in Mtb strains might reflect the “human adaptation” feature due to co-evolution of partial Mtb strains with humans in recent times (Kinsella et al., 2003; Forrellad et al., 2013). For example, some drug-resistance strains contain thicker cell envelopes with more lipids, which are an adaptation to the use of antibiotics (Velayati et al., 2009). If we continue to extensively use antibiotics, it is reasonable to infer that these lipid-dispensable genes may become core genes. In addition, the higher proportion of metabolism-related dispensable genes (Category Q, H and P) might indicate new partially shared metabolic features of Mtb and the ability to adapt to fast-changing conditions in modern human hosts (García-Alix et al., 2017). In conclusion, dispensable genes were secondary genes that appeared in a subset of Mtb strains, and determined partially shared features. Their appearance and/or disappearance in a subset of Mtb strains might be responsible for adaptations to the host and/or environmental conditions (Medini et al., 2005; Vernikos et al., 2015).

Although dispensable genes are secondary genes, they are important for the adaptation of lineage-strains to certain hosts and circumstances; thus, they could possibly be used to distinguish amongst different lineages. Mtb strains from two major modern lineages, lineages 2 and 4, are more infectious than those from the other lineages; these two lineages are also responsible the majority of the infected population worldwide (Coscolla and Gagneux, 2014). The pan-genome analysis identified five L2-specific genes (*dosTa, dosTb, PPE57a, RN10_0073*, and *RN10_0122*) and three L4-specific genes (*mmpL13a, Rv0325*, and *Rv2292c*) from 1,122 dispensable Mtb genes (Supplementary Table S12); this could partially indicate the individuality of the L2 and L4 lineages. This might also be the result of the co-evolution of Mtb with different human populations from different geographic areas (Galagan, 2014).

### Strain-specific genes reflect the individuality of Mtb strains

The strain-specific genes are mainly enriched in the following COG categories (Supplementary Table S13 and Supplementary Figure S11), reflecting the different individuality of the 36 Mtb strains: 30 strain-specific genes of eight strains (37004, 96075, CTRI-2, NITR206, F1, F11, F28, KZN 1435) are enriched in “Cell wall/membrane/envelope biogenesis” and “Lipid transport and metabolism” COG categories, suggesting the thick, lipid-rich cell envelope phenotype individuality of these strains; 15 strain-specific genes of four strains (9612, EAI5-NITR206, Erdman, KZN 605) are enriched in “Replication, recombination and repair” COG category, indicating the improved anti-stress capability of these strains; 23 strain-specific genes of nine strains (2279, 26105, BT2, CCDC5079, CTRI-2, Erdman, F28, HKBS1, ZMC13-264) are primarily related to “Secondary metabolites biosynthesis, transport and catabolism” and “Energy production and conversion” COG categories, suggesting the increased intracellular survivability since they might efficiently utilize the host nutrition to generate energy; 35 strain-specific genes of eight strains (96121, 323, NITR203, BT2, CDC1551, F1, KZN-605, ZMC13-264) are enriched in “Transcription” and “Amino acid transport and metabolism” COG categories, implying the higher ability to synthesize protein *in vivo* for these strains.

## Discussion

Since the core genes are primary genes and determine the phenotype generality, the 28 super core genes (SCGs) should be of increased importance and should reflect the “super phenotype generality” of Mtb strains. To explore the SCGs, we studied the evolution of SCGs, mainly focusing on the CNVs and selection pressure, to determine if the SCGs are stable during evolution and if they can be turned into the other kinds of genes (e. g., single-copy core gene, dispensable gene, and even strain-specific gene).

### The interconversion among SCGs, single-copy core genes, dispensable genes, and strain-specific genes through CNVs during evolution

**Figure 5:**
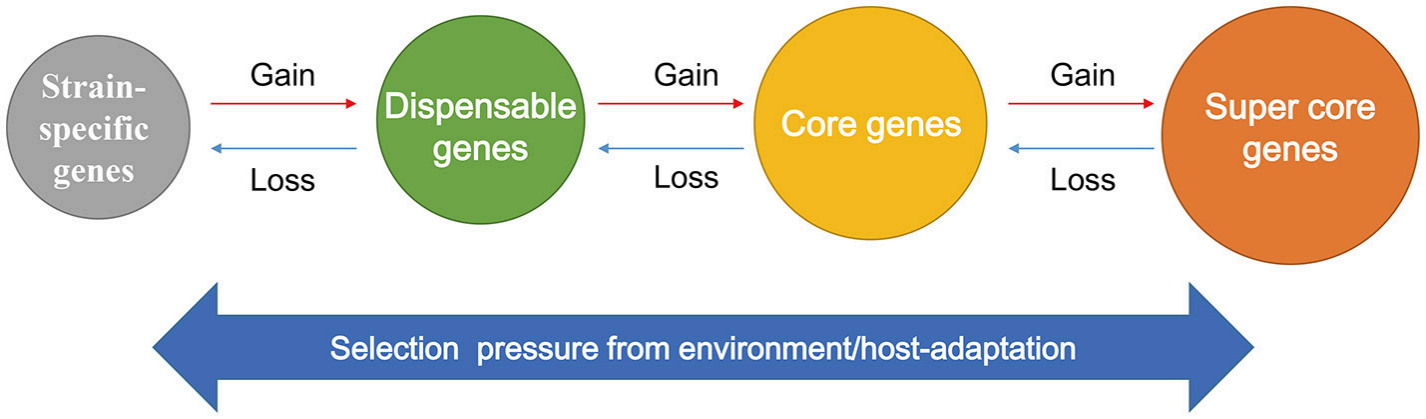
Schematic diagram showing the interconversion of SCGs, core genes, dispensable genes, and strain-specific genes following evolution under different conditions. The “interconversion” means that the strain-specific genes, dispensable genes, core genes and SCGs may be transformed into one other during evolution through CNVs. The increases in gene copies (Gain) lead to the conversion from strain-specific genes to dispensable genes, to core genes, and to SCGs. In contrast, the decreases in gene copies (Loss) result in the transformation from SCGs to core genes, to dispensable genes, and to strain-specific genes. Previous research indicated that the interconversion among these genes might be driven by the selection pressure from environment/host adaptation.

Further investigation of the 28 SCGs indicated interconversion amongst SCGs, single-copy core genes, dispensable genes, and strain-specific genes through CNVs (Figure 4), revealing the dynamic changes in gene copy numbers during evolution. This also reflects the changes of importance derived from host adaptations (Jia et al., 2017). Specially, during the evolution of STB (the putative ancestor of MTBC) (Galagan, 2014) to Mbo and Mtb, some single-copy core genes, dispensable genes, and even strain-specific genes could have been converted into SCGs by acquiring additional copies. For example, the STB single-copy core gene *Rv3844* was converted into a SCG in both Mtb and Mbo by gaining an additional copy; the STB dispensable genes *Rv3475* and *Rv3474* became MCGs and SCGs in Mbo and Mtb, respectively. Interestingly, one STB strain-specific gene, *ERDMAN_1749*, was transformed a dispensable gene and SCG in Mbo and Mtb, respectively. In contrast, some STB and Mtb SCGs became single-copy core genes or even dispensable genes in Mbo due to a loss of copies. For example, *plcC* was a SCG in both STB and Mtb, but is a single-copy core gene in Mbo. *PE_PGRS25* contained 3~5 copies in STB and Mtb strains, but was a dispensable gene in Mbo due to deletions in some Mbo strains. In summary, SCGs, single-copy core genes, dispensable genes, and strain specific genes can be transformed into one other during evolution (Figure 5); this highlights the changes of importance due to host adaptation.

Two Mtb SCGs (*PE_PGRS33* and *fadD15*) were selected for deeper investigations of the evolutionary process of SCGs. The results suggest that host-adaptation is the main driving force of the CNV. First, we constructed the phylogenetic tree of *PE_PGRS33*. It has three copies and exists in different MTBC lineages (Supplementary Figure S12), indicating different host adaptations. Specially, *PE_PGRS33_1* was present in all MTBC strains, but it was the only *PE_PGRS33* copy in the older *M. canettii* and animal-adapted MTBC strains (L8 lineage). This suggests that *PE_PGRS33_1* may have first appeared in the three copies and inherited from common ancestor (Gey van Pittius et al., 2006). *PE_PGRS33_2* was only present in modern human-associated MTBC strains (Mtb and *M. africanum*), indicating the modern human-adaptation. The third copy, *PE_PGRS33_3*, was present in most of the Mtb strains, this implies that it have appeared as an adaptation to non-African hosts following the human migration out of Africa (Supplementary Figure S12B; Comas et al., 2013). Therefore, the change in *PE_PGRS33* copy number (from a single-copy core gene in STB and Mbo strains to a SCG in Mtb strains) was likely due to host-adaptation from the co-evolution of MTBC strains and different hosts. We could also infer the order of appearance of *PE_PGRS33_1, 2*, and *3* based on the appearance of the host (Comas et al., 2013; Galagan, 2014).

We then constructed the phylogenetic tree of *fadD15*. The investigation into Mtb *fadD15* SCG revealed that its copy number increased over the evolution from fast-growing, non-pathogenic mycobacteria to slow-growing, pathogenic mycobacteria (Gey van Pittius et al., 2006; Supplementary Figure S13). *fadD15* was a single-copy core gene in fast-growing mycobacteria, but was a multi-copy core gene in slow-growing mycobacteria. This change could be due to environmental/host-adaptation, and is also closely related to pathogenesis since most fast-growing, non-pathogenic mycobacteria are found in the environment and most slow-growing, pathogenic mycobacteria are found in human/animal cells (Gey van Pittius et al., 2006; Galagan, 2014). *fadD15_1* was found in all mycobacteria species (i.e., both fast- and slow-growing species); this suggests that it initially appeared among the four copies and was inherited from common ancestor (Gey van Pittius et al., 2006). It may be irrelevant for pathogenesis. *fadD15_2* was present specifically in slow-growing mycobacteria (except Mbo, *M. leprae*, and *M. haemophilum*); this implies that it might be related to pathogenesis. Here we could infer that *fadD15_2* appeared after *fadD15_1* as an adaptation of the later emerging, slow-growing mycobacteria (Sandegren and Andersson, 2009; Galagan, 2014). *fadD15_3* (found in *M. avium, M. kansasii, M. marinum*, and *M. ulcerans*) and *fadD15_4* (found in *M. kansasii*) only exist in partial slow-growing mycobacteria, indicating they appeared more recently in specific slow-growing mycobacteria.

Further analysis showed that *fadD15_1* and *fadD15_2* possess lineage-specific amino acid substitutions (Supplementary Figure S14). In FadD15_1, I103T is only present in the L4 lineage; E382G is specific to the L2, L3, and L4 lineages (Supplementary Figure S14A). There are two substitutions (E232A and A460V) to FadD15_2 that are only found in L1 strains; the P492T substitution occurred when *M. canettii* evolved to the L1, L2, L3, L4, L6, and L8 lineage strains (Supplementary Figure S14B). These mutations might have emerged along with the differentiation of lineages, and may be associated with lineage-adaptation.

In addition, previous research has also described the process of copy number increase in ESX gene-clusters during the evolution of mycobacteria (Gey van Pittius et al., 2006). The ESX-4 gene-cluster-region first appeared in the Gram-positive actinobacteria with high GC content (e.g. *N. farcinica, C. glutamicum, C. efficiens, C. jeikeium*, and *C. diphtheriae*) during the origin of actinobacteria. Then the ESX-1, ESX-3, and ESX-2 gene-cluster-regions sequentially occurred in the fast-growing, non-pathogenic mycobacteria (e.g., *M. smegmatis, M. flavescens, M. vanbaalenii, M. sp. MCS* and *JLS and M. sp. KMS*) through the successive duplication events in the ESX-4 gene-cluster-region during mycobacteria evolution. At last, the ESX-5 gene-cluster-region appeared in the slow-growing, pathogenic mycobacteria (e.g. *M. microti, M. bovis, M. tuberculosis, M. africanum, M. marinum* and *M. ulcerans*) by doubling the ESX-2 gene-cluster-region in the late period of mycobacteria evolution. The CNV might be both the cause and consequence of natural selection.

Our in-depth analysis of SCGs copy numbers revealed that CNV plays important roles in host-adaptation; conversely, host-adaptation (i.e., co-evolution with hosts) had led to CNV in the strains. The analysis also indicates the changes of gene importance through CNV during evolution. In addition, the mutations of different copies were more specific to individual strains and lineages.

### Selection pressure derived from host-adaptation leads to the interconversion among different kinds of genes through CNVs

**Figure 6:**
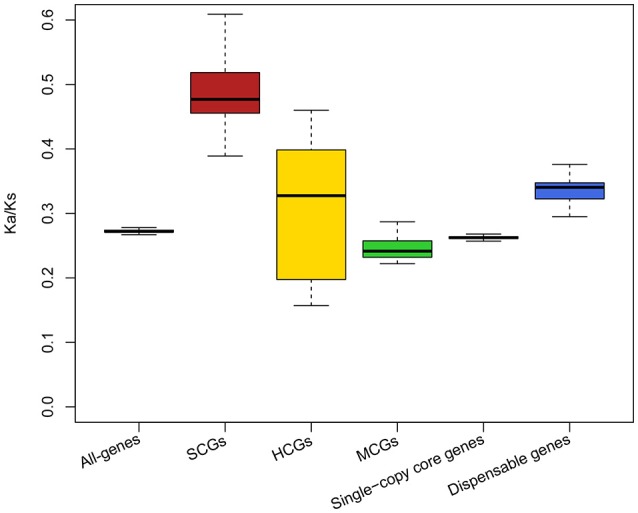
Ka/Ks ratios for all types of genes in Mtb strains. The schematic diagram shows the Ka/Ks ratios for all-genes, SCGs, HCGs, MCGs, single-copy core genes, and dispensable genes in Mtb strains. Different colors indicate different kinds of genes: all-genes, gray; SCGs, red; HCGs, yellow; MCGs, green; single-copy core genes, purple; dispensable genes, blue. The boxplots show the median values.

To investigate the impact of selection pressure on CNVs, we analyzed the rate of non-synonymous and synonymous substitutions (Ka/Ks) for different kinds of Mtb genes (i.e., SCGs, HCGs, MCGs, single-copy core genes, and dispensable genes). This analysis revealed that SCGs and HCGs were subject to higher selection pressures (i.e., higher Ka/Ks values) than single-copy core genes (Figure 6); this indicates that they are undergoing adaptive evolution (Thakur et al., 2013). These genes could gain new functionalities through non-synonymous substitutions on different copies, allowing them to become pleiotropic (Kiwuwa et al., 2013; Galagan, 2014). In this way, the SCGs and HCGs with multiple copies may serve to the enhancement of host-adaptation.

In general, the copy expansion of microbial genes is mainly derived from duplication and/or horizontal gene transfer (HGT) (Kinsella et al., 2003; Bratlie et al., 2010). The additional copies might endow SCGs and HCGs with new properties and functions via non-synonymous substitutions; therefore, different copies of SCGs and HCGs could be subject to different selection pressures. Our results support this notion. There were three copies of the SCG *PPE66* in Mtb; two of them had lower Ka/Ks values (0.27), but the other had a higher Ka/Ks value (0.99). The copy with the higher Ka/Ks value was likely undergoing adaptive evolution, which might confer new properties/functions on this copy and distinguish it from the other two copies.

Interestingly, the dispensable genes displayed relatively high selection pressures (Ka/Ks value) (Figure 6), indicating that they were undergoing adaptive evolution. Higher selection pressures could allow dispensable genes to gradually dominate the Mtb strains and eventually become core genes. However, if the selection pressure due to the ever-changing environment and/or host exceeded the tolerance of dispensable genes, the dispensable genes would likely reduce or even disappear.

In conclusion, the selection pressure derived from the environment/host led to changes in gene importance, as well as the interconversion amongst different kinds of genes through CNVs during evolution. Genes with higher selection pressures could follow one of two paths: if they become better adapted through the accumulation of additional copies or proper non-synonymous substitutions (derived from high selection pressure), they would likely be retained and could even convert to more important gene categories (convert from strain-specific genes to dispensable genes convert from HCGs to SCGs); on the other hand, if they do not adjust to the environment/host through the accumulation of more harmful non-synonymous substitutions, they would likely taper off or even disappear (i.e., reverse transformation). Thus, CNV might be both the cause and consequence of natural selection (Bratlie et al., 2010; Hardwick et al., 2014).

## Conclusions

We conducted a pan-genome analysis of Mtb genomes, so as to investigate which are primary/secondary, which are responsible for phenotypic generality/individuality, and which interconvert during evolution. (1) We identified 3,679 Mtb core (i.e., primary) genes, determining their phenotypic generality (e.g., virulence, slow growth, dormancy). (2) We detected 48 Mtb specific single core genes that partially reflect the differences between Mtb and Mbo individuality. (3) We observed 1,122 dispensable and 964 strain-specific secondary genes, reflecting partially shared and lineage-/strain-specific individualities. (4) We found five L2 lineage-specific genes that might be related to the increased virulence of the L2 lineage. (5) We discovered 28 Mtb “Super Core Genes” (SCGs), which might be of increased importance, and reflected the “super phenotype generality.” Most SCGs encode PE/PPE, virulence factors, antigens, and transposases, and have been verified as playing crucial roles in Mtb pathogenicity. (6) There are interconversion among SCGs, single-copy core, dispensable, and strain-specific genes through CNVs during evolution; different mutations on different copies highlight the delicate adaptive-evolution regulation amongst Mtb lineages. (7) The interconversion reflects that the importance of genes varied through CNVs, which might be driven by selective pressure from environment/host-adaptation.

## Author contributions

TY, JZO, HH, and FC designed the study. TY, JZO, XY, GM, GW, QL, YJS, and YHS performed bioinformatics analyses. TY, JZO, JZA, CL, JX, XJ, ND, LY, and FC prepared the manuscript. All authors contributed to and approved the final manuscript.

### Conflict of interest statement

The authors declare that the research was conducted in the absence of any commercial or financial relationships that could be construed as a potential conflict of interest.

## Abbreviations

CDS: coding sequences
CNV: copy number variation
COG: Clusters of Orthologous Group
HGT: horizontal gene transfer
HCG: High-level Core Gene
IEDB: Immune Epitope Database
MAFFT: Multiple Alignment using Fast Fourier Transform
Mbo: Mycobacterium bovis
MCG: Middle-level Core Gene
Mtb: Mycobacterium tuberculosis
MTBC: Mycobacterium tuberculosis complex
PanGP: Pan-Genome Profile Analyze Tool
PE/PPE: proline-glutamate/proline-proline-glutamate
PGAP: pan-genome analysis pipeline
RAST: Rapid Annotation using Subsystem Technology
RD: region of difference
SCG: Super Core Gene
STB: smooth tubercle bacilli
TB: tuberculosis
VF: virulence factor
VFDB: Virulence Factor database
WHO: World Health Organization.
