# Supplementary material for: Pan-Genomic Study of Mycobacterium tuberculosis Reflecting the Primary/Secondary Genes, Generality/Individuality, and the Interconversion Through Copy Number Variations
Source: Front Microbiol. 2018 Aug 17;9:1886. doi: 10.3389/fmicb.2018.01886 (PMC6109687; doi:10.3389/fmicb.2018.01886)
Supplement: Supplementary file 2 [file Table_2.DOCX]

Supplementary Table S2. General information on the 13 Mbo strains.

| **Strains** | **Accession No.** | **ATCC No./Lineage** | **Genome size (Mb)** | **GC content (%)** | **Coding gene No.** | **% Coding region** | **Source [reference]** |
| --- | --- | --- | --- | --- | --- | --- | --- |
| *M. bovis* ATCC BAA-935 | NZ_CP009449 | L8 | 4.36 | 65.6 | 4338 | 90.18 | American Type Culture Collection |
| *M. bovis* AF2122/97 | NC_002945 | L8 | 4.35 | 65.6 | 4286 | 90.58 | (Garnier et al., 2003) |
| *M. bovis* 30 | CP010332 | L8 | 4.34 | 65.6 | 4312 | 89.9 | Beijing Institute of Genomics (Zhu et al., 2016) |
| *M. bovis* 1595 | NZ_CP012095 | L8 | 4.35 | 65.6 | 4275 | 90.5 | Animal and Plant Quarantine Agency, Korea (Kim et al., 2015) |
| *M. bovis* BCG Pasteur 1173P2 | NC_008769 | L8 | 4.37 | 65.6 | 4281 | 90.37 | Institute Pasteur (Brosch et al., 2007) |
| *M. bovis* BCG 63839 | NZ_CP003494 | L8 | 4.33 | 65.7 | 4350 | 90.05 | Chinese Academy of Sciences (Brosch et al., 2007; Pan et al. 2011) |
| *M. bovis* BCG Korea 1168P | NC_020245 | L8 | 4.38 | 65.6 | 4286 | 90.45 | Korean Institute of Tuberculosis (Joung et al., 2013) |
| *M. bovis* BCG Mexico | NC_016804 | L8 | 4.35 | 65.7 | 4263 | 90.47 | Universidad Nacional Autonoma de Mexico (Orduna et al., 2011) |
| *M. bovis* BCG Moreau RDJ | NZ_AM412059 | L8 | 4.34 | 65.6 | 4263 | 90.41 | Institute Oswaldo Cruz – Fiocruz (Gomes et al., 2011) |
| *M. bovis* BCG Tokyo 172 | NC_012207 | L8 | 4.37 | 65.6 | 4306 | 90.48 | Masaaki Seki Japan BCG Laboratory (Seki et al., 2009) |
| *M. bovis* BCG 3281 | NZ_CP008744 | L8 | 4.41 | 65.6 | 4325 | 90.43 | Chinese Academy of Agriculture |
| *M. bovis* BCG Russia 368 | NZ_CP009243 | L8 | 4.37 | 65.6 | 4304 | 90.47 | Gamaleya Institute for Epidemiology and Microbiology (Voronina et al., 2016) |
| *M. bovis* BCG 26 | CP010331 | L8 | 4.35 | 65.65 | 4348 | 90.31 | Beijing Institute of Genomics (Zhu et al., 2016) |

**References**

Brosch, R., Gordon, S. V., Garnier, T., Eiglmeier, K., Frigui, W., Valenti, P., et al. (2007). Genome plasticity of BCG and impact on vaccine efficacy. *Proc. Natl. Acad. Sci. U.S.A*. 104, 5596-5601. doi: 10.1073/pnas.0700869104.

Garnier, T., Eiglmeier, K., Camus, J. C., Medina, N., Mansoor, H., Pryor, M., et al. (2003). The complete genome sequence of *Mycobacterium bovis*. *Proc. Natl. Acad. Sci. U.S.A*. 100, 7877-7882. doi: 10.1073/pnas.1130426100.

Gomes, L. H., Otto, T. D., Vasconcellos, E. A., Ferrao, P. M., Maia, R. M., Moreira, A. S., et al. (2011). Genome sequence of *Mycobacterium bovis* BCG Moreau, the Brazilian vaccine strain against tuberculosis. *J. Bacteriol*. 193, 5600-5601. doi: 10.1128/JB.05827-11.

Joung, S. M., Jeon, S. J., Lim, Y. J., Lim, J. S., Choi, B. S., Choi, I. Y., et al. (2013). Complete genome sequence of *Mycobacterium bovis* BCG Korea, the Korean vaccine strain for substantial production. *Genome. Announc*. 1, e0006913. doi: 10.1128/genomeA.00069-13.

Kim, N., Jang, Y., Kim, J. K., Ryoo, S., Kwon, K. H., Kang, S. S., et al. (2015). Complete genome sequence of *Mycobacterium bovis* clinical strain 1595, isolated from the laryngopharyngeal lymph node of South Korean cattle. *Genome. Announc*. 3. doi: 10.1128/genomeA.01124-15.

Orduna, P., Cevallos, M. A., de Leon, S. P., Arvizu, A., Hernandez-Gonzalez, I. L., Mendoza-Hernandez, G., et al. (2011). Genomic and proteomic analyses of *Mycobacterium bovis* BCG Mexico 1931 reveal a diverse immunogenic repertoire against tuberculosis infection. *BMC. Genomics*. 12, 493. doi: 10.1186/1471-2164-12-493.

Pan, Y., Yang, X., Duan, J., Lu, N., Leung, A. S., Tran, V., et al. (2011). Whole-genome sequences of four *Mycobacterium bovis* BCG vaccine strains. *J. Bacteriol.* 193, 3152-3153. doi: 10.1128/JB.00405-11.

Seki, M., Honda, I., Fujita, I., Yano, I., Yamamoto, S., and Koyama, A. (2009). Whole genome sequence analysis of *Mycobacterium bovis* Bacillus Calmette-Guerin (BCG) Tokyo 172: A comparative study of bcg vaccine substrains. *Vaccine*. 27, 1710-1716. doi: 10.1016/j.vaccine.2009.01.034.

Voronina, O. L., Kunda, M. S., Aksenova, E. I., Semenov, A. N., Ryzhova, N. N., Lunin, V. G., et al. (2016). Mosaic structure of *Mycobacterium bovis* BCG genomes as a representation of phage sequences' mobility. *BMC. Genomics*. 17, 1009. doi: 10.1186/s12864-016-3355-1.

Zhu, L. X., Zhong, J., Jia, X. M., Liu, G., Kang, Y., Dong, M. X., et al. (2016). Precision methylome characterization of *Mycobacterium tuberculosis* complex (MTBC) using PacBio single-molecule real-time (SMRT) technology. *Nucleic. Acids. Res*. 44, 730-743. doi: 10.1093/nar/gkv1498.
