# Supplementary material for: Pan-Genomic Study of Mycobacterium tuberculosis Reflecting the Primary/Secondary Genes, Generality/Individuality, and the Interconversion Through Copy Number Variations
Source: Front Microbiol. 2018 Aug 17;9:1886. doi: 10.3389/fmicb.2018.01886 (PMC6109687; doi:10.3389/fmicb.2018.01886)
Supplement: Supplementary file 3 [file Table_3.DOCX]

Supplementary Table S3. The number and proportion of core, dispensable, and strain-specific genes in the 36 Mtb strains.

| **Strains** | **Lineage** | **Core genes No.** | **Dispensable genes No.** | **Strain-specific genes No.** | **% Core genes** | **% Dispensable genes** | **% Strain-specific genes** |
| --- | --- | --- | --- | --- | --- | --- | --- |
| *M. tuberculosis* F1 | L4 | 3779 | 581 | 40 | 85.89 | 13.2 | 0.91 |
| *M. tuberculosis* F28 | L4 | 3785 | 559 | 22 | 86.69 | 12.8 | 0.5 |
| *M. tuberculosis* H37Ra | L4 | 3778 | 560 | 10 | 86.89 | 12.88 | 0.23 |
| *M. tuberculosis* Erdman | L4 | 3772 | 565 | 36 | 86.26 | 12.92 | 0.82 |
| *M. tuberculosis* 22103 | L4 | 3751 | 555 | 39 | 86.33 | 12.77 | 0.9 |
| *M. tuberculosis* 22115 | L4 | 3769 | 554 | 33 | 86.52 | 12.72 | 0.76 |
| *M. tuberculosis* 37004 | L4 | 3769 | 561 | 45 | 86.15 | 12.82 | 1.03 |
| *M. tuberculosis* KZN 4207 | L4 | 3765 | 552 | 7 | 87.07 | 12.77 | 0.16 |
| *M. tuberculosis* KZN 605 | L4 | 3774 | 535 | 17 | 87.24 | 12.37 | 0.39 |
| *M. tuberculosis* KZN 1435 | L4 | 3770 | 544 | 19 | 87.01 | 12.55 | 0.44 |
| *M. tuberculosis* Haarlem | L4 | 3763 | 546 | 13 | 87.07 | 12.63 | 0.3 |
| *M. tuberculosis* F11 | L4 | 3781 | 562 | 9 | 86.88 | 12.91 | 0.21 |
| *M. tuberculosis* H37Rv | L4 | 3777 | 557 | 2 | 87.11 | 12.85 | 0.05 |
| *M. tuberculosis* CDC1551 | L4 | 3753 | 543 | 55 | 86.26 | 12.48 | 1.26 |
| *M. tuberculosis* 7199-99 | L4 | 3761 | 575 | 8 | 86.58 | 13.24 | 0.18 |
| *M. tuberculosis* CTRI-2 | L4 | 3769 | 550 | 12 | 87.02 | 12.7 | 0.28 |
| *M. tuberculosis* Kurono | L4 | 3779 | 550 | 13 | 87.03 | 12.67 | 0.3 |
| *M. tuberculosis* 26105 | L3 | 3772 | 573 | 48 | 85.86 | 13.04 | 1.09 |
| *M. tuberculosis* 2242 | L2 | 3787 | 580 | 61 | 85.52 | 13.1 | 1.38 |
| *M. tuberculosis* 2279 | L2 | 3782 | 561 | 57 | 85.95 | 12.75 | 1.3 |
| *M. tuberculosis* NITR203 | L2 | 3782 | 587 | 73 | 85.14 | 13.21 | 1.64 |
| *M. tuberculosis* HKBS1 | L2 | 3777 | 557 | 9 | 86.97 | 12.83 | 0.21 |
| *M. tuberculosis* CCDC5079 | L2 | 3783 | 561 | 10 | 86.89 | 12.88 | 0.23 |
| *M. tuberculosis* 49-02 | L2 | 3779 | 569 | 2 | 86.87 | 13.08 | 0.05 |
| *M. tuberculosis* 96075 | L2 | 3771 | 541 | 15 | 87.15 | 12.5 | 0.35 |
| *M. tuberculosis* BT1 | L2 | 3780 | 542 | 15 | 87.16 | 12.5 | 0.35 |
| *M. tuberculosis* BT2 | L2 | 3781 | 548 | 14 | 87.06 | 12.62 | 0.32 |
| *M. tuberculosis* CCDC5180 | L2 | 3783 | 566 | 4 | 86.91 | 13 | 0.09 |
| *M. tuberculosis* 323 | L2 | 3783 | 575 | 45 | 85.92 | 13.06 | 1.02 |
| *M. tuberculosis* ZMC13-88 | L2 | 3775 | 571 | 18 | 86.5 | 13.08 | 0.41 |
| *M. tuberculosis* ZMC13-264 | L2 | 3778 | 571 | 16 | 86.55 | 13.08 | 0.37 |
| *M. tuberculosis* KIT87190 | L2 | 3764 | 552 | 25 | 86.71 | 12.72 | 0.58 |
| *M. tuberculosis* K | L2 | 3763 | 561 | 10 | 86.83 | 12.94 | 0.23 |
