# Supplementary material for: Pan-Genomic Study of Mycobacterium tuberculosis Reflecting the Primary/Secondary Genes, Generality/Individuality, and the Interconversion Through Copy Number Variations
Source: Front Microbiol. 2018 Aug 17;9:1886. doi: 10.3389/fmicb.2018.01886 (PMC6109687; doi:10.3389/fmicb.2018.01886)
Supplement: Supplementary file 4 [file Table_4.DOCX]

Supplementary Table S4. The number and proportion of core, dispensable, and strain-specific genes in the 13 Mbo strains.

| **Strains** | **Lineage** | **Core genes No.** | **Dispensable genes No.** | **Strain-specific genes No.** | **% Core genes** | **% Dispensable genes** | **% Strain-specific genes** |
| --- | --- | --- | --- | --- | --- | --- | --- |
| *M. bovis* ATCC BAA-935 | L8 | 3982 | 288 | 68 | 91.79 | 6.64 | 1.57 |
| *M. bovis* AF2122/97 | L8 | 3930 | 349 | 7 | 91.69 | 8.14 | 0.16 |
| *M. bovis* 30 | L8 | 3922 | 347 | 43 | 90.96 | 8.05 | 1 |
| *M. bovis* 1595 | L8 | 3930 | 329 | 16 | 91.93 | 7.7 | 0.37 |
| *M. bovis* BCG Pasteur 1173P2 | L8 | 3990 | 274 | 17 | 93.2 | 6.4 | 0.4 |
| *M. bovis* BCG 63839 | L8 | 3918 | 273 | 159 | 90.07 | 6.28 | 3.66 |
| *M. bovis* BCG Korea 1168P | L8 | 3991 | 286 | 9 | 93.12 | 6.67 | 0.21 |
| *M. bovis* BCG Mexico | L8 | 3963 | 296 | 4 | 92.96 | 6.94 | 0.09 |
| *M. bovis* BCG Moreau RDJ | L8 | 3953 | 303 | 7 | 92.73 | 7.11 | 0.16 |
| *M. bovis* BCG Tokyo 172 | L8 | 3973 | 324 | 9 | 92.27 | 7.52 | 0.21 |
| *M. bovis* BCG 3281 | L8 | 4016 | 292 | 17 | 92.86 | 6.75 | 0.39 |
| *M. bovis* BCG Russia 368 | L8 | 3973 | 322 | 9 | 92.31 | 7.48 | 0.21 |
| *M. bovis* BCG 26 | L8 | 3951 | 319 | 78 | 90.87 | 7.34 | 1.79 |
