# Supplementary material for: Pan-Genomic Study of Mycobacterium tuberculosis Reflecting the Primary/Secondary Genes, Generality/Individuality, and the Interconversion Through Copy Number Variations
Source: Front Microbiol. 2018 Aug 17;9:1886. doi: 10.3389/fmicb.2018.01886 (PMC6109687; doi:10.3389/fmicb.2018.01886)
Supplement: Supplementary file 5 [file Table_5.DOCX]

Supplementary Table S5. Core PE/PPE, virulence, and antigen genes found in the Mtb strains.

| **PE/PPE genes** | | | | | | | | | |
| --- | --- | --- | --- | --- | --- | --- | --- | --- | --- |
| **PPE1 (Rv0096)** | **PE_PGRS1 (Rv0109)** | PE_PGRS2 (Rv0124) | **PE1 (Rv0151c)** | **PE2 (Rv0152c)** | **PE3 (Rv0159c)** | **PE4 (Rv0160c)** | **PPE2 (Rv0256c)** | **Rv0278c (Rv0278c)** | PE_PGRS4 (Rv0279c) |
| **PPE3 (Rv0280)** | **PE5 (Rv0285)** | **PPE4 (Rv0286)** | PE_PGRS5 (Rv0297) | **PPE5 (Rv0304c)** | PPE6 (Rv0305c) | **PE6 (Rv0335c)** | PPE7 (Rv0354c) | **PPE8 (Rv0355c)** | **PPE10 (Rv0442c)** |
| **PPE11 (Rv0453)** | PE_PGRS6 (Rv0532) | PE_PGRS7 (Rv0578c) | PE_PGRS9 (Rv0746) | PE_PGRS10 (Rv0747) | **PE_PGRS11 (Rv0754)** | **PPE12 (Rv0755c)** | PE_PGRS12 (Rv0832) | PE_PGRS13 (Rv0833) | PE_PGRS14 (Rv0834c) |
| **PE_PGRS15 (Rv0872c)** | **PPE13 (Rv0878c)** | **PPE14 (Rv0915c)** | **PE7 (Rv0916c)** | PE_PGRS16 (Rv0977) | PE_PGRS17 (Rv0978c) | PE_PGRS18 (Rv0980c) | **PPE15 (Rv1039c)** | PE8 (Rv1040c) | PE_PGRS19 (Rv1067c) |
| PE_PGRS20 (Rv1068c) | PE_PGRS21 (Rv1087) | PE9 (Rv1088) | PE_PGRS22 (Rv1091) | **PPE16 (Rv1135c)** | **PPE17 (Rv1168c)** | **PE12 (Rv1172c)** | **PE13 (Rv1195)** | PPE18 (Rv1196) | **PE14 (Rv1214c)** |
| PE_PGRS23 (Rv1243c) | PE_PGRS24 (Rv1325c) | PPE19 (Rv1361c) | **PE15 (Rv1386)** | **PPE20 (Rv1387)** | PE_PGRS25 (Rv1396c) | **PE16 (Rv1430)** | PE_PGRS26 (Rv1441c) | PE_PGRS27 (Rv1450c) | PE_PGRS28 (Rv1452c) |
| PE_PGRS29 (Rv1468c) | **PPE21 (Rv1548c)** | **PE17 (Rv1646)** | PE_PGRS30 (Rv1651c) | **PPE22 (Rv1705c)** | **PPE23 (Rv1706c)** | PPE24 (Rv1753c) | wag22 (Rv1759c) | wag22 (Rv1759c) | PE_PGRS31 (Rv1768) |
| PPE25 (Rv1787) | PE18 (Rv1788) | PPE26 (Rv1789) | **PPE27 (Rv1790)** | **PE19 (Rv1791)** | **PPE28 (Rv1800)** | PPE29 (Rv1801) | **PPE30 (Rv1802)** | **PE_PGRS32 (Rv1803c)** | **PE20 (Rv1806)** |
| **PPE32 (Rv1808)** | **PPE33 (Rv1809)** | PE_PGRS33 (Rv1818c) | PE_PGRS34 (Rv1840c) | PPE34 (Rv1917c) | **PPE35 (Rv1918c)** | **PE_PGRS35 (Rv1983)** | PE22 (Rv2107) | **PPE36 (Rv2108)** | PPE37 (Rv2123) |
| PE_PGRS37 (Rv2126c) | PE_PGRS38 (Rv2162c) | **PE23 (Rv2328)** | PE_PGRS39 (Rv2340c) | PPE38 (Rv2352c) | PPE39 (Rv2353c) | **PE_PGRS40 (Rv2371)** | PE24 (Rv2408) | **PPE41 (Rv2430c)** | **PE25 (Rv2431c)** |
| PE_PGRS42 (Rv2487c) | PE_PGRS43 (Rv2490c) | **PE26 (Rv2519)** | PE_PGRS44 (Rv2591) | **PPE42 (Rv2608)** | PE_PGRS45 (Rv2615c) | **PE_PGRS46 (Rv2634c)** | PE_PGRS47 (Rv2741) | **PPE43 (Rv2768c)** | **PE27 (Rv2769c)** |
| **PPE44 (Rv2770c)** | PE_PGRS48 (Rv2853) | **PPE45 (Rv2892c)** | **PPE46 (Rv3018c)** | PE29 (Rv3022A) | **lipY (Rv3097c)** | **PPE49 (Rv3125c)** | PPE50 (Rv3135) | **PPE51 (Rv3136)** | **PPE52 (Rv3144c)** |
| PPE53 (Rv3159c) | PPE54 (Rv3343c) | PE_PGRS49 (Rv3344c) | PE_PGRS50 (Rv3345c) | **PPE55 (Rv3347c)** | PPE56 (Rv3350c) | PE_PGRS51 (Rv3367) | PE_PGRS52 (Rv3388) | PPE57 (Rv3425) | PPE58 (Rv3426) |
| PPE59 (Rv3429) | PE31 (Rv3477) | **PPE60 (Rv3478)** | PE_PGRS53 (Rv3507) | PE_PGRS54 (Rv3508) | PE_PGRS55 (Rv3511) | PE_PGRS56 (Rv3512) | PE_PGRS57 (Rv3514) | **PPE61 (Rv3532)** | **PPE62 (Rv3533c)** |
| **PPE63 (Rv3539)** | **PPE64 (Rv3558)** | PE_PGRS58 (Rv3590c) | PE_PGRS59 (Rv3595c) | **PPE65 (Rv3621c)** | **PE32 (Rv3622c)** | PE33 (Rv3650) | PE_PGRS60 (Rv3652) | PE_PGRS61 (Rv3653) | PPE66 (Rv3738c) |
| PPE67 (Rv3739c) | **PE34 (Rv3746c)** | **PE_PGRS62 (Rv3812)** | **PE35 (Rv3872)** | **PPE68 (Rv3873)** | **PPE69 (Rv3892c)** | **PE36 (Rv3893c)** |  |  |  |
| **VF genes** | | | | | | | | | |
| **fmt (CCDC5079_1420)** | pks15/1 (CCDC5079_2706) | **mps1 (Rv0101)** | **fbpC (Rv0129c)** | **mce1A (Rv0169)** | **mce1B (Rv0170)** | **mce1C (Rv0171)** | **mce1D (Rv0172)** | **mce1E (Rv0173)** | **mce1F (Rv0174)** |
| **zmp1 (Rv0198c)** | mmpL11 (Rv0202c) | **Rv0203 (Rv0203)** | **mmpL3 (Rv0206c)** | **fadE5 (Rv0244c)** | **eccA3 (Rv0282)** | **eccB3 (Rv0283)** | **eccC3 (Rv0284)** | **PE5 (Rv0285)** | **PPE4 (Rv0286)** |
| **esxG (Rv0287)** | **esxH (Rv0288)** | **espG3 (Rv0289)** | **eccD3 (Rv0290)** | **mycP3 (Rv0291)** | **eccE3 (Rv0292)** | **stf0 (Rv0295c)** | **rmlA (Rv0334)** | **mosR (Rv0348)** | **pknG (Rv0410c)** |
| **sodC (Rv0432)** | **mmpS4 (Rv0451c)** | **icl (Rv0467)** | pcaA (Rv0470c) | **hbhA (Rv0475)** | **senX3 (Rv0490)** | **regX3 (Rv0491)** | **proC (Rv0500)** | **cmaA2 (Rv0503c)** | **mce2A (Rv0589)** |
| mce2B (Rv0590) | **mce2C (Rv0591)** | **mce2D (Rv0592)** | **mce2E (Rv0593)** | **mce2F (Rv0594)** | **mmaA4 (Rv0642c)** | **sigL (Rv0735)** | **phoP (Rv0757)** | **phoR (Rv0758)** | **purC (Rv0780)** |
| **ompA (Rv0899)** | **prrB (Rv0902c)** | **prrA (Rv0903c)** | ctpV (Rv0969) | **mprA (Rv0981)** | **mprB (Rv0982)** | **narG (Rv1161)** | **narH (Rv1162)** | **narJ (Rv1163)** | **narI (Rv1164)** |
| **papA3 (Rv1182)** | **mmpL10 (Rv1183)** | **pe (Rv1184c)** | **fad23 (Rv1185c)** | **sigE (Rv1221)** | **lpqY (Rv1235)** | **sugA (Rv1236)** | **sugB (Rv1237)** | **sugC (Rv1238)** | lysA (Rv1293) |
| **Rv1344 (Rv1344)** | **fadD33 (Rv1345)** | **fadE14 (Rv1346)** | **mbtK (Rv1347c)** | **irtA (Rv1348)** | **irtB (Rv1349)** | **lprG (Rv1411c)** | gtf2 (Rv1524) | gtf1 (Rv1526c) | PE_PGRS30 (Rv1651c) |
| **narX (Rv1736c)** | **narK2 (Rv1737c)** | plcD (Rv1755c) | **eccB5 (Rv1782)** | **eccCa5 (Rv1783)** | eccCb5 (Rv1784) | **cyp143 (Rv1785c)** | **Rv1786 (Rv1786)** | PPE25 (Rv1787) | PE18 (Rv1788) |
| PPE26 (Rv1789) | **PPE27 (Rv1790)** | **PE19 (Rv1791)** | **esxM (Rv1792*)** | **esxN (Rv1793)** | **Rv1794 (Rv1794)** | **eccD5 (Rv1795)** | **mycP5 (Rv1796)** | **eccE5 (Rv1797)** | **eccA5 (Rv1798)** |
| **mgtC (Rv1811)** | **secA2 (Rv1821)** | **fbpB (Rv1886c)** | **katG (Rv1908c)** | icl2 (Rv1915) | icl2 (Rv1916) | **mce3A (Rv1966)** | **mce3B (Rv1967)** | mce3C (Rv1968) | **mce3D (Rv1969)** |
| **mce3E (Rv1970)** | **mce3F (Rv1971)** | **hspX (Rv2031c)** | **pafA (Rv2097c)** | **mpa (Rv2115c)** | trpD (Rv2192c) | **glnA1 (Rv2220)** | **caeA (Rv2224c)** | **ptpA (Rv2234)** | **kasB (Rv2246)** |
| **plcC (Rv2349c)** | plcB (Rv2350c) | plcA (Rv2351c) | **mbtH (Rv2377c)** | **mbtG (Rv2378c)** | **mbtF (Rv2379c)** | **mbtE (Rv2380c)** | **mbtD (Rv2381c)** | **mbtC (Rv2382c)** | **mbtB (Rv2383c)** |
| **mbtA (Rv2384)** | **mbtJ (Rv2385)** | **mbtI (Rv2386c)** | **eis (Rv2416c)** | **ahpC (Rv2428)** | **PPE41 (Rv2430c)** | **ndk (Rv2445c)** | **relA (Rv2583c)** | **sigA/rpoV (Rv2703)** | **ideR (Rv2711)** |
| **tesA (Rv2928)** | **fadD26 (Rv2930)** | **ppsA (Rv2931)** | **ppsB (Rv2932)** | **ppsC (Rv2933)** | **ppsD (Rv2934)** | **ppsE (Rv2935)** | **ddrA (Rv2936)** | **ddrB (Rv2937)** | **drrC (Rv2938)** |
| **papA5 (Rv2939)** | **mas (Rv2940c)** | **fadD28 (Rv2941)** | **mmpL7 (Rv2942)** | **lppx (Rv2945c)** | **pks1 (Rv2946c)** | pks15 (Rv2947c) | **fadD22 (Rv2948c)** | Rv2949c (Rv2949c) | **fadD29 (Rv2950c)** |
| **Rv2951c (Rv2951c)** | **Rv2952 (Rv2952)** | **Rv2953 (Rv2953)** | **Rv2954c (Rv2954c)** | **Rv2955c (Rv2955c)** | Rv2956 (Rv2956) | **Rv2957 (Rv2957)** | **Rv2958c (Rv2958c)** | **Rv2959c (Rv2959c)** | Rv2962c (Rv2962c) |
| **leuD (Rv2987c)** | **mymA (Rv3083)** | **lipR (Rv3084)** | sadH (Rv3085) | **adhD (Rv3086)** | **chp (Rv3087)** | **tgs4 (Rv3088)** | **fadD13 (Rv3089)** | **devS (Rv3132c)** | **devR/dosR (Rv3133c)** |
| **nuoG (Rv3151)** | **sigH (Rv3223c)** | **kefB (Rv3236c)** | **ctpC (Rv3270)** | **sigF (Rv3286c)** | **sapM (Rv3310)** | **sigD (Rv3414c)** | whiB3 (Rv3416) | **esxT (Rv3444c)** | **esxU (Rv3445c)** |
| **Rv3446c (Rv3446c)** | **eccC4 (Rv3447c)** | cccD4 (Rv3448) | mycP4 (Rv3449) | **eccB4 (Rv3450c)** | **lipF (Rv3487c)** | **mce4F (Rv3494c)** | **mce4E (Rv3495c)** | **mce4D (Rv3496c)** | **mce4C (Rv3497c)** |
| **mce4B (Rv3498c)** | **mce4A (Rv3499c)** | **fadE29 (Rv3543c)** | **fadE28 (Rv3544c)** | **cyp125 (Rv3545c)** | **panD (Rv3601c)** | **panC (Rv3602c)** | **espD (Rv3614c)** | **espC (Rv3615c)** | **espA (Rv3616c)** |
| **lpqH (Rv3763)** | **fbpA (Rv3804c)** | **erp (Rv3810)** | **papA2 (Rv3820c)** | **sap (Rv3821)** | chp1 (Rv3822) | **mmpL8 (Rv3823c)** | **papA1 (Rv3824c)** | pks2 (Rv3825c) | **fad23 (Rv3826)** |
| **sodA (Rv3846)** | **espR (Rv3849)** | **espE (Rv3864)** | **espF (Rv3865)** | **espG1 (Rv3866)** | **espH (Rv3867)** | **eccA1 (Rv3868)** | **eccB1 (Rv3869)** | **eccCa1 (Rv3870)** | **eccCb1 (Rv3871)** |
| **PE35 (Rv3872)** | **PPE68 (Rv3873)** | **esxB (Rv3874)** | **esxA (Rv3875)** | espI (Rv3876) | **eccD1 (Rv3877)** | **espJ (Rv3878)** | espK (Rv3879c) | **espL (Rv3880c)** | **espB (Rv3881c)** |
| **eccE1 (Rv3882c)** | **mycP1 (Rv3883c)** | **eccA2 (Rv3884c)** | **eccE2 (Rv3885c)** | **mycP2 (Rv3886c)** | **eccD2 (Rv3887c)** | **Rv3888c (Rv3888c)** | **espG2 (Rv3889c)** | **esxC (Rv3890c)** | **esxD (Rv3891c)** |
| **PPE69 (Rv3892c)** | **PE36 (Rv3893c)** | eccC2 (Rv3894c) | **eccB2 (Rv3895c)** | **sigM (Rv3911)** |  |  |  |  |  |
| **Antigens** | | | | | | | | | |
| moaA3 (BQ2027_MB3355C) | **pknA (Rv0015c)** | **fadD34 (Rv0035)** | **rplI (Rv0056)** | **icd2 (Rv0066c)** | **Rv0068 (Rv0068)** | **Rv0110 (Rv0110)** | **pepA (Rv0125)** | **fbpC (Rv0129c)** | **Rv0142 (Rv0142)** |
| Rv0145 (Rv0145) | **Rv0146 (Rv0146)** | **PE3 (Rv0159c)** | **TB18.5 (Rv0164)** | **mce1A (Rv0169)** | **mce1B (Rv0170)** | **mce1C (Rv0171)** | **mce1D (Rv0172)** | **lprK (Rv0173)** | **mce1F (Rv0174)** |
| **Rv0191 (Rv0191)** | Rv0192A (Rv0192A) | **zmp1 (Rv0198c)** | **Rv0203 (Rv0203)** | **mmpL3 (Rv0206c)** | **lipC (Rv0220)** | **Rv0221 (Rv0221)** | **echA1 (Rv0222)** | **fadE5 (Rv0244c)** | **Rv0249c (Rv0249c)** |
| **hsp (Rv0251c)** | **PPE2 (Rv0256c)** | PE_PGRS3 (Rv0278c) | **PPE3 (Rv0280)** | **eccC3 (Rv0284)** | **PPE4 (Rv0286)** | **esxG (Rv0287)** | **esxH (Rv0288)** | **espG3 (Rv0289)** | **eccD3 (Rv0290)** |
| **mycP3 (Rv0291)** | **eccE3 (Rv0292)** | **Rv0293c (Rv0293c)** | **tam (Rv0294)** | **Rv0295c (Rv0295c)** | PE_PGRS5 (Rv0297) | **Rv0298 (Rv0298)** | **Rv0299 (Rv0299)** | **Rv0309 (Rv0309)** | **aspC (Rv0337c)** |
| **Rv0339c (Rv0339c)** | **iniB (Rv0341)** | **dnaK (Rv0350)** | **Rv0371c (Rv0371c)** | **Rv0372c (Rv0372c)** | **purT (Rv0389)** | **lpqL (Rv0418)** | **groEL2 (Rv0440)** | hsp65 (Rv0440) | **PPE10 (Rv0442c)** |
| **ufaA1 (Rv0447c)** | **PPE11 (Rv0453)** | **Rv0476 (Rv0476)** | **Rv0508 (Rv0508)** | **gabP (Rv0522)** | **ccsA (Rv0529)** | **Rv0538 (Rv0538)** | **Rv0544c (Rv0544c)** | **fadD8 (Rv0551c)** | **mce2A (Rv0589)** |
| **lprL (Rv0593)** | **hadC (Rv0637)** | **rplK (Rv0640)** | **rplL (Rv0652)** | **rpoB (Rv0667)** | rpoC (Rv0668) | **end (Rv0670)** | **Rv0690c (Rv0690c)** | **Rv0691c (Rv0691c)** | **pqqE (Rv0693)** |
| **lldD1 (Rv0694)** | **rplW (Rv0703)** | PE_PGRS10 (Rv0747) | **PPE12 (Rv0755c)** | **ggtA (Rv0773c)** | **Rv0776c (Rv0776c)** | **purB (Rv0777)** | **Rv0787 (Rv0787)** | PE_PGRS14 (Rv0834c) | pdc (Rv0853c) |
| **Rv0854 (Rv0854)** | **Rv0890c (Rv0890c)** | Rv0894 (Rv0894) | **PPE14 (Rv0915c)** | **PE7 (Rv0916c)** | **pstS3 (Rv0928)** | **pknD (Rv0931c)** | **pstS2 (Rv0932c)** | **pstS1 (Rv0934)** | **purN (Rv0956)** |
| **Rv0959 (Rv0959)** | **mscL (Rv0985c)** | **Rv0987 (Rv0987)** | **Rv0988 (Rv0988)** | **galU (Rv0993)** | **rpfB (Rv1009)** | **glmU (Rv1018c)** | **kdpE (Rv1027c)** | **kdpD (Rv1028c)** | **Rv1036c (Rv1036c)** |
| **esxJ (Rv1038c)** | PPE15 (Rv1039c) | **PE8 (Rv1040c)** | **Rv1045 (Rv1045)** | PE9 (Rv1088) | PE_PGRS22 (Rv1091) | **vapC32 (Rv1114)** | **Rv1128c (Rv1128c)** | Rv1157c (Rv1157c) | Rv1158c (Rv1158c) |
| **PE12 (Rv1172c)** | **TB8.4 (Rv1174c)** | **Rv1184c (Rv1184c)** | **fadD21 (Rv1185c)** | **Rv1186c (Rv1186c)** | **rocA (Rv1187)** | **Rv1188 (Rv1188)** | **PE13 (Rv1195)** | PPE18 (Rv1196) | esxK (Rv1197) |
| esxL (Rv1198) | **tagA (Rv1210)** | vapC33 (Rv1242) | PE_PGRS23 (Rv1243c) | **deaD (Rv1253)** | **Rv1255c (Rv1255c)** | **cyp130 (Rv1256c)** | **lprA (Rv1270c)** | **oppA (Rv1280c)** | **cysN (Rv1286)** |
| Rv1291c (Rv1291c) | **rho (Rv1297)** | **hemK (Rv1300)** | **Rv1301 (Rv1301)** | **atpH (Rv1307)** | **atpA (Rv1308)** | **atpD (Rv1310)** | **atpC (Rv1311)** | **Rv1312 (Rv1312)** | **ogt (Rv1316c)** |
| **alkA (Rv1317c)** | mec (Rv1334) | **Rv1339 (Rv1339)** | **irtA (Rv1348)** | PPE19 (Rv1361c) | **Rv1366 (Rv1366)** | **Rv1367c (Rv1367c)** | **Rv1382 (Rv1382)** | **PE15 (Rv1386)** | **PPE20 (Rv1387)** |
| **Rv1405c (Rv1405c)** | **Rv1410c (Rv1410c)** | **uvrC (Rv1420)** | **Rv1431 (Rv1431)** | Rv1434 (Rv1434) | **gap (Rv1436)** | PE_PGRS26 (Rv1441c) | PE_PGRS27 (Rv1450c) | **Rv1453 (Rv1453)** | **Rv1461 (Rv1461)** |
| **inhA (Rv1484)** | **Rv1490 (Rv1490)** | **mutB (Rv1493)** | Rv1503c (Rv1503c) | **Rv1513 (Rv1513)** | **Rv1518 (Rv1518)** | **Rv1523 (Rv1523)** | **Rv1535 (Rv1535)** | **Rv1565c (Rv1565c)** | **bioA (Rv1568)** |
| Rv1582c (Rv1582c) | **lgt (Rv1614)** | **pykA (Rv1617)** | **cydA (Rv1623c)** | **TB15.3 (Rv1636)** | **Rv1639c (Rv1639c)** | **infC (Rv1641)** | **rpmI (Rv1642)** | **Rv1648 (Rv1648)** | **lprJ (Rv1690)** |
| **tlyA (Rv1694)** | **PPE22 (Rv1705c)** | **Rv1733c (Rv1733c)** | **Rv1734c (Rv1734c)** | **narK2 (Rv1737c)** | mtp40 (Rv1755c) | **Rv1769 (Rv1769)** | **cyp143 (Rv1785c)** | PPE25 (Rv1787) | PE18 (Rv1788) |
| PPE26 (Rv1789) | **PE19 (Rv1791)** | **esxN (Rv1793)** | **PPE28 (Rv1800)** | **PPE30 (Rv1802)** | **PE20 (Rv1806)** | **PPE32 (Rv1808)** | **Rv1813c (Rv1813c)** | PE_PGRS33 (Rv1818c) | **garA (Rv1827)** |
| **glcB (Rv1837c)** | **gnd1 (Rv1844c)** | **apa (Rv1860)** | **Rv1866 (Rv1866)** | **Rv1870c (Rv1870c)** | Rv1871c (Rv1871c) | **lldD2 (Rv1872c)** | **Rv1873 (Rv1873)** | **bfrA (Rv1876)** | **Rv1877 (Rv1877)** |
| **Rv1879 (Rv1879)** | **Rv1885c (Rv1885c)** | **fbpB (Rv1886c)** | fbpB (Rv1886c) | KatG (Rv1908c) | fadB5 (Rv1912c) | PPE34 (Rv1917c) | **mpt63 (Rv1926c)** | **Rv1945 (Rv1945)** | **yrbE3B (Rv1965)** |
| **Rv1973 (Rv1973)** | **Rv1977 (Rv1977)** | **Rv1979c (Rv1979c)** | **mpt64 (Rv1980c)** | **PE_PGRS35 (Rv1983)** | **cfp21 (Rv1984c)** | **Rv1985c (Rv1985c)** | **Rv1986 (Rv1986)** | Rv1987 (Rv1987) | ctpF (Rv1997) |
| **Rv2004c (Rv2004c)** | **otsB1 (Rv2006)** | **Rv2015c (Rv2015c)** | **pfkB (Rv2029c)** | **Rv2030c (Rv2030c)** | **hspX (Rv2031c)** | **Rv2034 (Rv2034)** | **sigC (Rv2069)** | **Rv2074 (Rv2074)** | **tatA (Rv2094c)** |
| **pafB (Rv2096c)** | **Rv2119 (Rv2119)** | **hisE (Rv2122c)** | PPE37 (Rv2123) | **Rv2182c (Rv2182c)** | **TB16.3 (Rv2185c)** | **Rv2190c (Rv2190c)** | **Rv2191 (Rv2191)** | **ctaC (Rv2200c)** | **dlaT (Rv2215)** |
| **glnA1 (Rv2220)** | **Rv2223c (Rv2223c)** | **Rv2226 (Rv2226)** | **kasB (Rv2246)** | Rv2273 (Rv2273) | lppO (Rv2290) | esxO (Rv2346c) | plcB (Rv2350c) | **cfp2 (Rv2376c)** | **PPE41 (Rv2430c)** |
| **Rv2433c (Rv2433c)** | **mobA (Rv2453c)** | clpP2 (Rv2460c) | **gdh (Rv2476c)** | PE_PGRS42 (Rv2487c) | PE_PGRS43 (Rv2490c) | **bkdC (Rv2495c)** | **Rv2512c (Rv2512c)** | vapB17 (Rv2526) | **Rv2531c (Rv2531c)** |
| **Rv2567 (Rv2567)** | **aspS (Rv2572c)** | **Rv2574 (Rv2574)** | **Rv2575 (Rv2575)** | Rv2576c (Rv2576c) | **Rv2600 (Rv2600)** | **PPE42 (Rv2608)** | **Rv2609c (Rv2609c)** | **hrp1 (Rv2626c)** | **Rv2627c (Rv2627c)** |
| **Rv2628 (Rv2628)** | **Rv2629 (Rv2629)** | **PE_PGRS46 (Rv2634c)** | Rv2653c (Rv2653c) | **Rv2654c (Rv2654c)** | **Rv2658c (Rv2658c)** | **Rv2666 (Rv2666)** | **arsA (Rv2684)** | **Rv2716 (Rv2716)** | **recX (Rv2736c)** |
| **PPE44 (Rv2770c)** | **ald (Rv2780)** | Rv2819c (Rv2819c) | **Rv2823c (Rv2823c)** | PE_PGRS48 (Rv2853) | **mtr (Rv2855)** | **mapB (Rv2861c)** | **relF (Rv2865)** | **Rv2867c (Rv2867c)** | **gcpE (Rv2868c)** |
| **rip (Rv2869c)** | **mpt83 (Rv2873)** | **dipZ (Rv2874)** | **mpt70 (Rv2875)** | **mpt53 (Rv2878c)** | **cdsA (Rv2881c)** | **lepB (Rv2903c)** | **ppsB (Rv2932)** | **mas (Rv2940c)** | FadD28 (Rv2941) |
| FadD28_1 (Rv2941) | **lppX (Rv2945c)** | **Rv2954c (Rv2954c)** | **Rv2955c (Rv2955c)** | **Rv2957 (Rv2957)** | **Rv2958c (Rv2958c)** | Rv2962c (Rv2962c) | **Rv2963 (Rv2963)** | **ppk1 (Rv2984)** | hupB (Rv2986c) |
| **leuD (Rv2987c)** | **leuC (Rv2988c)** | Rv2994 (Rv2994) | **serA1 (Rv2996c)** | **Rv2997 (Rv2997)** | **lppY (Rv2999)** | **Rv3000 (Rv3000)** | **ilvC (Rv3001c)** | **ilvN (Rv3002c)** | **ilvB1 (Rv3003c)** |
| **cfp6 (Rv3004)** | **lppZ (Rv3006)** | Rv3007c (Rv3007c) | **pfkA (Rv3010c)** | **gatC (Rv3012c)** | **ligA (Rv3014c)** | **Rv3015c (Rv3015c)** | **esxQ (Rv3017c)** | PPE46 (Rv3018c) | PPE46 (Rv3018c) |
| esxR (Rv3019c) | esxS (Rv3020c) | PPE47/PPE48 (Rv3021c/Rv3022c) | **trmU (Rv3024c)** | **iscS (Rv3025c)** | **Rv3026c (Rv3026c)** | **fixB (Rv3028c)** | **fixA (Rv3029c)** | **Rv3031 (Rv3031)** | **Rv3032 (Rv3032)** |
| **Rv3035 (Rv3035)** | **fadE22 (Rv3061c)** | **ligB (Rv3062)** | **ftsX (Rv3101c)** | **fprA (Rv3106)** | **moaA1 (Rv3109)** | Rv3115 (Rv3115) | **PPE49 (Rv3125c)** | **devR (Rv3133c)** | PPE50 (Rv3135) |
| **PPE51 (Rv3136)** | **Rv3142c (Rv3142c)** | **Rv3143 (Rv3143)** | **PPE52 (Rv3144c)** | **nuoB (Rv3146)** | **nuoD (Rv3148)** | **Rv3161c (Rv3161c)** | **Rv3173c (Rv3173c)** | Rv3189 (Rv3189) | **Rv3201c (Rv3201c)** |
| **Rv3207c (Rv3207c)** | **whiB1 (Rv3219)** | **TB7.3 (Rv3221c)** | **ppk2 (Rv3232c)** | **tmk (Rv3247c)** | **Rv3267 (Rv3267)** | **Rv3268 (Rv3268)** | **Rv3282 (Rv3282)** | **accA3 (Rv3285)** | **lhr (Rv3296)** |
| **lpqC (Rv3298c)** | **Rv3300c (Rv3300c)** | **dacB1 (Rv3330)** | **Rv3333c (Rv3333c)** | **icd1 (Rv3339c)** | PPE54 (Rv3343c) | **PPE55 (Rv3347c)** | **Rv3354 (Rv3354)** | **dnaE2 (Rv3370c)** | Rv3378c (Rv3378c) |
| **cmaA1 (Rv3392c)** | **iunH (Rv3393)** | **vapB47 (Rv3407)** | **groES (Rv3418c)** | **gcp (Rv3419c)** | PPE57 (Rv3425) | Rv3427c (Rv3427c) | Rv3428c (Rv3428c) | PPE59 (Rv3429) | **Rv3431c (Rv3431c)** |
| **Rv3467 (Rv3467)** | PE31 (Rv3477) | **PPE60 (Rv3478)** | **mce4C (Rv3497c)** | **mce4B (Rv3498c)** | **mce4A (Rv3499c)** | **yrbE4B (Rv3500c)** | PE_PGRS53 (Rv3507) | **PPE61 (Rv3532)** | **lysS (Rv3598c)** |
| **espC (Rv3615c)** | **espA (Rv3616c)** | ephA (Rv3617) | **esxV (Rv3619c)** | **PPE65 (Rv3621c)** | **galE1 (Rv3634c)** | Rv3649 (Rv3649) | Rv3658c (Rv3658c) | **Rv3689 (Rv3689)** | Rv3693 (Rv3693) |
| **Rv3714c (Rv3714c)** | Rv3729 (Rv3729) | **Rv3736 (Rv3736)** | **Rv3737 (Rv3737)** | **lpqH (Rv3763)** | **lipE (Rv3775)** | **Rv3798 (Rv3798)** | **fbpD (Rv3803c)** | **fbpA (Rv3804c)** | **PE_PGRS62 (Rv3812)** |
| **mmpL8 (Rv3823c)** | **papA1 (Rv3824c)** | pks2 (Rv3825c) | **serS (Rv3834c)** | **Rv3839 (Rv3839)** | **bfrB (Rv3841)** | **sodA (Rv3846)** | **Rv3847 (Rv3847)** | **Rv3856c (Rv3856c)** | **gltB (Rv3859c)** |
| **espE (Rv3864)** | **espF (Rv3865)** | **eccCb1 (Rv3871)** | **PPE68 (Rv3873)** | PPE68 (Rv3873) | **esxB (Rv3874)** | **esxA (Rv3875)** | esxA (Rv3875) | espI (Rv3876) | **espJ (Rv3878)** |
| espK (Rv3879c) | **espB (Rv3881c)** | **mycP1 (Rv3883c)** | **trxC (Rv3914)** | Rv3922c (Rv3922c) |  |  |  |  |  |

*Core PE/PPE proteins, VFs, and antigens were shown in bold.
