# Supplementary material for: Pan-Genomic Study of Mycobacterium tuberculosis Reflecting the Primary/Secondary Genes, Generality/Individuality, and the Interconversion Through Copy Number Variations
Source: Front Microbiol. 2018 Aug 17;9:1886. doi: 10.3389/fmicb.2018.01886 (PMC6109687; doi:10.3389/fmicb.2018.01886)
Supplement: Supplementary file 6 [file Table_6.DOCX]

**Supplementary Table S6.** Functional and phenotypic analysis of core PE/PPE, VFs, and antigens in the Mtb strains.

| **Phenotype** | **Sub-phenotype** | **Gene** | **Synonym** | **COG** | **Annotation** | **Function/Mechanism** | **Reference** |
| --- | --- | --- | --- | --- | --- | --- | --- |
| Complex cell envelope | Cell wall integrity and cell morphtype | *pknA* | *Rv0015c* | COG0515RTKL | serine/threonine-protein kinase PknA | PknA involves in cell wall synthesis and plays important role in determining cell shape, morphology, and cell division,a and mediate the signaling that slows down the in vitro growth of *M. tuberculosis* | (Forrellad et al., 2013) |
| Complex cell envelope | Cell wall integrity and cell morphtype | *fbpC* | *Rv0129c* | COG0627R | diacylglycerol acyltransferase/mycolyltransferase Ag85C | FbpC have mycolyltransferase activity, required for maintaining the integrity of the mycobacterial cell envelope | (Forrellad et al., 2013) |
| Complex cell envelope |  | *TB18.5* | *Rv0164* | COG2867I | hypothetical protein | TB18.5 probably play an irreplaceable role during mycobacterial growth, for example in lipid transfer during cell envelope synthesis | (Zheng et al., 2018) |
| Complex cell envelope | Cell wall integrity and cell morphtype | *pknG* | *Rv0410c* | COG0515RTKL | serine/threonine-protein kinase PknG | *pknG* plays potential role in central carbon metabolism and in cell wall biosynthesis | (Forrellad et al., 2013) |
| Complex cell envelope | Cell wall integrity and cell morphtype | *hadC* | *Rv0637* | COG2030I | (3R)-hydroxyacyl-ACP dehydratase subunit HadC | The mutation or deletion of *hadC* affects the biosynthesis of oxygenated mycolic acids, and has an impact on the morphotype, cording capacity and biofilm growth of the bacilli as well as on their sensitivity to agents such as rifampicin. The deletion of hadC leads to a dramatic loss of virulence. | (Slama et al., 2016) |
| Complex cell envelope |  | *mmaA4* | *Rv0642c* | COG2230M | hydroxymycolate synthase MmaA4 | *M. tuberculosis* mutant with an inactivated *mmaA4* gene displayed a profound alteration in its envelope permeability as well as the loss of oxygenated mycolic acids. | (Forrellad et al., 2013) |
| Complex cell envelope | Host-cell entry | *sigL* | *Rv0735* | COG1595K | ECF RNA polymerase sigma factor SigL | SigL regulates the expression of proteins involved in lipid metabolism and cell envelope, such as polyketide synthase, lipid transporters, enzymes of lipid biogenesis, etc., among other mycobacterial genes. The lack of *sigL* in *M. tuberculosis* results in an immunopathology defect of virulence in mice as it has been reported for mutants in many other sigma factors | (Forrellad et al., 2013) |
| Complex cell envelope | Hyper virulent | *Rv0987* | *Rv0987* | COG4591M | adhesion component ABC transporter permease | Rv0987 disruption might alter the architecture and integrity of the mycobacterial cell wall, which is likely to affect the adherence properties of the bacillus | (Rosas-Magallanes et al., 2007) |
| Complex cell envelope |  | *galU* | *Rv0993* | COG1210M | UTP--glucose-1-phosphate uridylyltransferase | *galU* is involved in cell envelope precursor synthesis | (Lai et al., 2008) |
| Complex cell envelope | Host-cell entry | *Rv1184c* | *Rv1184c* | - | hypothetical protein | Rv1184c is essential for the final steps of polyacyltrehalose (a virulence-associated outer membrane lipids) biosynthesis. | (Touchette et al., 2015) |
| Complex cell envelope |  | *fadD21* | *Rv1185c* | COG0318IQ | fatty-acid--CoA ligase FadD21 | FadD21 belongs to a family of fatty acyl AMP ligases whose role is to activate long-chain fatty acids as acyl adenylates, which are then transferred to polyketide synthases for further chain extension | (Belardinelli et al., 2014) |
| Complex cell envelope |  | *oppA* | *Rv1280c* | COG0747E | oligopeptide ABC transporter substrate-binding lipoprotein OppA | Belong to *opp* operon, which encoding permeases involved in the uptake of small peptides are annotated in the *M. tuberculosis* H37Rv.Opp may be involved in the import of peptides or lipopeptides to signal the need to adequate/remodel cell wall envelope components. | (Forrellad et al., 2013) |
| Complex cell envelope | Cell wall integrity and cell morphtype | *Rv1410c* | *Rv1410c* | COG2814G | aminoglycosides/tetracycline-transport integral membrane protein | Rv1410c plays a role in maintaining general metabolism.It is a subunit of the Mce4 transport apparatus (catalyzing its assembly or providing cell wall integrity) that allow more efficient cholesterol uptake. | (Ramon-Garcia et al., 2015) |
| Complex cell envelope | Host-cell entry | *inhA* | *Rv1484* | COG0623I | NADH-dependent enoyl-(Kawaguchi et al.) reductase | InhA is a key enzyme of the fatty-acid synthase II system involved in mycolic acid biosynthesis. | (Forrellad et al., 2013) |
| Complex cell envelope | Host-cell entry | *lgt* | *Rv1614* | COG0682M | prolipoprotein diacylglyceryl transferase | Lgt is the gating enzyme of lipoprotein biosynthesis,which is often involved in virulence and immunoregulatory processes. Lgt is essential in *M. tuberculosis.* | (Tschumi et al., 2012) |
| Complex cell envelope | Cell wall integrity and cell morphtype | *cydA* | *Rv1623c* | COG1271C | cytochrome D ubiquinol oxidase subunit I CydA | Cytochrome bd is composed of two integral membrane polypeptides, subunits I (CydA, 57 kDa) and II (CydB, 43 kDa), and a small protein, named CydX (4 kDa), that has been recently found to be important for the enzymatic activity and proposed to be an additional subunit of the complex | (Giuffre et al., 2014) |
| Complex cell envelope | Host-cell entry | *rip* | *Rv2869c* | COG0750M | zinc metalloprotease | Rv2869c protease participates in multiple lipid biosynthetic pathways possible through cleavage of membrane bound transcriptional regulators. It is related to virulence. | (Forrellad et al., 2013) |
| Complex cell envelope | Hyper virulent | *ppsA* | *Rv2931* | COG3321Q | phthiocerol synthesis polyketide synthase type I PpsA | PpsA-ppsE operon (Rv2931 to 2935) encoding a polyketide synthase required for phthiocerol biosythesis, effect the strain deficient in PDIM production. | (Forrellad et al., 2013) |
| Complex cell envelope | Hyper virulent | *ppsB* | *Rv2932* | COG3321Q | phthiocerol synthesis polyketide synthase type I PpsB | PpsA-ppsE operon (Rv2931 to 2935) encoding a polyketide synthase required for phthiocerol biosythesis, effect the strain deficient in PDIM production. | (Forrellad et al., 2013) |
| Complex cell envelope | Hyper virulent | *ppsC* | *Rv2933* | COG3321Q | phthiocerol synthesis polyketide synthase type I PpsC | PpsA-ppsE operon (Rv2931 to 2935) encoding a polyketide synthase required for phthiocerol biosythesis, effect the strain deficient in PDIM production. | (Forrellad et al., 2013) |
| Complex cell envelope | Hyper virulent | *ppsD* | *Rv2934* | COG3321Q | phthiocerol synthesis polyketide synthase type I PpsD | PpsA-ppsE operon (Rv2931 to 2935) encoding a polyketide synthase required for phthiocerol biosythesis, effect the strain deficient in PDIM production. | (Forrellad et al., 2013) |
| Complex cell envelope | Hyper virulent | *ppsE* | *Rv2935* | COG3321Q | phthiocerol synthesis polyketide synthase type I PpsE | PpsA-ppsE operon (Rv2931 to 2935) encoding a polyketide synthase required for phthiocerol biosythesis, effect the strain deficient in PDIM production. | (Forrellad et al., 2013) |
| Complex cell envelope | Hyper virulent | *drrC* | *Rv2938* | COG0842V | daunorubicin ABC transporter permease DrrC | DrrC is a component of the secretion of PDIM, which is a member of an ATP-binding cassette (ABC) transporter and works along with MmpL7; thus defects in any of them lead to PDIM accumulation. | (Forrellad et al., 2013) |
| Complex cell envelope | Cell wall integrity and cell morphtype | *papA5* | *Rv2939* | - | phthiocerol/phthiodiolone dimycocerosyl transferase | PapA5 may be proximal to the membrane in Mtb, consistent with other soluble biosynthetic enzymes that act on lipid intermediates in cell wall biosynthesis and have been localized to polar sites of cell wall growth. | (Touchette et al., 2015) |
| Complex cell envelope | Hyper virulent | *fadD28* | *Rv2941* | COG0318IQ | long-chain-fatty-acid--AMP ligase FadD28 | FadD28 involves in the production of the pathogenesis associated PDIM lipid family .FadD28 is very likely required for mycobacteria virulence. | (Forrellad et al., 2013) |
| Complex cell envelope | Hyper virulent | *mmpL7* | *Rv2942* | COG2409R | transmembrane transport protein MmpL7 | MmpL7 is required for the translocation of PDIMs across the plasma membrane. | (Forrellad et al., 2013) |
| Complex cell envelope | Hyper virulent | *lppX* | *Rv2945c* | - | lipoprotein LppX | The lipoprotein LppX is also a protein required for the translocation of PDIMs to the outer membrane of *M. tuberculosis*. A mutant in the gene encoding this protein was identified as being highly attenuated in the STM experiment. | (Forrellad et al., 2013) |
| Complex cell envelope | Host-cell entry/hyper virulent | *pks1* | *Rv2946c* | COG3321Q | polyketide synthase | An *M. tuberculosis* mutant in the *pks1* gene was deficient in the production of phenolic glycolipids (PGLs), which is associated to the hyper virulent phenotype of *M. tuberculosis* isolates belonging to the W-Beijing family. | (Forrellad et al., 2013) |
| Complex cell envelope | Host-cell entry/hyper virulent | *fadD22* | *Rv2948c* | COG0318IQ | p-hydroxybenzoyl--AMP ligase | FadD22 and FadD29 are responsible for one particular reaction in the PGL biosynthetic pathway. PGL is associated to the hyper virulent phenotype of *M. tuberculosis* isolates belonging to the W-Beijing family. | (Simeone et al., 2010) |
| Complex cell envelope | Host-cell entry/hyper virulent | *fadD29* | *Rv2950c* | - | long-chain-fatty-acid--AMP ligase FadD29 | FadD22 and FadD29 are responsible for one particular reaction in the PGL biosynthetic pathway. PGL is associated to the hyper virulent phenotype of *M. tuberculosis* isolates belonging to the W-Beijing family. | (Simeone et al., 2010) |
| Complex cell envelope | Host-cell entry | *Rv2951c* | *Rv2951c* | COG2141C | phthiodiolone/phenolphthiodiolone dimycocerosates ketoreductase | In *Mycobacterium tuberculosis*, Rv2951c encodes the phthiodiolone ketoreductase (PKR). Disruption of Rv2951c eliminates the production of DIM A and PGL-tb and causes the accumulation of DIM B and glycosylated phenolphthiodiolone dimycocerosates . | (Purwantini et al., 2016) |
| Complex cell envelope | Host-cell entry | *Rv2954c* | *Rv2954c* | COG0030J | hypothetical protein | Rv2954c is involved in the biosynthesis of the glycosyl moiety of PGL and pHBADs). | (Angala et al., 2014) |
| Complex cell envelope | Host-cell entry | *Rv2955c* | *Rv2955c* | COG4123R | hypothetical protein | Rv2955c is involved in the biosynthesis of the glycosyl moiety of PGL and pHBADs). | (Angala et al., 2014) |
| Complex cell envelope | Host-cell entry | *Rv2957* | *Rv2957* | COG0463M | PGL/p-HBAD biosynthesis glycosyltransferase | Rv2957 is involved in the biosynthesis of the glycosyl moiety of PGL and pHBADs). | (Angala et al., 2014) |
| Complex cell envelope | Host-cell entry | *Rv2958c* | *Rv2958c* | COG1819GC | PGL/p-HBAD biosynthesis glycosyltransferase | Rv2958c is involved in the biosynthesis of the glycosyl moiety of PGL and pHBADs). | (Angala et al., 2014) |
| Complex cell envelope | Host-cell entry | *Rv2959c* | *Rv2959c* | COG3510V | rhamnosyl O-methyltransferase | Rv2959c is involved in the biosynthesis of the glycosyl moiety of PGL and pHBADs). | (Angala et al., 2014) |
| Complex cell envelope |  | *regX3* | *Rv0491* | COG0745TK | two component sensory transduction protein RegX | RegX3 both positively and negatively regulates a large and functionally diverse regulon comprised of 100 genes, including those involved in important physiological activities, including energy metabolism, cell envelope maintenance, and regulatory functions. | (Forrellad et al., 2013) |
| Complex cell envelope | Hyper virulent | *fadD26* | *Rv2930* | COG0318IQ | fatty-acid--CoA ligase FadD26 | FadD26 is a fatty-acid-CoA synthase involved in the biosynthesis of pthiocerol dimycocerosic acids (PDIMs). PDIMs constitute major virulence factors of *M. tuberculosis*, in particular during the early step of infection when bacilli encounter their host macrophages. | (Forrellad et al., 2013) |
| Complex cell envelope/Immune responsse | Affecting host cytokine production | *lprA* | *Rv1270c* | - | lipoprotein LprA | LprA is Mtb lipoprotein with Toll-like receptor 2 activity that modulate antigen presenting cell (APC) functions, which we define to include cytokine production and other innate immune responses of APCs as well as antigen presentation, recapitulating many effects of APC infection with Mtb. | (Drage et al., 2009) |
| Complex cell envelope/Immune responsse | protective immunity | *mpt83* | *Rv2873* | COG2335M | cell surface lipoprotein | Mycobacterial MPT83 is a dominant antigen during infection, and immunization of mice with plasmid DNA expressing this antigen induced significant immune responses and protection against challenge with *M. bovis*. | (Xue et al., 2004) |
| Complex cell envelope/Immune responsse |  | *lpqH* | *Rv3763* | - | lipoprotein LpqH | LpqH can inhibit MHC-II antigen processing and presentation in macrophages.It also induces dendritic cell (DC) maturation. | (Forrellad et al., 2013) |
| Complex cell envelope/Intracellular survival | Host-cell entry | *lprK* | *Rv0173* | COG1463Q | Mce family lipoprotein LprK | Belong to *mce*1 operon, which conferring mycobacteria the ability to enter into mammalian cells and survive inside the macrophage. | (Forrellad et al., 2013) |
| Complex cell envelope/Intracellular survival | Host-cell entry | *lprL* | *Rv0593* | COG1463Q | Mce family lipoprotein LprL | Belong to mce2 operon, which have evolved to fulfil specific roles, most likely related to lipid metabolism, modulating pathogenicity through changes in *M. tuberculosis* lipid pathways. | (Forrellad et al., 2013) |
| Complex cell envelope/Intracellular survival | Nutrient absorption | *pstS3* | *Rv0928* | COG0226P | phosphate ABC transporter substrate-binding lipoprotein PstS | PstS-3 is required for phosphate uptake and survival within macrophages. | (Forrellad et al., 2013) |
| Complex cell envelope/Intracellular survival | Nutrient absorption | *pstS2* | *Rv0932c* | COG0226P | phosphate ABC transporter substrate-binding lipoprotein PstS | PstS-2 is involved in phosphate import during starvation, a condition prevailing inside the phagosome. | (Forrellad et al., 2013) |
| Complex cell envelope/Intracellular survival | Nutrient absorption | *pstS1* | *Rv0934* | COG0226P | phosphate ABC transporter substrate-binding lipoprotein PstS | PstS-1 is involved in phosphate import during starvation. | (Forrellad et al., 2013) |
| Complex cell envelope/Intracellular survival |  | *lpqY* | *Rv1235* | COG1653G | trehalose ABC transporter substrate-binding lipoprotein LpqY | lpqY Is a gene required for growth in mice and in macrophages. | (Forrellad et al., 2013) |
| Complex cell envelope/Intracellular survival |  | *dlaT* | *Rv2215* | COG0508C | pyruvate dehydrogenase E2 component dihydrolipoamide acyltransferase | Deletion of *dlaT* had a pronounced effect on Mtb’s growth in standard medium in vitro, sensitized Mtb to RNI, and attenuated Mtb in the mouse. | (Venugopal et al., 2011) |
| Complex cell envelope/Intracellular survival | Hyper virulent | *eccB1* | *Rv3869* | - | ESX-1 secretion system protein EccB | ESX systems function as specialized secretion systems that enable the transport of selected substrates across the complex, thick mycobacterial cell envelope that forms a structural barrier to protein export. ESX-1 secretion system are found to exhibit a range of diverse phenotypes, including defects in immune modulation, tissue invasion, phagosomal trafficking and growth inside the macrophages. | (Sreejit et al., 2014, Groschel et al., 2016) |
| Complex cell envelope/Intracellular survival | Hyper virulent/interferantimicrobial activity of the phagosome | *eccCa1* | *Rv3870* | COG1674D | ESX-1 secretion system protein EccCa | ESX systems function as specialized secretion systems that enable the transport of selected substrates across the complex, thick mycobacterial cell envelope that forms a structural barrier to protein export. ESX-1 secretion system are found to exhibit a range of diverse phenotypes, including defects in immune modulation, tissue invasion, phagosomal trafficking and growth inside the macrophages. | (Sreejit et al., 2014, Groschel et al., 2016) |
| Complex cell envelope/Intracellular survival | Hyper virulent/interferantimicrobial activity of the phagosome | *eccCb1* | *Rv3871* | COG1674D | ESX-1 secretion system protein EccCb | ESX systems function as specialized secretion systems that enable the transport of selected substrates across the complex, thick mycobacterial cell envelope that forms a structural barrier to protein export. ESX-1 secretion system are found to exhibit a range of diverse phenotypes, including defects in immune modulation, tissue invasion, phagosomal trafficking and growth inside the macrophages. | (Sreejit et al., 2014, Groschel et al., 2016) |
| Complex cell envelope/Intracellular survival | Hyper virulent/interferantimicrobial activity of the phagosome | *PE35* | *Rv3872* | - | PE family protein PE35 | ESX systems function as specialized secretion systems that enable the transport of selected substrates across the complex, thick mycobacterial cell envelope that forms a structural barrier to protein export. ESX-1 secretion system are found to exhibit a range of diverse phenotypes, including defects in immune modulation, tissue invasion, phagosomal trafficking and growth inside the macrophages. | (Sreejit et al., 2014, Groschel et al., 2016) |
| Complex cell envelope/Intracellular survival | Hyper virulent/interferantimicrobial activity of the phagosome | *PPE68* | *Rv3873* | COG5651N | PPE family protein PPE68 | ESX systems function as specialized secretion systems that enable the transport of selected substrates across the complex, thick mycobacterial cell envelope that forms a structural barrier to protein export. ESX-1 secretion system are found to exhibit a range of diverse phenotypes, including defects in immune modulation, tissue invasion, phagosomal trafficking and growth inside the macrophages. | (Sreejit et al., 2014, Groschel et al., 2016) |
| Complex cell envelope/Intracellular survival |  | *eccA3* | *Rv0282* | COG0464O | ESX-3 secretion system protein EccA | *eccA3* belongs to ESX-3 secretion system, which plays both irondependent and -independent roles in Mtb pathogenesis. | (Tufariello et al., 2016) |
| Complex cell envelope/Intracellular survival |  | *eccB3* | *Rv0283* | - | ESX-3 secretion system protein EccB3 | *eccB3* belongs to ESX-3 secretion system, which plays both irondependent and -independent roles in Mtb pathogenesis. | (Tufariello et al., 2016) |
| Complex cell envelope/Intracellular survival |  | *eccC3* | *Rv0284* | COG1674D | ESX-3 secretion system protein EccC3 | *eccC3* belonsg to ESX-3 secretion system, which plays both irondependent and -independent roles in Mtb pathogenesis. | (Tufariello et al., 2016) |
| Complex cell envelope/Intracellular survival |  | *esxG* | *Rv0287* | - | ESAT-6 like protein EsxG | *esxG* belongs to ESX-3 secretion system, which plays both irondependent and -independent roles in Mtb pathogenesis. | (Tufariello et al., 2016) |
| Complex cell envelope/Intracellular survival |  | *esxH* | *Rv0288* | COG4842S | ESAT-6-like protein EsxH | *esxH* belongs to ESX-3 secretion system, which plays both irondependent and -independent roles in Mtb pathogenesis. | (Tufariello et al., 2016) |
| Complex cell envelope/Intracellular survival |  | *espG3* | *Rv0289* | - | ESX-3 secretion-associated protein EspG3 | *espG3* belong to ESX-3 secretion system, which plays both irondependent and -independent roles in Mtb pathogenesis. | (Tufariello et al., 2016) |
| Complex cell envelope/Intracellular survival |  | *eccD3* | *Rv0290* | - | ESX-3 secretion system protein EccD | eccD3 belongs to ESX-3 secretion system, which plays both irondependent and -independent roles in Mtb pathogenesis. | (Tufariello et al., 2016) |
| Complex cell envelope/Intracellular survival |  | *mycP3* | *Rv0291* | COG1404O | membrane-anchored mycosin MycP | Belongs to ESX-3 secretion system, which plays both irondependent and -independent roles in Mtb pathogenesis. | (Tufariello et al., 2016) |
| Complex cell envelope/Intracellular survival |  | *eccE3* | *Rv0292* | - | ESX-3 secretion system protein EccE | Belongs to ESX-3 secretion system, which plays both irondependent and -independent roles in Mtb pathogenesis. | (Tufariello et al., 2016) |
| Complex cell envelope/Intracellular survival |  | *esxN* | *Rv1793* | - | ESAT-6 like protein EsxN | *esxN* belongs to ESX-5 system, which is involved in secretion of most PE-PPE proteins in *M. tuberculosis* and is important in mycobacterial pathogenesis. | (Forrellad et al., 2013) |
| Complex cell envelope/Intracellular survival |  | *eccD5* | *Rv1795* | - | ESX-5 type VII secretion system protein EccD | *eccD5* belongs to ESX-5 system, which is involved in secretion of most PE-PPE proteins in *M. tuberculosis* and is important in mycobacterial pathogenesis. | (Forrellad et al., 2013) |
| Complex cell envelope/Intracellular survival |  | *mycP5* | *Rv1796* | COG1404O | membrane-anchored mycosin MycP | *mycP5* belongs to ESX-5 system, which is involved in secretion of most PE-PPE proteins in *M. tuberculosis* and is important in mycobacterial pathogenesis. | (Forrellad et al., 2013) |
| Complex cell envelope/Intracellular survival |  | *eccE5* | *Rv1797* | - | ESX-5 type VII secretion system protein EccE | *eccE5* belongs to ESX-5 system, which is involved in secretion of most PE-PPE proteins in *M. tuberculosis* and is important in mycobacterial pathogenesis. | (Forrellad et al., 2013) |
| Complex cell envelope/Intracellular survival |  | *eccA5* | *Rv1798* | COG1223R | ESX-5 type VII secretion system protein EccA | *eccE5* belongs to ESX-5 system, which is involved in secretion of most PE-PPE proteins in *M. tuberculosis* and is important in mycobacterial pathogenesis. | (Forrellad et al., 2013) |
| Complex cell envelope/Intracellular survival |  | *espD* | *Rv3614c* | - | ESX-1 secretion-associated protein EspD | *espD* plays an important role in the maintenance of cellular levels of EspA in *M. tuberculosis* and the secretion of EsxA. | (Forrellad et al., 2013) |
| Complex cell envelope/Intracellular survival | Hyper virulent | *mycP1* | *Rv3883c* | COG1404O | membrane-anchored mycosin | Serine protease MycP1 post-transcriptionally regulates the ESX-1 secretion activity, contributes to the fine-tuning of ESAT6 and CFP10 secretion,balancing the virulence and immunogenic properties of these proteins,which is essential for successful maintenance of long-term M. tuberculosis infection. | (Forrellad et al., 2013) |
| Complex cell envelope/Intracellular survival/Immune responsse | Hyper virulent/interferantimicrobial activity of the phagosome | *espE* | *Rv3864* | - | ESX-1 secretion-associated protein EspE | ESX systems function as specialized secretion systems that enable the transport of selected substrates across the complex, thick mycobacterial cell envelope that forms a structural barrier to protein export. ESX-1 secretion system are found to exhibit a range of diverse phenotypes, including defects in immune modulation, tissue invasion, phagosomal trafficking and growth inside the macrophages. | (Sreejit et al., 2014, Groschel et al., 2016) |
| Complex cell envelope/Intracellular survival/Immune responsse | Hyper virulent/interferantimicrobial activity of the phagosome | *espF* | *Rv3865* | - | ESX-1 secretion-associated protein EspF | ESX systems function as specialized secretion systems that enable the transport of selected substrates across the complex, thick mycobacterial cell envelope that forms a structural barrier to protein export. ESX-1 secretion system are found to exhibit a range of diverse phenotypes, including defects in immune modulation, tissue invasion, phagosomal trafficking and growth inside the macrophages. | (Sreejit et al., 2014, Groschel et al., 2016) |
| Complex cell envelope/Intracellular survival/Immune responsse | Hyper virulent/interferantimicrobial activity of the phagosome | *espG1* | *Rv3866* | COG4170V | ESX-1 secretion-associated protein EspG | ESX systems function as specialized secretion systems that enable the transport of selected substrates across the complex, thick mycobacterial cell envelope that forms a structural barrier to protein export. ESX-1 secretion system are found to exhibit a range of diverse phenotypes, including defects in immune modulation, tissue invasion, phagosomal trafficking and growth inside the macrophages. | (Sreejit et al., 2014, Groschel et al., 2016) |
| Complex cell envelope/Intracellular survival/Immune responsse | Hyper virulent/interferantimicrobial activity of the phagosome | *espH* | *Rv3867* | - | ESX-1 secretion-associated protein EspH | ESX systems function as specialized secretion systems that enable the transport of selected substrates across the complex, thick mycobacterial cell envelope that forms a structural barrier to protein export. ESX-1 secretion system are found to exhibit a range of diverse phenotypes, including defects in immune modulation, tissue invasion, phagosomal trafficking and growth inside the macrophages. | (Sreejit et al., 2014, Groschel et al., 2016) |
| Complex cell envelope/Intracellular survival/Immune responsse | Hyper virulent/interferantimicrobial activity of the phagosome | *eccA1* | *Rv3868* | COG0464O | ESX-1 secretion system protein EccA1 | ESX systems function as specialized secretion systems that enable the transport of selected substrates across the complex, thick mycobacterial cell envelope that forms a structural barrier to protein export. ESX-1 secretion system are found to exhibit a range of diverse phenotypes, including defects in immune modulation, tissue invasion, phagosomal trafficking and growth inside the macrophages. | (Sreejit et al., 2014, Groschel et al., 2016) |
| Complex cell envelope/Intracellular survival/Immune responsse | Hyper virulent/interferantimicrobial activity of the phagosome | *esxB* | *Rv3874* | COG4842S | ESAT-6-like protein EsxB | ESX systems function as specialized secretion systems that enable the transport of selected substrates across the complex, thick mycobacterial cell envelope that forms a structural barrier to protein export. ESX-1 secretion system are found to exhibit a range of diverse phenotypes, including defects in immune modulation, tissue invasion, phagosomal trafficking and growth inside the macrophages. | (Sreejit et al., 2014, Groschel et al., 2016) |
| Complex cell envelope/Intracellular survival/Immune responsse | Hyper virulent/interferantimicrobial activity of the phagosome | *esxA* | *Rv3875* | COG4842S | ESAT-6 protein EsxA | ESX systems function as specialized secretion systems that enable the transport of selected substrates across the complex, thick mycobacterial cell envelope that forms a structural barrier to protein export. ESX-1 secretion system are found to exhibit a range of diverse phenotypes, including defects in immune modulation, tissue invasion, phagosomal trafficking and growth inside the macrophages. | (Sreejit et al., 2014, Groschel et al., 2016) |
| Complex cell envelope/Intracellular survival/Immune responsse | Hyper virulent/interferantimicrobial activity of the phagosome | *eccD1* | *Rv3877* | - | ESX-1 secretion system protein EccD1 | ESX systems function as specialized secretion systems that enable the transport of selected substrates across the complex, thick mycobacterial cell envelope that forms a structural barrier to protein export. ESX-1 secretion system are found to exhibit a range of diverse phenotypes, including defects in immune modulation, tissue invasion, phagosomal trafficking and growth inside the macrophages. | (Sreejit et al., 2014, Groschel et al., 2016) |
| Complex cell envelope/Intracellular survival/Immune responsse | Hyper virulent/interferantimicrobial activity of the phagosome | *espJ* | *Rv3878* | - | ESX-1 secretion-associated protein EspJ | ESX systems function as specialized secretion systems that enable the transport of selected substrates across the complex, thick mycobacterial cell envelope that forms a structural barrier to protein export. ESX-1 secretion system are found to exhibit a range of diverse phenotypes, including defects in immune modulation, tissue invasion, phagosomal trafficking and growth inside the macrophages. | (Sreejit et al., 2014, Groschel et al., 2016) |
| Complex cell envelope/Intracellular survival/Immune responsse | Hyper virulent/interferantimicrobial activity of the phagosome | *espL* | *Rv3880c* | - | ESX-1 secretion-associated protein EspL | ESX systems function as specialized secretion systems that enable the transport of selected substrates across the complex, thick mycobacterial cell envelope that forms a structural barrier to protein export. ESX-1 secretion system are found to exhibit a range of diverse phenotypes, including defects in immune modulation, tissue invasion, phagosomal trafficking and growth inside the macrophages. | (Sreejit et al., 2014, Groschel et al., 2016) |
| Complex cell envelope/Intracellular survival/Immune responsse | Hyper virulent/interferantimicrobial activity of the phagosome | *espB* | *Rv3881c* | COG0038P | ESX-1 secretion-associated protein EspB | ESX systems function as specialized secretion systems that enable the transport of selected substrates across the complex, thick mycobacterial cell envelope that forms a structural barrier to protein export. ESX-1 secretion system are found to exhibit a range of diverse phenotypes, including defects in immune modulation, tissue invasion, phagosomal trafficking and growth inside the macrophages. | (Sreejit et al., 2014, Groschel et al., 2016) |
| Complex cell envelope/Intracellular survival/Immune responsse | Hyper virulent/interferantimicrobial activity of the phagosome | *eccE1* | *Rv3882c* | - | ESX-1 secretion system protein EccE1 | ESX systems function as specialized secretion systems that enable the transport of selected substrates across the complex, thick mycobacterial cell envelope that forms a structural barrier to protein export. ESX-1 secretion system are found to exhibit a range of diverse phenotypes, including defects in immune modulation, tissue invasion, phagosomal trafficking and growth inside the macrophages. | (Sreejit et al., 2014, Groschel et al., 2016) |
| dormancy |  | *PE4* | *Rv0160c* | - | PE family protein PE4 | Rv0160c is an in vivo expressed immunodominant antigen that could play an important role in intracellular persistence of the mycobacteria. | (Singh et al., 2013) |
| dormancy |  | *Rv0890c* | *Rv0890c* | COG3903R | HTH-type transcriptional regulator | Rv0890c is a LuxR family regulator involved in MTB dormancy. | (Liu et al., 2016) |
| dormancy |  | *pknD* | *Rv0931c* | COG0515RTKL | serine/threonine-protein kinase PknD | PknD is required for the survival and persistence of M. tuberculosis inside hosts. | (Forrellad et al., 2013) |
| dormancy |  | *PPE15* | *Rv1039c* | COG5651N | PPE family protein PPE15 | PPE15 is required for triacylglycerol accumulation under dormancy-inducing conditions. | (Daniel et al., 2016) |
| dormancy |  | *narG* | *Rv1161* | COG5013C | nitrate reductase subunit alpha | NarGH-mediated nitrate reductase activity is involved in *M. tuberculosis* virulence and persistence by protecting hypoxic mycobacteria from acid killing, an environmental stress encountered by the pathogen in inflamed granulomatous lesions and cavities. | (Tan et al., 2010) |
| dormancy |  | *narH* | *Rv1162* | COG1140C | nitrate reductase subunit beta | NarGH-mediated nitrate reductase activity is involved in *M. tuberculosis* virulence and persistence by protecting hypoxic mycobacteria from acid killing, an environmental stress encountered by the pathogen in inflamed granulomatous lesions and cavities. | (Tan et al., 2010) |
| dormancy |  | *TB8.4* | *Rv1174c* | - | low molecular weight T-cell antigen | TB8.4 is involved in reactivation of dormant mycobacteria. | (Bottai et al., 2006) |
| dormancy |  | *bioA* | *Rv1568* | COG0161H | adenosylmethionine-8-amino-7-oxononanoate aminotransferase | BioA was essential for the establishment of infection as well as for the persistence of *M. tuberculosis* in mice. | (Kar et al., 2017) |
| dormancy |  | *PE17* | *Rv1646* | - | PE family protein PE17 | PE17 is involved in persistant and possibly funciton within human macrophage-like cell line. | (Wang et al., 2011) |
| dormancy |  | *Rv1733c* | *Rv1733c* | - | transmembrane protein | A major *M. tuberculosis* latency antigen which is highly expressed by “dormant” *M. tuberculosis* and well recognized by T cells from latently M. tuberculosis-infected individuals. | (Coppola et al., 2015) |
| dormancy |  | *Rv1734c* | *Rv1734c* | COG0508C | hypothetical protein | Interestingly, Rv1734c is a dormancy regulon gene that is weakly induced early in NRP but is the only dormancy gene that was noticed to remain expressed in the late stages of NRP. | (Magombedze et al., 2013) |
| dormancy |  | *hspX* | *Rv2031c* | COG0071O | alpha-crystallin | HspX is part of the DosR regulon, a genetic program of *M. tuberculosis* induced by conditions that inhibit aerobic respiration and prevent bacillus replication that regulates the expression of a large number of dormancy-associated proteins | (Forrellad et al., 2013) |
| dormancy |  | *PE23* | *Rv2328* | - | PE family protein PE23 | PE23 is postulated to contribute to the microbe’s persistence in granulomas | (Robinson, 2007) |
| dormancy |  | *PPE41* | *Rv2430c* | COG5651N | PPE family protein PPE41 | PPE41 is involved in persistant and possibly funciton within human macrophage-like cell line. | (Wang et al., 2011) |
| dormancy |  | *PE26* | *Rv2519* | - | PE family protein PE26 | PE26 is involved in persistant and possibly funciton within human macrophage-like cell line. | (Wang et al., 2011) |
| dormancy |  | *PE27* | *Rv2769c* | - | PE family protein PE27 | PE27 is involved in persistant and possibly funciton within human macrophage-like cell line. | (Wang et al., 2011) |
| dormancy |  | *nuoG* | *Rv3151* | COG1034C | NADH-quinone oxidoreductase subunit G | nuoG encoding for the main subunit type I NADH-dehydrogenase, which confer anti-apoptosis activity and contribute to the survival and persistence of *Mycobacterium tuberculosis.* | (Velmurugan et al., 2007) |
| dormancy/Immune responsse |  | *PE3* | *Rv0159c* | - | PE family protein PE3 | PE3 plays significant roles in mycobacterial persistence during infection, modulate host immune response. | (Singh et al., 2013) |
| Immune responsse |  | *PPE1* | *Rv0096* | COG5651N | PPE family protein PPE1 | PPE1 may have a function in modulation of the host-bacterium interaction. | (Raman et al., 2006) |
| Immune responsse |  | *pepA* | *Rv0125* | COG0265O | serine protease PepA | PepA induces a proliferative PBMC response. | (Hebert et al., 2007) |
| Immune responsse |  | *Rv0146* | *Rv0146* | COG3315Q | S-adenosylmethionine-dependent methyltransferase | Rv0146 is a gene involved in the innate responses of primary macrophagesa gene involved in the innate responses of primary macrophages | (Kato-Maeda et al., 2013) |
| Immune responsse | Affecting host cytokine production | *lipC* | *Rv0220* | COG0657I | esterase LipC | Rv0220 is a cell‐surface‐associated esterase of *M. tuberculosis*, which was highly immunogenic and could elicit production of both antibodies and cytokines/chemokines. | (You et al., 2017) |
| Immune responsse |  | *PPE2* | *Rv0256c* | COG5651N | PPE family protein PPE2 | Rv0256c displays stronger and specific immunoreactivity against the sera obtained from clinically active tuberculosis patients compared to PPD and ESAT-6 and could differentiate the TB-patients from the BCG-vaccinated controls. | (Abraham et al., 2014) |
| Immune responsse |  | *Rv0309* | *Rv0309* | COG3786S | hypothetical protein | Rv0309 might be involved in diversifying selection to evade host immunity. | (Jiang et al., 2013) |
| Immune responsse |  | *groEL2* | *Rv0440* | COG0459O | molecular chaperone GroEL | GroEL2 is a highly expressed, immunodominant stress-induced protein. | (Forrellad et al., 2013) |
| Immune responsse |  | *ufaA1* | *Rv0447c* | COG2230M | cyclopropane-fatty-acyl-phospholipid synthase UfaA | Rv0447c, Rv2957 and Rv2958c participate in orchestrating clinically relevant immune responses in human TB by potentiating CD4+ and CD8+ T-cell responses. | (Rao et al., 2017) |
| Immune responsse |  | *rplL* | *Rv0652* | COG0222J | 50S ribosomal protein L7/L12 | Rv0652 stimulates production of tumour necrosis factor and monocytes chemoattractant protein-1 in macrophages through the Toll-like receptor 4 pathway. It may play an immune-stimulatory role during *M. tuberculosis* infection. | (Kim et al., 2012) |
| Immune responsse | protective immunity | *PPE14* | *Rv0915c* | COG5651N | PPE family protein PPE14 | PPE14 is proved to confer protective immunity against a challenge with *M. tuberculosis* in murine experimental modelsl | (Rindi et al., 2007) |
| Immune responsse |  | *rpfB* | *Rv1009* | COG3583S | resuscitation-promoting factor RpfB | RpfB regulates innate immunity and activates adaptive immunity through TLR4l | (Kim et al., 2013) |
| Immune responsse | Affecting host cytokine production | *PPE17* | *Rv1168c* | COG5651N | PPE family protein PPE17 | Rv1168 (PPE17) induces proinflammatory signaling. Rv1168c was also found to display stronger and specific immunoreactivity against the sera obtained from clinically active TB patients compared to PPD, ESAT-6 and *hsp*60.49. | (Mukhopadhyay and Balaji, 2011) |
| Immune responsse | Affecting host cytokine production | *PE13* | *Rv1195* | - | PE family protein PE13 | PE13 is foundtophysically interact with PPE18, which is found to strongly activate IL-10 induction that promotes replication and persistence of *M. tuberculosis* inside the host, and inhibits production of the protective IL-12 and TNF-a cytokines in activated macrophages by upregulating and phosphorylating SOCS3 protein | (Mukhopadhyay and Balaji, 2011) |
| Immune responsse |  | *Rv1255c* | *Rv1255c* | COG1309K | HTH-type transcriptional regulator | Rv1255c-E could evoke high Th1 responses and that Rv1255c-E may be more immunogenic shares homology with several helix-turn-helix-type DNA-binding transcriptional regulatorsl | (Luo et al., 2017) |
| Immune responsse |  | *tlyA* | *Rv1694* | COG1189J | 16S/23S rRNA (cytidine-2'-O)-methyltransferase TlyA | TlyA mutant *M.tuberculosis* indeuces enhanced host-potective Th1 and Th17 responses. TlyA significantly contributes to the pathogenesis of *M. tuberculosis*. | (Forrellad et al., 2013) |
| Immune responsse |  | *PPE22* | *Rv1705c* | COG5651N | PPE family protein PPE22 | Eptiope exasmination suggest the immunogeneic nature of PPE22. | (Fishbein et al., 2015) |
| Immune responsse |  | *Rv1769* | *Rv1769* | COG3616E | hypothetical protein | Rv1769 is one of eight highly immunogenic antigens that were identified as potential diagnostic reagents or as subunit vaccines warrant further study. | (Cockle et al., 2002) |
| Immune responsse |  | *PE20* | *Rv1806* | - | PE family protein PE20 | PE20-derived DNA vaccines have been shown to reduce guinea pig bacterial lung burden by >0.5 log. | (Sampson, 2011) |
| Immune responsse |  | *garA* | *Rv1827* | COG1716T | glycogen accumulation regulator GarA | garA is a substrate for PknB Human T cell antigen. | (Deenadayalan et al., 2010) |
| Immune responsse | protective immunity | *apa* | *Rv1860* | COG5373S | alanine/proline-rich secreted protein Apa | The glycosylated Rv1860 protein of *Mycobacterium tuberculosis* inhibits dendritic cell mediated TH1 and TH17 polarization of T Cells and abrogates protective immunity conferred by BCG. | (Satchidanandam et al., 2014) |
| Immune responsse |  | *mpt63* | *Rv1926c* | - | immunogenic protein Mpt63 | Mpt63 is a novel antigen. | (Manca et al., 1997) |
| Immune responsse |  | *Rv1979c* | *Rv1979c* | - | permease | Rv1979 is one of eight highly immunogenic antigens that were identified as potential diagnostic reagents or as subunit vaccines warrant further study. | (Cockle et al., 2002) |
| Immune responsse |  | *cfp21* | *Rv1984c* | COG3946U | cutinase | CFP21 and MPT64(rCM) fusion protein, encoded by RD2 of Mycobacterium tuberculosis, could stimulate higher level of interferon (IFN)-γ in tuberculin skin test (TST)-positive healthy population than in TST-negative healthy population. | (Wang et al., 2011) |
| Immune responsse |  | *Rv1986* | *Rv1986* | COG1279R | amino-acid transporter | Rv1986 is one of eight highly immunogenic antigens that were identified as potential diagnostic reagents or as subunit vaccines warrant further study. | (Cockle et al., 2002) |
| Immune responsse |  | *PPE36* | *Rv2108* | COG5651N | PPE family protein PPE36 | PPE36 is a cell wall associated protein, which might play a role in host-pathogen interactions. | (Mukhopadhyay and Balaji, 2011) |
| Immune responsse |  | *Rv2190c* | *Rv2190c* | COG0791M | endopeptidase | The rv2190c DNA had some immunotherapeutic effect on TB. | (Liang et al., 2017) |
| Immune responsse |  | *PE25* | *Rv2431c* | - | PE family protein PE25 | PE25 is involved in induce significant B cell response. | (Mukhopadhyay and Balaji, 2011) |
| Immune responsse |  | *PPE42* | *Rv2608* | COG5651N | PPE family protein PPE42 | PPE42 could induce both humoral and cellular immune responses. | (Ates et al., 2018) |
| Immune responsse |  | *Rv2654c* | *Rv2654c* | - | antitoxin | Rv2654 is a strongly recognized T cell antigen that is highly specific for TB. | (Aagaard et al., 2004) |
| Immune responsse |  | *PPE43* | *Rv2768c* | COG5651N | PPE family protein PPE43 | PPE43 is extracellular proteins that are either surface membrane attached or released proteins, and thus, may be directly involved in host-pathogen interaction. | (Chiliza et al., 2017) |
| Immune responsse |  | *PPE44* | *Rv2770c* | COG5651N | PPE family protein PPE44 | PPE44-specific immune responses could be detected in mice acutely, chronically and latently infected with *M. tuberculosis*. | (Romano et al., 2008) |
| Immune responsse |  | *mpt70* | *Rv2875* | COG2335M | major secreted immunogenic protein Mpt70 | MPT70 is an antigenic protein. *M. tuberculosis* produces small quantities of the antigen MPT70 in vitro and only induces expression during infection. | (Veyrier et al., 2008) |
| Immune responsse |  | *mpt53* | *Rv2878c* | COG1225O | soluble secreted antigen Mpt53 | MPT53 of *M. tuberculosis* induces strong, tuberculosis-specific antibody responses in guinea pigs but induces no delayed-type hypersensitivity. Involvement in immune responses during human tuberculosis was very modest. | (Johnson et al., 2001) |
| Immune responsse |  | *lepB* | *Rv2903c* | COG0681U | signal peptidase | Rv2903c contains a novel HLA-B*35-restricted CD8+ T-cell epitope in *Mycobacterium tuberculosis*, indicating its immunogeneic nature. | (Klein et al., 2002) |
| Immune responsse |  | *lipY* | *Rv3097c* | COG0657I | triacylglycerol lipase Lip | LipY can regulate intracellular triglyceride levels and is also exported to the cell wall by one of the ESX family members, ESX-5. It may interact with the host immune system. | (Mishra et al., 2008) |
| Immune responsse |  | *sigH* | *Rv3223c* | COG1595K | ECF RNA polymerase sigma factor SigH | *sigH* modulates the immune response elicited against *M. tuberculosis*. |  |
| Immune responsse |  | *esxT* | *Rv3444c* | COG4842S | ESAT-6 like protein EsxT | EsxT could induce apoptosis. | (Shi et al., 2014) |
| Immune responsse |  | *sodA* | *Rv3846* | COG0605P | superoxide dismutase | The secretion of SodA is likely to be the major SecA2-dependent process involved in the inhibition of host cell apoptosis. | (Forrellad et al., 2013) |
| Immune responsse | Affecting host cytokine production | *PPE27* | *Rv1790* | COG5651N | PPE family protein PPE27 | PPE27 promotes the survival of nonpathogenic *M. smegmatis* in vitro by manipulating the expression of multiple cytokines, NO, and affecting host cell necrosis, which provide a new insight to understand the functions of this gene. | (Yang et al., 2017) |
| Intracellular survival | Host-cell entry | *mce1A* | *Rv0169* | COG1463Q | Mce family protein Mce1A | Belong to *mce*1 operon, which conferring mycobacteria the ability to enter into mammalian cells and survive inside the macrophage | (Forrellad et al., 2013) |
| Intracellular survival | Host-cell entry | *mce1B* | *Rv0170* | COG1463Q | Mce family protein Mce1B | Belong to *mce*1 operon, which conferring mycobacteria the ability to enter into mammalian cells and survive inside the macrophage. | (Forrellad et al., 2013) |
| Intracellular survival | Host-cell entry | *mce1C* | *Rv0171* | COG1463Q | Mce family protein Mce1C | Belong to *mce*1 operon, which conferring mycobacteria the ability to enter into mammalian cells and survive inside the macrophage. | (Forrellad et al., 2013) |
| Intracellular survival | Host-cell entry | *mce1D* | *Rv0172* | COG1463Q | Mce family protein Mce1D | Belong to *mce*1 operon, which conferring mycobacteria the ability to enter into mammalian cells and survive inside the macrophage. | (Forrellad et al., 2013) |
| Intracellular survival | Host-cell entry | *mce1F* | *Rv0174* | COG1463Q | Mce family protein Mce1F | Belong to *mce*1 operon, which conferring mycobacteria the ability to enter into mammalian cells and survive inside the macrophage. | (Forrellad et al., 2013) |
| Intracellular survival | Nutrient absorption | *zmp1* | *Rv0198c* | COG3590O | zinc metalloprotease | *zmp1* gene is required for the survival of M. tuberculosis in murine J774 and RAW264.7 macrophages and for the full virulence of *M. tuberculosis.*It is also essential for prevention of inflammasome activation (a specialized inflammatory caspase activating protein complex, and component of the innate immune system) and IL-1β production. | (Forrellad et al., 2013) |
| Intracellular survival | Nutrient absorption | *Rv0203* | *Rv0203* | - | hypothetical protein | Rv0203 involved in mycobacterial heme-iron uptake and contribute to *Mycobacterium tuberculosis* survival. | (Owens et al., 2013) |
| Intracellular survival |  | *mmpL3* | *Rv0206c* | COG2409R | transmembrane transport protein MmpL3 | MmpL3 is essential for growth and survival of *M. tuberculosis* during human macrophage infection and MmpL3 depletion leads to a complex change in the global transcriptional profile. | (Degiacomi et al., 2017) |
| Intracellular survival |  | *fadE5* | *Rv0244c* | COG1960I | acyl-CoA dehydrogenase FadE5 | Rv0244c is required for in vitro growth on cholesterol. |  |
| Intracellular survival | stress response | *hsp* | *Rv0251c* | COG0071O | heat shock protein | Belong to a group of seven MTB genes that are up-regulated in response to multiple stresses. | (Wilkinson et al., 2005) |
| Intracellular survival | stress response | *sodC* | *Rv0432* | COG2032P | superoxide dismutase | SodC is a coppercontaining SOD outer-membrane lipoprotein involved in the protection of *M. tuberculosis* against the extracellular superoxide generated by host cells. | (Forrellad et al., 2013) |
| Intracellular survival | Interferantimicrobial activity of the phagosome | *PPE10* | *Rv0442c* | - | PPE family protein PPE10 | *ppe10* is relevant for *M. bovis BCG* to arrest phagosome acidification following uptake by macrophages.avoiding phagosomal acidification is important for the intracellular survival and persistence of pathogenic mycobacteria. | (Forrellad et al., 2013) |
| Intracellular survival |  | *mmpS4* | *Rv0451c* | - | membrane protein MmpS4 | MmpS4 is required for growth of Mtb under low iron conditions. | (Wells et al., 2013) |
| Intracellular survival | stress response | *senX3* | *Rv0490* | COG5002T | two component sensor histidine kinase SenX3 | SenX3-RegX3 is involved in phosphate sensing and is homologous to the master aerobic regulator ArcB-ArcA of *E. coli*. They are are necessary for *M. tuberculosis* full virulence. | (Forrellad et al., 2013) |
| Intracellular survival | Host-cell entry | *mce2A* | *Rv0589* | COG1463Q | Mce family protein Mce2A | Belong to *mce2* operon, which have evolved to fulfil specific roles, most likely related to lipid metabolism, modulating pathogenicity through changes in *M. tuberculosis* lipid pathways. | (Forrellad et al., 2013) |
| Intracellular survival | Host-cell entry | *mce2C* | *Rv0591* | COG1463Q | Mce family protein Mce2C | Belong to mce2 operon, which have evolved to fulfil specific roles, most likely related to lipid metabolism, modulating pathogenicity through changes in M. tuberculosis lipid pathways. | (Forrellad et al., 2013) |
| Intracellular survival | Host-cell entry | *mce2D* | *Rv0592* | COG1463Q | Mce family protein Mce2D | Belong to mce2 operon, which have evolved to fulfil specific roles, most likely related to lipid metabolism, modulating pathogenicity through changes in M. tuberculosis lipid pathways. | (Forrellad et al., 2013) |
| Intracellular survival | Host-cell entry | *mce2F* | *Rv0594* | COG1463Q | Mce family protein Mce2F | Belong to mce2 operon, which have evolved to fulfil specific roles, most likely related to lipid metabolism, modulating pathogenicity through changes in M. tuberculosis lipid pathways. | (Forrellad et al., 2013) |
| Intracellular survival | stress response | *PE_PGRS11* | *Rv0754* | COG0406G | PE-PGRS family protein PE_PGRS11 | PE_PGRS11 is an immunodominant antigen that plays a crucial role in modulating alveolar epithelial cell fate decisions under oxidative stress. | (Chaturvedi et al., 2010) |
| Intracellular survival |  | *phoP* | *Rv0757* | COG0745TK | two component system response transcriptional positive regulator PhoP | The two-component system PhoP/PhoR is the one whose disruption has been shown to most dramatically affect the ability of *M. tuberculosis* to replicate in cellular and animal models. | (Forrellad et al., 2013) |
| Intracellular survival |  | *phoR* | *Rv0758* | COG0642T | two component system response sensor kinase PhoR | The two-component system PhoP/PhoR is the one whose disruption has been shown to most dramatically affect the ability of *M. tuberculosis* to replicate in cellular and animal models. | (Forrellad et al., 2013) |
| Intracellular survival |  | *prrB* | *Rv0902c* | COG0642T | two component sensor histidine kinase PrrB | The mutation of TCS prrA-prrB diminishes the *M. tuberculosis* growth in macrophages at initial stages. | (Forrellad et al., 2013) |
| Intracellular survival |  | *prrA* | *Rv0903c* | COG0745TK | two component transcriptional regulator PrrA | The mutation of TCS prrA-prrB diminishes the *M. tuberculosis* growth in macrophages at initial stages. | (Forrellad et al., 2013) |
| Intracellular survival |  | *glmU* | *Rv1018c* | COG1207M | bifunctional UDP-N-acetylglucosamine pyrophosphorylase/glucosamine-1-phosphate N-acetyltransferase | GlmU is a substrate for two important biosynthetic pathways: lipopolysaccharide and peptidoglycan synthesis. The *glmU* gene is essential in *Mycobacterium tuberculosis*, being required for optimal bacterial growth. | (Zhang et al., 2009) |
| Intracellular survival | Nutrient absorption | *kdpE* | *Rv1027c* | COG0745TK | transcriptional regulator KdpE | The two-component system KdpD/KdpE plays a regulatory role in potassium (K+) transportis and is an adaptive regulator involved in the virulence and intracellular survival of pathogenic bacteria. | (Freeman et al., 2013) |
| Intracellular survival | Nutrient absorption | *kdpD* | *Rv1028c* | COG2205T | sensor protein KdpD | The two-component system KdpD/KdpE plays a regulatory role in potassium (K+) transportis and is an adaptive regulator involved in the virulence and intracellular survival of pathogenic bacteria. | (Freeman et al., 2013) |
| Intracellular survival | stress response | *narJ* | *Rv1163* | COG2180C | nitrate reductase subunit delta | *narGHJI* contribute to anaerobic growth in the presence of nitrate. | (Freeman et al., 2013) |
| Intracellular survival | stress response | *narI* | *Rv1164* | COG2181C | nitrate reductase subunit gamma | *narGHJI* contribute to anaerobic growth in the presence of nitrate. | (Sohaskey and Wayne, 2003) |
| Intracellular survival |  | *rho* | *Rv1297* | COG1158K | transcription termination factor Rho | Rho is a bona fidemolecular motor and directional helicase which requires a catalytic site competent for ATP hydrolysis to disrupt RNA duplexes or transcription elongation complexes. Rho appears to be essential for *M. tuberculosis* growth. | (D'Heygere et al., 2015) |
| Intracellular survival |  | *PE15* | *Rv1386* | - | PE family protein PE15 | PE15 (Rv1386) is associated with the cell envelope and modulates innate immunity and mediate bacillary survival in macrophages. | (Tiwari et al., 2012) |
| Intracellular survival | stress response | *Rv1405c* | *Rv1405c* | COG2226H | methyltransferase | *M. tuberculosis* Rv1405c mutants attenuated growth in response to in vitro stress conditions that mimic the intracellular milieuthe. It plays a role in this adaptive process. | (Healy et al., 2016) |
| Intracellular survival |  | *Rv1461* | *Rv1461* | COG0719O | hypothetical protein | The Rv1461 encodes the primary Fe-S cluster biogenesis system, is predicted to be essential for in vitro growth of M. tuberculosis. | (Willemse et al., 2018) |
| Intracellular survival | host-cell entry | *Rv1490* | *Rv1490* | - | membrane protein | Rv1490 could be participating in mycobacterial host-cell entry and thus contributing to mycobacterial pathogenicity. | (Patarroyo et al., 2008) |
| Intracellular survival |  | *pykA* | *Rv1617* | COG0469G | pyruvate kinase | PykA encodes an active pyruvate kinase that is allosterically activated by glucose-6-phosphate (G6P) and adenosine monophosphate (AMP). Deletion of pykA prevents Mtb growth in the presence of fermentable carbon sources and has a cidal effect in the presence of glucose that correlates with elevated levels of the toxic catabolite methylglyoxal. | (Noy et al., 2016) |
| Intracellular survival |  | *mgtC* | *Rv1811* | COG1285S | Mg2+ transport P-type ATPase MgtC | MgtC mutant was attenuated for virulence in cultured human macrophages and impaired for growth in the lungs and spleens of BALB/c mice compared with the parental or complemented strains. | (Forrellad et al., 2013) |
| Intracellular survival | stress response | *glcB* | *Rv1837c* | COG2225C | malate synthase | GlcB takes part in the glyoxylate shunt and has been implicated as a virulence factor, which was also important for MTB survival under adverse conditions, such as low oxygen, nonreplicative states and the intracellular environment. | (Liu et al., 2016) |
| Intracellular survival |  | *lldD2* | *Rv1872c* | COG1304C | L-lactate dehydrogenase | ΔlldD2 mutant was impaired in replication in human macrophages, indicating a critical role for lactate oxidation during intracellular growth. | (Billig et al., 2017) |
| Intracellular survival | host-cell entry | *mce3A* | *Rv1966* | COG1463Q | Mce family protein Mce3A | Mce protein an important protein family for entry and survival of bacteria in the host. | (Jia et al., 2017) |
| Intracellular survival | host-cell entry | *mce3B* | *Rv1967* | COG1463Q | Mce family protein Mce3B | Mce protein an important protein family for entry and survival of bacteria in the host. | (Jia et al., 2017) |
| Intracellular survival | host-cell entry | *mce3D* | *Rv1969* | COG1463Q | Mce family protein Mce3D | Mce protein an important protein family for entry and survival of bacteria in the host. | (Jia et al., 2017) |
| Intracellular survival | host-cell entry | *mce3F* | *Rv1971* | COG1463Q | Mce family protein Mce3F | Mce protein an important protein family for entry and survival of bacteria in the host. | (Jia et al., 2017) |
| Intracellular survival |  | *sigC* | *Rv2069* | COG1595K | ECF RNA polymerase sigma factor SigC | SigC is important for pathogenesis and survival within granulomas in low-dose aerosol guinea pig infection model: *sigC*-mutant produced fewer and smaller lung and spleen granulomas as compared with the parental *M. tuberculosis*. | (Forrellad et al., 2013) |
| Intracellular survival |  | *tatA* | *Rv2094c* | COG1826U | Sec-independent protein translocase membrane-bound protein TatA | Tat export is essential for growth of *M. tuberculosis*, at least under standard laboratory conditions, as shown by the inability to delete tatA, tatB, or tatC unless exogenous copies of the tat genes are provided. | (Feltcher et al., 2010) |
| Intracellular survival | stress response | *ctaC* | *Rv2200c* | COG1622C | cytochrome C oxidase subunit II | CtaC is the subunit II of the cytochrome c oxidase important for growth under aerobic conditions. It is essential in *M. tuberculosis* H37Rv and has a role in virulence. | (Forrellad et al., 2013) |
| Intracellular survival | Nutrient absorption | *mbtG* | *Rv2378c* | COG3486Q | L-lysine N6-monooxygenase | Proteins involved in iron acquisition, siderophores are the most important iron-chelating compounds synthetised by microorganisms, being mycobactin and carboxymycobactin the major ones in Mycobacterium. These compounds are biosynthesized through the action of proteins encoded by the mbt cluster, which includes the genes mbtA to mbtJ. The mutation of any mbt gene disrupts the synthesis of these siderophores, which, in turn, unables the bacterium to acquire the metal from the medium. Therefore, the bacteria fail to survive in the host cell. | (Forrellad et al., 2013) |
| Intracellular survival | Nutrient absorption | *mbtF* | *Rv2379c* | COG1020Q | peptide synthetase | Proteins involved in iron acquisition, siderophores are the most important iron-chelating compounds synthetised by microorganisms, being mycobactin and carboxymycobactin the major ones in Mycobacterium. These compounds are biosynthesized through the action of proteins encoded by the mbt cluster, which includes the genes mbtA to mbtJ. The mutation of any mbt gene disrupts the synthesis of these siderophores, which, in turn, unables the bacterium to acquire the metal from the medium. Therefore, the bacteria fail to survive in the host cell. | (Forrellad et al., 2013) |
| Intracellular survival | Nutrient absorption | *mbtE* | *Rv2380c* | - | peptide synthetase | Proteins involved in iron acquisition, siderophores are the most important iron-chelating compounds synthetised by microorganisms, being mycobactin and carboxymycobactin the major ones in Mycobacterium. These compounds are biosynthesized through the action of proteins encoded by the mbt cluster, which includes the genes mbtA to mbtJ. The mutation of any mbt gene disrupts the synthesis of these siderophores, which, in turn, unables the bacterium to acquire the metal from the medium. Therefore, the bacteria fail to survive in the host cell. | (Forrellad et al., 2013) |
| Intracellular survival | Nutrient absorption | *mbtD* | *Rv2381c* | COG3321Q | polyketide synthetase | Proteins involved in iron acquisition, siderophores are the most important iron-chelating compounds synthetised by microorganisms, being mycobactin and carboxymycobactin the major ones in Mycobacterium. These compounds are biosynthesized through the action of proteins encoded by the mbt cluster, which includes the genes mbtA to mbtJ. The mutation of any mbt gene disrupts the synthesis of these siderophores, which, in turn, unables the bacterium to acquire the metal from the medium. Therefore, the bacteria fail to survive in the host cell. | (Forrellad et al., 2013) |
| Intracellular survival | Nutrient absorption | *mbtC* | *Rv2382c* | COG3321Q | polyketide synthetase | Proteins involved in iron acquisition, siderophores are the most important iron-chelating compounds synthetised by microorganisms, being mycobactin and carboxymycobactin the major ones in Mycobacterium. These compounds are biosynthesized through the action of proteins encoded by the mbt cluster, which includes the genes mbtA to mbtJ. The mutation of any mbt gene disrupts the synthesis of these siderophores, which, in turn, unables the bacterium to acquire the metal from the medium. Therefore, the bacteria fail to survive in the host cell. | (Forrellad et al., 2013) |
| Intracellular survival | nutrient absorption | *mbtB* | *Rv2383c* | COG1020Q | phenyloxazoline synthase | Proteins involved in iron acquisition, siderophores are the most important iron-chelating compounds synthetised by microorganisms, being mycobactin and carboxymycobactin the major ones in Mycobacterium. These compounds are biosynthesized through the action of proteins encoded by the mbt cluster, which includes the genes mbtA to mbtJ. The mutation of any mbt gene disrupts the synthesis of these siderophores, which, in turn, unables the bacterium to acquire the metal from the medium. Therefore, the bacteria fail to survive in the host cell. | (Forrellad et al., 2013) |
| Intracellular survival | Nutrient absorption | *mbtA* | *Rv2384* | COG1021Q | bifunctional salicyl-AMP ligase/salicyl-S-ArCP synthetase | Proteins involved in iron acquisition, siderophores are the most important iron-chelating compounds synthetised by microorganisms, being mycobactin and carboxymycobactin the major ones in Mycobacterium. These compounds are biosynthesized through the action of proteins encoded by the mbt cluster, which includes the genes mbtA to mbtJ. The mutation of any mbt gene disrupts the synthesis of these siderophores, which, in turn, unables the bacterium to acquire the metal from the medium. Therefore, the bacteria fail to survive in the host cell. | (Forrellad et al., 2013) |
| Intracellular survival | Nutrient absorption | *mbtJ* | *Rv2385* | COG0657I | acetyl hydrolase | Proteins involved in iron acquisition, siderophores are the most important iron-chelating compounds synthetised by microorganisms, being mycobactin and carboxymycobactin the major ones in Mycobacterium. These compounds are biosynthesized through the action of proteins encoded by the mbt cluster, which includes the genes mbtA to mbtJ. The mutation of any mbt gene disrupts the synthesis of these siderophores, which, in turn, unables the bacterium to acquire the metal from the medium. Therefore, the bacteria fail to survive in the host cell. | (Forrellad et al., 2013) |
| Intracellular survival | Nutrient absorption | *mbtI* | *Rv2386c* | COG0147EH | salicylate synthase | Proteins involved in iron acquisition, siderophores are the most important iron-chelating compounds synthetised by microorganisms, being mycobactin and carboxymycobactin the major ones in Mycobacterium. These compounds are biosynthesized through the action of proteins encoded by the mbt cluster, which includes the genes mbtA to mbtJ. The mutation of any mbt gene disrupts the synthesis of these siderophores, which, in turn, unables the bacterium to acquire the metal from the medium. Therefore, the bacteria fail to survive in the host cell. | (Forrellad et al., 2013) |
| Intracellular survival |  | *relA* | *Rv2583c* | COG0317TK | bifunctional (p)ppGpp synthase/hydrolase RelA | The *rel*-deficient mutant showed impaired initial growth and survival relative to the wild-type strain. Loss of Rel was associated with the striking absence of tubercle lesions grossly and of caseous granulomas histologically. | (Klinkenberg et al., 2010) |
| Intracellular survival |  | *leuD* | *Rv2987c* | COG0066E | 3-isopropylmalate dehydratase small subunit | *leuD*, encoding isopropylmalate isomerase, an enzyme that functions in the biosynthesis of leucine.The *leuD* mutant could not grow in primary murine macrophages or kill SCID mice. | (Smith, 2003) |
| Intracellular survival |  | *ftsX* | *Rv3101c* | COG2177D | cell division protein FtsX | FtsX recognizes RipC and links the cell division apparatus with peptidoglycan hydrolysis.*ftsX* is involved in Mtb growth and division. | (Mavrici et al., 2014) |
| Intracellular survival | stress response | *devS* | *Rv3132c* | COG4585T | two component sensor histidine kinase DevS | The *Mycobacterium tuberculosis* dosS gene (Rv3133c) is part of an operon, Rv3134c-Rv3132c, and encodes a response regulator that has been shown to be upregulated by hypoxia and other in vitro stress conditions and may be important for bacterial survival within granulomatous lesions found in tuberculosis.*dosS* is required for full virulence | (Converse et al., 2009) |
| Intracellular survival | stress response | *devR* | *Rv3133c* | COG2197TK | two component transcriptional regulator DevR | The *Mycobacterium tuberculosis* dosR gene (Rv3133c) is part of an operon, Rv3134c-Rv3132c, and encodes a response regulator that has been shown to be upregulated by hypoxia and other in vitro stress conditions and may be important for bacterial survival within granulomatous lesions found in tuberculosis.*dosR* is required for full virulence. | (Converse et al., 2009) |
| Intracellular survival | stress response | *whiB1* | *Rv3219* | - | transcriptional regulator WhiB1 | *M. tuberculosis* WhiB1 is an essential (Smith et al.) protein that acts as a specific NO-sensing DNA-binding protein. NO sensing and gene expression reprogramming by WhiB1 could contribute to developmental adaptations in response to host generated NO. | (Smith et al., 2010) |
| Intracellular survival |  | *ctpC* | *Rv3270* | COG2217P | manganese/zinc-exporting P-type ATPase | *ctpC* deficiency caused zinc retention within the mycobacterial cytoplasm, leading to impaired intracellular growth of the bacilli. | (Botella et al., 2011) |
| Intracellular survival | Interferantimicrobial activity of the phagosome | *sapM* | *Rv3310* | - | acid phosphatase | Mtb ΔsapM is defective in the arrest of phagosomal maturation as well as for growth in human THP-1 macrophages. | (Puri et al., 2013) |
| Intracellular survival |  | *espA* | *Rv3616c* | - | ESX-1 secretion-associated protein EspA | EspA is related to the growth capability of mutants. | (Forrellad et al., 2013) |
| Intracellular survival |  | *fbpA* | *Rv3804c* | COG0627R | diacylglycerol acyltransferase/mycolyltransferase Ag85A | The mutant of *fbpA* can induce a marked decrease in growth in monocyte-like human THP-1 and murine J774 macrophage cell lines.FbpA would be essential in the virulence of M. tuberculosis. | (Forrellad et al., 2013) |
| Intracellular survival | stress response | *dnaK* | *Rv0350* | COG0443O | chaperone protein DnaK | DnaK may indirectly be responding to heat stress in a feedback loop mechanism. Overexpression of DnaK in M. tuberculosis leads to an enhanced clearance of the pathogen in a mouse model. | (Parijat and Batra, 2015) |
| Intracellular survival | stress response | *mprA* | *Rv0981* | - | two-component response regulator MrpA | MprAB system senses and processes stress resulting from the accumulation of unfolded or misfolded protein substrates and regulate the expression of chaperones/proteases like HtrA2.This complex regulatory system is required for full virulence. | (Forrellad et al., 2013) |
| Intracellular survival | stress response | *mprB* | *Rv0982* | COG0642T | two component histidine-protein kinase/phosphatase MprB | MprAB system senses and processes stress resulting from the accumulation of unfolded or misfolded protein substrates and regulate the expression of chaperones/proteases like HtrA2.This complex regulatory system is required for full virulence. | (Forrellad et al., 2013) |
| Intracellular survival | stress response | *sigE* | *Rv1221* | COG1595K | ECF RNA polymerase sigma factor SigE | SigE is one of the major regulators involved in the mycobacterial stress responses and it is also upregulated upon macrophage infection. | (Forrellad et al., 2013) |
| Intracellular survival | stress response | *TB15.3* | *Rv1636* | COG0589T | iron-regulated universal stress protein | Rv1636(TB15.3) is an universal stress protein with unknown function. These were over-expressed under nutrient/oxygen limitation and be involved in virulence/chronic infection. | (Sharma et al., 2016) |
| Intracellular survival | stress response | *bfrA* | *Rv1876* | COG2193P | bacterioferritin BfrA | Constitutive expression of bfrA modestly enhanced resistance to oxidative stress suggesting that BfrA may function under oxidative stress. | (Pandey and Rodriguez, 2014) |
| Intracellular survival | stress response | *ahpC* | *Rv2428* | COG0450O | alkyl hydroperoxide reductase subunit C | AhpC is involved in the response to oxidative stress in *M. tuberculosis*. *ahpC* is located adjacent to oxyR, a central regulator of the peroxydative and nitrosative stress response that is dysfunctional in the MTBC species. What’s more, M. tuberculosis compensates the lack of KatG catalase-peroxidase activity overexpressing AhpC. | (Forrellad et al., 2013) |
| Intracellular survival | stress response | *lipF* | *Rv3487c* | - | carboxylesterase LipF | LipF may function to cleave lipids which could provide metabolic energy to withstand acidic stress. Specific induction of lipF may prime mycobacteria to be more resistant to acidic stress and be more likely to survive in vivo. | (Richter and Saviola, 2009) |
| Intracellular survival/Immune responsse | stress response | *Rv1813c* | *Rv1813c* | - | hypothetical protein | Rv1813c and Rv1812c may represent general stress-responsive elements that are necessary for aspects of *M. tuberculosis* virulence and the host immune response to infection. | (Bretl et al., 2012) |
| virulence |  | *sugA* | *Rv1236* | COG1175G | sugar ABC transporter permease SugA | The LpqY-SugA-SugB-SugC transporter-mediated uptake of trehalose plays crucial important role in the virulence of *M.tuberculosis*. | (Forrellad et al., 2013) |
| virulence |  | *sugB* | *Rv1237* | COG0395G | sugar ABC transporter permease SugB | The LpqY-SugA-SugB-SugC transporter-mediated uptake of trehalose plays crucial important role in the virulence of *M. tuberculosis*. | (Forrellad et al., 2013) |
| virulence |  | *sugC* | *Rv1238* | COG3839G | sugar ABC transporter ATP-binding protein SugC | The LpqY-SugA-SugB-SugC transporter-mediated uptake of trehalose plays crucial important role in the virulence of *M. tuberculosis*. | (Forrellad et al., 2013) |
| Virulence |  | *PPE21* | *Rv1548c* | COG5651N | PPE family protein PPE21 | PPE21 is involved in lipid metabolism, an important feature of mycobacterial pathogenicity. | (Fishbein et al., 2015) |
| Virulence |  | *Rv0249c* | *Rv0249c* | - | succinate dehydrogenase membrane anchor subunit | Rv0249c encodes for virulence-associated succinate dehydrogenase. | (Knapp et al., 2015) |
| Virulence |  | *PE5* | *Rv0285* | - | PE family protein PE5 | PE5–PPE4 was found to be critical for the siderophore-mediated iron-acquisition functions of ESX-3. | (Tufariello, 2016 #32) |
| Virulence |  | *PPE4* | *Rv0286* | COG5651N | PPE family protein PPE4 | PE5–PPE4 was found to be critical for the siderophore-mediated iron-acquisition functions of ESX-3. | (Tufariello, 2016 #32) |
| Virulence |  | *aspC* | *Rv0337c* | COG0436E | aspartate aminotransferase | *aspC* gene is thought to be essential in *M. tuberculosis* and is involved mostly in aspartate biosynthesis. | (Gouzy et al., 2013) |
| Virulence |  | *hbhA* | *Rv0475* | COG1754R | heparin binding hemagglutinin HbhA | HbhA (heparin-binding hemagglutinin) is the major adhesin exposed at the surface of the cell. It is required for extrapulmonary dissemination, and that interactions with non-phagocytic cells have an important role in the pathogenesis of tuberculosis. | (Forrellad et al., 2013) |
| Virulence |  | *proC* | *Rv0500* | COG0345E | pyrroline-5-carboxylate reductase | ProC is involved in proline biosynthesis. | (Smith, 2003) |
| Virulence |  | *cmaA2* | *Rv0503c* | COG2230M | cyclopropane mycolic acid synthase | *cmaA2*is required for trans cyclopropane formation. | (Barkan et al., 2010) |
| Virulence |  | *mscL* | *Rv0985c* | COG1970M | large-conductance ion mechanosensitive channel | MscL directly senses and responds to membrane tension, and prevents cell lysis. | (Zhong and Blount, 2013) |
| Virulence |  | *papA3* | *Rv1182* | - | acyltransferase PapA | PapA3 is involved in lipid metabolism. | (Forrellad et al., 2013) |
| Virulence |  | *mmpL10* | *Rv1183* | COG2409R | transmembrane transport protein MmpL10 | MmpL10, transport protein belonging to the RND superfamily, is involved in lipid metabolism. | (Forrellad et al., 2013) |
| Virulence |  | *atpH* | *Rv1307* | COG0712C | ATP synthase subunit b/delta | *atpH* is a component of the ATP synthase complex. Which is involved in aerobic respiration. | (Lin et al., 2016) |
| Virulence |  | *atpA* | *Rv1308* | COG0056C | ATP synthase subunit alpha | *atp*A is a component of the ATP synthase complex. Which is involved in aerobic respiration. | (Lin et al., 2016) |
| Virulence |  | *atpD* | *Rv1310* | COG0055C | ATP synthase subunit beta | *atpD* is a component of the ATP synthase complex. Which is involved in aerobic respiration. | (Lin et al., 2016) |
| Virulence |  | *atpC* | *Rv1311* | COG0355C | ATP synthase subunit epsilon | *atpC* is a component of the ATP synthase complex. Which is involved in aerobic respiration. | (Lin et al., 2016) |
| Virulence |  | *mbtK* | *Rv1347c* | COG1670J | lysine N-acetyltransferase MbtK | MbtK is involved in iron uptake is present in the *M. tuberculosis*. | (Forrellad et al., 2013) |
| Virulence |  | *irtA* | *Rv1348* | COG1132V | iron ABC transporter ATP-binding protein/permease IrtA | IrtAB is a transporter of the Fe3+-siderophore complex.The *irtAB* mutant shows a reduced ability to replicate in THP-1 human macrophages and in the lungs of C57B/6 mice compared with the parental strain fused to a cytoplasm substrate-binding domain (SBD), which is essential for iron acquisition. | (Forrellad et al., 2013) |
| Virulence |  | *irtB* | *Rv1349* | COG1132V | iron ABC transporter ATP-binding protein/permease IrtB | IrtAB is a transporter of the Fe3+-siderophore complex.The *irtAB* mutant shows a reduced ability to replicate in THP-1 human macrophages and in the lungs of C57B/6 mice compared with the parental strain fused to a cytoplasm substrate-binding domain (SBD), which is essential for iron acquisition. | (Forrellad et al., 2013) |
| Virulence |  | *narX* | *Rv1736c* | COG5013C | nitrate reductase-like protein NarX | NarX is predicted to encode a nitrate reductase which may help the bacterium to adapt to hypoxia, which is controlled by dosR. | (Hu and Coates, 2011) |
| Virulence |  | *narK2* | *Rv1737c* | COG2223P | nitrate/nitrite transporter | DosR is the master regulator of "DosR regulon", which regulates the expression of narK2 that are induced when Mtb encounters hypoxic conditions. | (Koo et al., 2012) |
| Virulence |  | *eccB5* | *Rv1782* | - | ESX-5 type VII secretion system protein EccB5 | EccB5, a transmembrane protein, was predicted to be building blocks of the *M. tuberculosis* ESX-5 membrane-associated complex. | (Di Luca et al., 2012) |
| Virulence |  | *PE19* | *Rv1791* | - | PE family protein PE19 | Mutants in ppe25-pe19 are attenuated both in BMDM and in the SCID mouse infection model. | (Forrellad et al., 2013) |
| Virulence |  | *PPE35* | *Rv1918c* | COG5651N | PPE family protein PPE35 | PPE35 may play additional roles in the mechanisms of pathogenesis. | (Bannantine et al., 2011) |
| Virulence |  | *mpt64* | *Rv1980c* | - | immunogenic protein Mpt64 | MPT64 could inhibit the apoptosis of RAW264.7 macrophages through the NF-κB-miRNA21-Bcl-2 pathway. | (Wang et al., 2014) |
| Virulence |  | *pafA* | *Rv2097c* | - | proteasome accessory factor PafA | PafA is essential to cause lethal infections by *Mycobacterium tuberculosis* (Mtb) in mice. |  |
| Virulence |  | *mpa* | *Rv2115c* | COG0464O | proteasome-associated ATPase | Mpa is improtant for defense against RNI and for virulence of Mtb in mouose. | (Forrellad et al., 2013) |
| Virulence |  | *glnA1* | *Rv2220* | COG0174E | glutamine synthetase | GlnA1 gene encodes a GSI enzyme that is transcriptionally and posttranslationally regulated in a manner similar to that of the Escherichia coli GS. It is essential for *M. tuberculosis* virulence. | (Tullius et al., 2003) |
| Virulence |  | *caeA* | *Rv2224c* | COG0596R | carboxylesterase A | CaeA is required for full virulence of M. tuberculosis in mice. | (Forrellad et al., 2013) |
| Virulence |  | *ptpA* | *Rv2234* | COG0394T | protein-tyrosine-phosphatase | PtpA is a member of mycobacterial proteins involved in arresting macrophage phagosomal maturation with a clear role in the virulence of pathogenic Mycobacterium species. | (Forrellad et al., 2013) |
| Virulence |  | *kasB* | *Rv2246* | COG0304IQ | 3-oxoacyl-ACP synthase 2 | *kasB* gene is involved in the pathogenesis of *M. tuberculosis.* | (Forrellad et al., 2013) |
| Virulence |  | *plcC* | *Rv2349c* | COG3511M | phospholipase C | PlcC is a phospholipase C-type enzyme, which plays a role in the virulence of *M. tuberculosis*. | (Forrellad et al., 2013) |
| Virulence |  | *eis* | *Rv2416c* | COG4552R | enhanced intracellular survival protein | Eis plays essential roles in modulating macrophage autophagy, inflammatory responses, and cell death via a reactive oxygen species (ROS)-dependent pathway. Macrophages infected with an Mtb eis-deletion mutant H37Rv (Mtb-Δeis) displayed markedly increased accumulation of massive autophagic vacuoles and formation of autophagosomes in vitro and in vivo. | (Shin et al., 2010) |
| Virulence |  | *ideR* | *Rv2711* | COG1321K | iron-dependent repressor and activator IdeR | IdeR is an iron-dependent regulatory protein essential in *M. tuberculosis* that functions as a repressor. In an abundant iron condition, IdeR is found complexed with Fe3+, and this complex binds to the promoter regions of mbt and mbt-2 clusters preventing their transcription. On the contrary, in a depleted iron condition, there is not sufficient iron to form the Fe3+-IdeR complex, and IdeR releases the promoter leading to the transcription of the genes of this cluster which, as was described ealier, leads to the synthesis of proteins essential for the acquisition and incorporation of iron to the bacteria. | (Forrellad et al., 2013) |
| Virulence |  | *fadE22* | *Rv3061c* | COG1960I | acyl-CoA dehydrogenase FadE22 | *fadE22* plays a potential pathogenic role in *Mycobacterium tuberculosis*. | (Rindi et al., 2002) |
| Virulence |  | *lipR* | *Rv3084* | COG0657I | acetyl-hydrolase LipR | *lipR*have a role in tuberculosis transmission and pathogenesis. | (Sheline et al., 2009) |
| Virulence |  | *fadD13* | *Rv3089* | COG0318IQ | long chain-fatty-acid--CoA ligase FadD13 | *fadE13*p lays a potential pathogenic role in *Mycobacterium tuberculosis*. | (Rindi et al., 2002) |
| Virulence |  | *moaA1* | *Rv3109* | COG2896H | cyclic pyranopterin monophosphate synthase 1 | moaD2 contributes to MoCo production in *M. tuberculosis*. MoCo biosynthesis is important in the physiology and pathogenesis of *M. tuberculosis*. | (Williams et al., 2011) |
| Virulence |  | *ppk2* | *Rv3232c* | COG2326S | polyphosphate kinase | The stringent response factor PPK2 play an important role in *M. tuberculosis* metabolism, biofilm formation, and antibiotic sensitivity in vivo. | (Chuang et al., 2016) |
| Virulence |  | *sigF* | *Rv3286c* | COG1191K | RNA polymerase sigma factor SigF | A *sigF* mutant of *M. tuberculosis* H37Rv produced diffused granulomas lacking necrosis in guinea pig lungs. | (Forrellad et al., 2013) |
| Virulence |  | *sigD* | *Rv3414c* | COG1595K | ECF RNA polymerase sigma factor SigD | *sigD* may play a role in the tuberculosis disease. | (Forrellad et al., 2013) |
| Virulence |  | *mce4F* | *Rv3494c* | COG1463Q | Mce family protein Mce4 | *mce4F* belongs to mce4 operon. *Mycobacterium tuberculosis* strains disrupted in mce4 operon is attenuated in mice. | (Senaratne et al., 2008) |
| Virulence |  | *mce4D* | *Rv3496c* | COG1463Q | Mce family protein Mce4D | *mce4D* belongs to mce4 operon. *Mycobacterium tuberculosis* strains disrupted in mce4 operon is attenuated in mice. | (Senaratne et al., 2008) |
| Virulence |  | *mce4C* | *Rv3497c* | COG1463Q | Mce family protein Mce4C | *mce4C* belongs to mce4 operon. *Mycobacterium tuberculosis* strains disrupted in mce4 operon is attenuated in mice. | (Senaratne et al., 2008) |
| Virulence |  | *mce4B* | *Rv3498c* | COG1463Q | Mce family protein Mce4B | *mce4B* belongs to mce4 operon. *Mycobacterium tuberculosis* strains disrupted in mce4 operon is attenuated in mice. | (Senaratne et al., 2008) |
| Virulence |  | *mce4A* | *Rv3499c* | COG1463Q | Mce family protein Mce4A | *mce4A* belongs to mce4 operon. *Mycobacterium tuberculosis* strains disrupted in mce4 operon is attenuated in mice. | (Senaratne et al., 2008) |
| Virulence |  | *mmpL8* | *Rv3823c* | COG2409R | integral membrane transport protein MmpL8 | MmpL8 is necessary for an intermediate step in the SL-1 biosynthesis pathway.MmpL8 transports other molecules that are implicated in virulence (other than SL-1). | (Forrellad et al., 2013) |
| Virulence and immune response |  | *PPE28* | *Rv1800* | COG5651N | PPE family protein PPE28 | The PPE genes chromosomally and functionally associated with ESX-5. | (Mendum et al., 2015) |
| Virulence and immune response |  | *PPE30* | *Rv1802* | COG5651N | PPE family protein PPE30 | The PPE genes chromosomally and functionally associated with ESX-5. | (Mendum et al., 2015) |
| Virulence and immune response |  | *PPE32* | *Rv1808* | COG5651N | PPE family protein PPE32 | The PPE genes chromosomally and functionally associated with ESX-5. | (Mendum et al., 2015) |
| Virulence and immune response |  | *PPE33* | *Rv1809* | COG5651N | PPE family protein PPE33 | The PPE genes chromosomally and functionally associated with ESX-5. | (Mendum et al., 2015) |
| Virulence and immune response |  | *fbpB* | *Rv1886c* | COG0627R | diacylglycerol acyltransferase/mycolyltransferase Ag85B | FbpB is a member of complex (Ag85), which is the major secreted protein constituent of mycobacterial cell culture and it is also found in association with the bacterial surface. This protein complex plays an essential role in the pathogenesis of tuberculosis. | (Forrellad et al., 2013) |
| Virulence and immune response |  | *espC* | *Rv3615c* | - | ESX-1 secretion-associated protein EspC | EspC is a potent antigen in both active and latent TB infection. T-cell responses to EspC were highly specific (93%) for M. tuberculosis infection. The immunodominance of EspC, equivalent to that of ESAT6 and CFP10, and its high antigenic specificity make this protein a promissory TB vaccine candidate and a potential T-cellantigen. | (Forrellad et al., 2013) |
| Virulence and immune response |  | *espR* | *Rv3849* | - | ESX-1 transcriptional regulator EspR | EspR can bind to the *espACD* operon promoter and is then secreted from *M.tuberculosis* by the ESX-1 system.And the *espACD* operon is under positive regulation control of EspR | (Forrellad et al., 2013) |

**Reference**

Aagaard, C., Brock, I., Olsen, A., Ottenhoff, T. H., Weldingh, K., and Andersen, P. (2004). Mapping immune reactivity toward Rv2653 and Rv2654: two novel low-molecular-mass antigens found specifically in the *Mycobacterium tuberculosis* complex. *J Infect Dis*. **189**, 812-819. doi: 10.1086/381679.

Abraham, P. R., Latha, G. S., Valluri, V. L., and Mukhopadhyay, S. (2014). *Mycobacterium tuberculosis* PPE protein Rv0256c induces strong b cell response in tuberculosis patients. *Infect Genet Evol*. **22**, 244-249. doi: 10.1016/j.meegid.2013.06.023.

Angala, S. K., Belardinelli, J. M., Huc-Claustre, E., Wheat, W. H., and Jackson, M. (2014). The cell envelope glycoconjugates of *Mycobacterium tuberculosis*. *Crit Rev Biochem Mol Biol*. **49**, 361-399. doi: 10.3109/10409238.2014.925420.

Ates, L. S., Sayes, F., Frigui, W., Ummels, R., Damen, M. P. M., Bottai, D., et al. (2018). RD5-mediated lack of PE_PGRS and PPE-MPTR export in BCG vaccine strains results in strong reduction of antigenic repertoire but little impact on protection. *PLoS Pathog*. **14**, e1007139. doi: 10.1371/journal.ppat.1007139.

Bannantine, J. P., Paulson, A. L., Chacon, O., Fenton, R. J., Zinniel, D. K., McVey, D. S., et al. (2011). Immunogenicity and reactivity of novel *Mycobacterium avium* subsp. paratuberculosis PPE MAP1152 and conserved MAP1156 proteins with sera from experimentally and naturally infected animals. *Clin Vaccine Immunol*. **18**, 105-112. doi: 10.1128/CVI.00297-10.

Barkan, D., Rao, V., Sukenick, G. D., and Glickman, M. S. (2010). Redundant function of *cmaA2* and *mmaA2* in *Mycobacterium tuberculosis* *cis* cyclopropanation of oxygenated mycolates. *J Bacteriol*. **192**, 3661-3668. doi: 10.1128/JB.00312-10.

Belardinelli, J. M., Larrouy-Maumus, G., Jones, V., Sorio de Carvalho, L. P., McNeil, M. R., and Jackson, M. (2014). Biosynthesis and translocation of unsulfated acyltrehaloses in *Mycobacterium tuberculosis*. *J Biol Chem*. **289**, 27952-27965. doi: 10.1074/jbc.M114.581199.

Billig, S., Schneefeld, M., Huber, C., Grassl, G. A., Eisenreich, W., and Bange, F. C. (2017). Lactate oxidation facilitates growth of *Mycobacterium tuberculosis* in human macrophages. *Sci Rep*. **7**, 6484. doi: 10.1038/s41598-017-05916-7.

Botella, H., Peyron, P., Levillain, F., Poincloux, R., Poquet, Y., Brandli, I., et al. (2011). Mycobacterial p(1)-type atpases mediate resistance to zinc poisoning in human macrophages. *Cell Host Microbe*. **10**, 248-259. doi: 10.1016/j.chom.2011.08.006.

Bottai, D., Batoni, G., Esin, S., Florio, W., Brancatisano, F. L., Favilli, F., et al. (2006). The secretion antigen SA5K has a role in the adaptation of *Mycobacterium bovis* bacillus Calmette-Guérin to intracellular stress and hypoxia. *Microbes Infect*. **8**, 2254-2261. doi: 10.1016/j.micinf.2006.04.020.

Bretl, D. J., He, H., Demetriadou, C., White, M. J., Penoske, R. M., Salzman, N. H., et al. (2012). MprA and DosR coregulate a *Mycobacterium tuberculosis* virulence operon encoding Rv1813c and Rv1812c. *Infect Immun*. **80**, 3018-3033. doi: 10.1128/IAI.00520-12.

Chaturvedi, R., Bansal, K., Narayana, Y., Kapoor, N., Sukumar, N., Togarsimalemath, S. K., et al. (2010). TThe multifunctional PE_PGRS11 protein from *Mycobacterium tuberculosis* plays a role in regulating resistance to oxidative stress. *J Biol Chem*. **285**, 30389-30403. doi: 10.1074/jbc.M110.135251.

Chiliza, T. E., Pillay, M., and Pillay, B. (2017). Identification of unique essential proteins from a mycobacterium tuberculosis f15/lam4/kzn phage secretome library. Pathog Dis. **75**. doi: 10.1093/femspd/ftx001.

Chuang, Y. M., Dutta, N. K., Hung, C. F., Wu, T. C., Rubin, H., and Karakousis, P. C. (2016). Stringent response factors PPX1 and PPK2 play an important role in *Mycobacterium tuberculosis* metabolism, biofilm formation, and sensitivity to isoniazid *in vivo*. *Antimicrob Agents Chemother*. **60**, 6460-6470. doi: 10.1128/AAC.01139-16.

Cockle, P. J., Gordon, S. V., Lalvani, A., Buddle, B. M., Hewinson, R. G., and Vordermeier, H. M. (2002). Identification of novel *Mycobacterium tuberculosis* antigens with potential as diagnostic reagents or subunit vaccine candidates by comparative genomics. *Infect Immun*. **70**, 6996-7003.

Converse, P. J., Karakousis, P. C., Klinkenberg, L. G., Kesavan, A. K., Ly, L. H., Allen, S. S., et al. (2009). Role of the *dosR-dosS* two-component regulatory system in *Mycobacterium tuberculosis* virulence in three animal models. *Infect Immun*. **77**, 1230-1237. doi: 10.1128/IAI.01117-08.

Coppola, M., van den Eeden, S. J., Wilson, L., Franken, K. L., Ottenhoff, T. H., and Geluk, A. (2015). Synthetic long peptide derived from *Mycobacterium tuberculosis* latency antigen Rv1733c protects against tuberculosis. *Clin Vaccine Immunol*. **22**, 1060-1069. doi: 10.1128/CVI.00271-15.

D'Heygere, F., Schwartz, A., Coste, F., Castaing, B., and Boudvillain, M. (2015). ATP-dependent motor activity of the transcription termination factor Rho from *Mycobacterium tuberculosis*. *Nucleic Acids Res*. **43**, 6099-6111. doi: 10.1093/nar/gkv505.

Daniel, J., Kapoor, N., Sirakova, T., Sinha, R., and Kolattukudy, P. (2016). The perilipin-like PPE15 protein in *Mycobacterium tuberculosis* is required for triacylglycerol accumulation under dormancy-inducing conditions. *Mol Microbiol*. **101**, 784-794. doi: 10.1111/mmi.13422.

Deenadayalan, A., Heaslip, D., Rajendiran, A. A., Velayudham, B. V., Frederick, S., Yang, H. L., et al. (2010). Immunoproteomic identification of human T cell antigens of *Mycobacterium tuberculosis* that differentiate healthy contacts from tuberculosis patients. *Mol Cell Proteomics*. **9**, 538-549. doi: 10.1074/mcp.M900299-MCP200.

Degiacomi, G., Benjak, A., Madacki, J., Boldrin, F., Provvedi, R., Palu, G., et al. (2017). Essentiality of *mmpL3* and impact of its silencing on *Mycobacterium tuberculosis* gene expression. *Sci Rep*. **7**, 43495. doi: 10.1038/srep43495.

Di Luca, M., Bottai, D., Batoni, G., Orgeur, M., Aulicino, A., Counoupas, C., et al. (2012). The ESX-5 associated *eccB_5_-eccC_5_* locus is essential for *Mycobacterium tuberculosis* viability. *PLoS One*. **7**, e52059. doi: 10.1371/journal.pone.0052059.

Drage, M. G., Pecora, N. D., Hise, A. G., Febbraio, M., Silverstein, R. L., Golenbock, D. T., et al. (2009). TLR2 and its co-receptors determine responses of macrophages and dendritic cells to lipoproteins of *Mycobacterium tuberculosis*. *Cell Immunol*. **258**, 29-37. doi: 10.1016/j.cellimm.2009.03.008.

Feltcher, M. E., Sullivan, J. T., and Braunstein, M. (2010). Protein export systems of *Mycobacterium tuberculosis*: novel targets for drug development? *Future Microbiol*. **5**, 1581-1597. doi: 10.2217/fmb.10.112.

Fishbein, S., van Wyk, N., Warren, R. M., and Sampson, S. L. (2015). Phylogeny to function: PE/PPE protein evolution and impact on *Mycobacterium tuberculosis* pathogenicity. *Mol Microbiol*. **96**, 901-916. doi: 10.1111/mmi.12981.

Forrellad, M. A., Klepp, L. I., Gioffre, A., Sabio y Garcia, J., Morbidoni, H. R., de la Paz Santangelo, M., et al. (2013). Virulence factors of the *Mycobacterium tuberculosis* complex. *Virulence*. **4**, 3-66. doi: 10.4161/viru.22329.

Freeman, Z. N., Dorus, S., and Waterfield, N. R. (2013). The KdpD/KdpE two-component system: integrating K^+^ homeostasis and virulence. *PLoS Pathog*. **9**, e1003201. doi: 10.1371/journal.ppat.1003201.

Giuffre, A., Borisov, V. B., Arese, M., Sarti, P., and Forte, E. (2014). Cytochrome *bd* oxidase and bacterial tolerance to oxidative and nitrosative stress. *Biochim Biophys Acta*. **1837**, 1178-1187. doi: 10.1016/j.bbabio.2014.01.016.

Gouzy, A., Poquet, Y., and Neyrolles, O. (2013). A central role for aspartate in *Mycobacterium tuberculosis* physiology and virulence. *Front Cell Infect Microbiol*. **3**, 68. doi: 10.3389/fcimb.2013.00068.

Groschel, M. I., Sayes, F., Simeone, R., Majlessi, L., and Brosch, R. (2016). Esx secretion systems: mycobacterial evolution to counter host immunity. *Nat Rev Microbiol*. **14**, 677-691. doi: 10.1038/nrmicro.2016.131.

Healy, C., Golby, P., MacHugh, D. E., and Gordon, S. V. (2016). The MarR family transcription factor Rv1404 coordinates adaptation of *Mycobacterium tuberculosis* to acid stress via controlled expression of Rv1405c, a virulence-associated methyltransferase. *Tuberculosis (Edinb)*. **97**, 154-162. doi: 10.1016/j.tube.2015.10.003.

Hebert, A. M., Talarico, S., Yang, D., Durmaz, R., Marrs, C. F., Zhang, L., et al. (2007). DNA polymorphisms in the *pepA* and *PPE18* genes among clinical strains of *Mycobacterium tuberculosis*: implications for vaccine efficacy. *Infect Immun*. **75**, 5798-5805. doi: 10.1128/IAI.00335-07.

Hu, Y., and Coates, A. R. (2011). *Mycobacterium tuberculosis* *acg* gene is required for growth and virulence in vivo. *PLoS One*. **6**, e20958. doi: 10.1371/journal.pone.0020958.

Jia, X., Yang, L., Dong, M., Chen, S., Lv, L., Cao, D., et al. (2017). The bioinformatics analysis of comparative genomics of *Mycobacterium tuberculosis* complex (MTBC) provides insight into dissimilarities between intraspecific groups differing in host association, virulence, and epitope diversity. *Front Cell Infect Microbiol*. **7**, 88. doi: 10.3389/fcimb.2017.00088.

Jiang, Y., Dou, X., Zhang, W., Liu, H., Zhao, X., Wang, H., et al. (2013). Genetic diversity of antigens Rv2945c and Rv0309 in *Mycobacterium tuberculosis* strains may reflect ongoing immune evasion. *FEMS Microbiol Lett*. **347**, 77-82. doi: 10.1111/1574-6968.12222.

Johnson, S., Brusasca, P., Lyashchenko, K., Spencer, J. S., Wiker, H. G., Bifani, P., et al. (2001). Characterization of the secreted MPT53 antigen of *Mycobacterium tuberculosis*. *Infect Immun*. **69**, 5936-5939.

Kar, R., Nangpal, P., Mathur, S., Singh, S., and Tyagi, A. K. (2017). *bioA* mutant of *Mycobacterium tuberculosis* shows severe growth defect and imparts protection against tuberculosis in guinea pigs. PLoS One. **12**, e0179513. doi: 10.1371/journal.pone.0179513.

Kato-Maeda, M., Ho, C., Passarelli, B., Banaei, N., Grinsdale, J., Flores, L., et al. (2013). Use of whole genome sequencing to determine the microevolution of *Mycobacterium tuberculosis* during an outbreak. *PLoS One*. **8**, e58235. doi: 10.1371/journal.pone.0058235.

Kawaguchi, H., Matsumoto, I., Osada, A., Kurata, I., Ebe, H., Tanaka, Y., et al. (2018). Identification of novel biomarker as citrullinated inter-alpha-trypsin inhibitor heavy chain 4, specifically increased in sera with experimental and rheumatoid arthritis. *Arthritis Res Ther*. **20**, 66. doi: 10.1186/s13075-018-1562-7.

Kim, J. S., Kim, W. S., Choi, H. G., Jang, B., Lee, K., Park, J. H., et al. (2013). *Mycobacterium tuberculosis* RpfB drives Th1-type T cell immunity via a TLR4-dependent activation of dendritic cells. *J Leukoc Biol*. **94**, 733-749. doi: 10.1189/jlb.0912435.

Kim, K., Sohn, H., Kim, J. S., Choi, H. G., Byun, E. H., Lee, K. I., et al. (2012). *Mycobacterium tuberculosis* Rv0652 stimulates production of tumour necrosis factor and monocytes chemoattractant protein-1 in macrophages through the Toll-like receptor 4 pathway. *Immunology*. **136**, 231-240. doi: 10.1111/j.1365-2567.2012.03575.x.

Klein, M. R., Hammond, A. S., Smith, S. M., Jaye, A., Lukey, P. T., and McAdam, K. P. (2002). Hla-b*35-restricted cd8(+)-T-cell epitope in *Mycobacterium tuberculosis* Rv2903c. Infect Immun. **70**, 981-984.

Klinkenberg, L. G., Lee, J. H., Bishai, W. R., and Karakousis, P. C. (2010). The stringent response is required for full virulence of *Mycobacterium tuberculosis* in guinea pigs. *J Infect Dis*. **202**, 1397-1404. doi: 10.1086/656524.

Knapp, G. S., Lyubetskaya, A., Peterson, M. W., Gomes, A. L., Ma, Z., Galagan, J. E., et al. (2015). Role of intragenic binding of cAMP responsive protein (CRP) in regulation of the succinate dehydrogenase genes Rv0249c-Rv0247c in TB complex mycobacteria. *Nucleic Acids Res*. **43**, 5377-5393. doi: 10.1093/nar/gkv420.

Koo, M. S., Subbian, S., and Kaplan, G. (2012). Strain specific transcriptional response in *Mycobacterium tuberculosis* infected macrophages. *Cell Commun Signal*. **10**, 2. doi: 10.1186/1478-811X-10-2.

Lai, X., Wu, J., Chen, S., Zhang, X., and Wang, H. (2008). Expression, purification, and characterization of a functionally active *Mycobacterium tuberculosis* UDP-glucose pyrophosphorylase. *Protein Expr Purif*. **61**, 50-56. doi: 10.1016/j.pep.2008.05.015.

Liang, Y., Zhang, X., Bai, X., Xiao, L., Wang, X., Zhang, J., et al. (2017). Immunogenicity and therapeutic effects of a *Mycobacterium tuberculosis* Rv2190c DNA vaccine in mice. *BMC Immunol*. **18**, 11. doi: 10.1186/s12865-017-0196-x.

Lin, W., de Sessions, P. F., Teoh, G. H., Mohamed, A. N., Zhu, Y. O., Koh, V. H., et al. (2016). Transcriptional profiling of *Mycobacterium tuberculosis* exposed to *in vitro* lysosomal stress. *Infect Immun*. **84**, 2505-2523. doi: 10.1128/IAI.00072-16.

Liu, Q., Luo, T., Dong, X., Sun, G., Liu, Z., Gan, M., et al. (2016). Genetic features of *Mycobacterium tuberculosis* modern Beijing sublineage. *Emerg Microbes Infect*. **5**, e14. doi: 10.1038/emi.2016.14.

Luo, L., Zhu, L., Yue, J., Liu, J., Liu, G., Zhang, X., et al. (2017). Antigens Rv0310c and Rv1255c are promising novel biomarkers for the diagnosis of *Mycobacterium tuberculosis* infection. *Emerg Microbes Infect*. **6**, e64. doi: 10.1038/emi.2017.54.

Magombedze, G., Dowdy, D., and Mulder, N. (2013). Latent tuberculosis: models, computational efforts and the pathogen's regulatory mechanisms during dormancy. *Front Bioeng Biotechnol*. **1**, 4. doi: 10.3389/fbioe.2013.00004.

Manca, C., Lyashchenko, K., Wiker, H. G., Usai, D., Colangeli, R., and Gennaro, M. L. (1997). Molecular cloning, purification, and serological characterization of MPT63, a novel antigen secreted by Mycobacterium tuberculosis. *Infect Immun*. **65**, 16-23.

Mavrici, D., Marakalala, M. J., Holton, J. M., Prigozhin, D. M., Gee, C. L., Zhang, Y. J., et al. (2014). *Mycobacterium tuberculosis* FtsX extracellular domain activates the peptidoglycan hydrolase, RipC. *Proc Natl Acad Sci U S A*. **111**, 8037-8042. doi: 10.1073/pnas.1321812111.

Mendum, T. A., Wu, H., Kierzek, A. M., and Stewart, G. R. (2015). Lipid metabolism and Type VII secretion systems dominate the genome scale virulence profile of *Mycobacterium tuberculosis* in human dendritic cells. *BMC Genomics*. **16**, 372. doi: 10.1186/s12864-015-1569-2.

Mishra, K. C., de Chastellier, C., Narayana, Y., Bifani, P., Brown, A. K., Besra, G. S., et al. (2008). Functional role of the PE domain and immunogenicity of the *Mycobacterium tuberculosis* triacylglycerol hydrolase Lipy. *Infect Immun*. **76**, 127-140. doi: 10.1128/IAI.00410-07.

Mukhopadhyay, S., and Balaji, K. N. (2011). The PE and PPE proteins of *Mycobacterium tuberculosis*. *Tuberculosis (Edinb)*. **91**, 441-447. doi: 10.1016/j.tube.2011.04.004.

Noy, T., Vergnolle, O., Hartman, T. E., Rhee, K. Y., Jacobs, W. R., Jr., Berney, M., et al. (2016). Central role of pyruvate kinase in carbon co-catabolism of *Mycobacterium tuberculosis*. *J Biol Chem*. **291**, 7060-7069. doi: 10.1074/jbc.M115.707430.

Owens, C. P., Chim, N., Graves, A. B., Harmston, C. A., Iniguez, A., Contreras, H., et al. (2013). The *Mycobacterium tuberculosis* secreted protein Rv0203 transfers heme to membrane proteins MmpL3 and MmpL11. *J Biol Chem*. **288**, 21714-21728. doi: 10.1074/jbc.M113.453076.

Pandey, R., and Rodriguez, G. M. (2014). IdeR is required for iron homeostasis and virulence in *Mycobacterium tuberculosis*. *Mol Microbiol*. **91**, 98-109. doi: 10.1111/mmi.12441.

Parijat, P., and Batra, J. K. (2015). Role of DnaK in HspR-HAIR interaction of *Mycobacterium tuberculosis*. *IUBMB Life*. **67**, 816-827. doi: 10.1002/iub.1438.

Patarroyo, M. A., Curtidor, H., Plaza, D. F., Ocampo, M., Reyes, C., Saboya, O., et al. (2008). Peptides derived from the *Mycobacterium tuberculosis* Rv1490 surface protein implicated in inhibition of epithelial cell entry: potential vaccine candidates? *Vaccine*. **26**, 4387-4395. doi: 10.1016/j.vaccine.2008.05.092.

Puri, R. V., Reddy, P. V., and Tyagi, A. K. (2013). Secreted acid phosphatase (SapM) of *Mycobacterium tuberculosis* is indispensable for arresting phagosomal maturation and growth of the pathogen in guinea pig tissues. *PLoS One*. **8**, e70514. doi: 10.1371/journal.pone.0070514.

Purwantini, E., Daniels, L., and Mukhopadhyay, B. (2016). F420H2 is required for phthiocerol dimycocerosate synthesis in mycobacteria. *J Bacteriol*. **198**, 2020-2028. doi: 10.1128/JB.01035-15.

Raman, S., Puyang, X., Cheng, T. Y., Young, D. C., Moody, D. B., and Husson, R. N. (2006). *Mycobacterium tuberculosis* SigM positively regulates Esx secreted protein and nonribosomal peptide synthetase genes and down regulates virulence-associated surface lipid synthesis. *J Bacteriol*. **188**, 8460-8468. doi: 10.1128/JB.01212-06.

Ramon-Garcia, S., Stewart, G. R., Hui, Z. K., Mohn, W. W., and Thompson, C. J. (2015). The mycobacterial P55 efflux pump is required for optimal growth on cholesterol. *Virulence*. **6**, 444-448. doi: 10.1080/21505594.2015.1044195.

Rao, M., Cadieux, N., Fitzpatrick, M., Reed, S., Arsenian, S., Valentini, D., et al. (2017). *Mycobacterium tuberculosis* proteins involved in cell wall lipid biosynthesis improve BCG vaccine efficacy in a murine tb model. *Int J Infect Dis*. **56**, 274-282. doi: 10.1016/j.ijid.2017.01.024.

Richter, L., and Saviola, B. (2009). The *lipF* promoter of *Mycobacterium tuberculosis* is upregulated specifically by acidic pH but not by other stress conditions. *Microbiol Res*. **164**, 228-232. doi: 10.1016/j.micres.2007.06.003.

Rindi, L., Fattorini, L., Bonanni, D., Iona, E., Freer, G., Tan, D., et al. (2002). Involvement of the *fadD33* gene in the growth of *Mycobacterium tuberculosis* in the liver of BALB/c mice. *Microbiology*. **148**, 3873-3880. doi: 10.1099/00221287-148-12-3873.

Rindi, L., Peroni, I., Lari, N., Bonanni, D., Tortoli, E., and Garzelli, C. (2007). Variation of the expression of *Mycobacterium tuberculosis ppe44* gene among clinical isolates. *FEMS Immunol Med Microbiol*. **51**, 381-387. doi: 10.1111/j.1574-695X.2007.00315.x.

Robinson, N. (2007). Identification of a novel mycobacterial gene involved in the synthesis of a phenolic glycolipid and its role in the prevention of phagosome maturation: University of Bonn.

Romano, M., Rindi, L., Korf, H., Bonanni, D., Adnet, P. Y., Jurion, F., et al. (2008). Immunogenicity and protective efficacy of tuberculosis subunit vaccines expressing PPE44 ( Rv2770c). *Vaccine*. **26**, 6053-6063. doi: 10.1016/j.vaccine.2008.09.025.

Rosas-Magallanes, V., Stadthagen-Gomez, G., Rauzier, J., Barreiro, L. B., Tailleux, L., Boudou, F., et al. (2007). Signature-tagged transposon mutagenesis identifies novel *Mycobacterium tuberculosis* genes involved in the parasitism of human macrophages. *Infect Immun*. **75**, 504-507. doi: 10.1128/IAI.00058-06.

Sampson, S. L. (2011). Mycobacterial PE/PPE proteins at the host-pathogen interface. *Clin Dev Immunol*. **2011**, 497203. doi: 10.1155/2011/497203.

Satchidanandam, V., Kumar, N., Jumani, R. S., Challu, V., Elangovan, S., and Khan, N. A. (2014). The glycosylated Rv1860 protein of *Mycobacterium tuberculosis* inhibits dendritic cell mediated TH1 and TH17 polarization of T cells and abrogates protective immunity conferred by BCG. *PLoS Pathog*. **10**, e1004176. doi: 10.1371/journal.ppat.1004176.

Senaratne, R. H., Sidders, B., Sequeira, P., Saunders, G., Dunphy, K., Marjanovic, O., et al. (2008). *Mycobacterium tuberculosis* strains disrupted in *mce3* and *mce4* operons are attenuated in mice. *J Med Microbiol*. **57**, 164-170. doi: 10.1099/jmm.0.47454-0.

Sharma, D., Lata, M., Singh, R., Deo, N., Venkatesan, K., and Bisht, D. (2016). Cytosolic proteome profiling of aminoglycosides resistant *Mycobacterium tuberculosis* clinical isolates using MALDI-TOF/MS. *Front Microbiol*. **7**, 1816. doi: 10.3389/fmicb.2016.01816.

Sheline, K. D., France, A. M., Talarico, S., Foxman, B., Zhang, L., Marrs, C. F., et al. (2009). Does the *lipR* gene of tubercle bacilli have a role in tuberculosis transmission and pathogenesis? *Tuberculosis (Edinb)*. **89**, 114-119. doi: 10.1016/j.tube.2008.09.004.

Shi, J., Zhang, H., Fang, L., Xi, Y., Zhou, Y., Luo, R., et al. (2014). A novel firefly luciferase biosensor enhances the detection of apoptosis induced by ESAT-6 family proteins of *Mycobacterium tuberculosis*. *Biochem Biophys Res Commun*. **452**, 1046-1053. doi: 10.1016/j.bbrc.2014.09.047.

Shin, D. M., Jeon, B. Y., Lee, H. M., Jin, H. S., Yuk, J. M., Song, C. H., et al. (2010). *Mycobacterium tuberculosis* eis regulates autophagy, inflammation, and cell death through redox-dependent signaling. *PLoS Pathog*. **6**, e1001230. doi: 10.1371/journal.ppat.1001230.

Simeone, R., Leger, M., Constant, P., Malaga, W., Marrakchi, H., Daffe, M., et al. (2010). Delineation of the roles of FadD22, FadD26 and FadD29 in the biosynthesis of phthiocerol dimycocerosates and related compounds in *Mycobacterium tuberculosis*. *FEBS J*. **277**, 2715-2725. doi: 10.1111/j.1742-464X.2010.07688.x.

Singh, S. K., Kumari, R., Singh, D. K., Tiwari, S., Singh, P. K., Sharma, S., et al. (2013). Putative roles of a proline-glutamic acid-rich protein (PE3) in intracellular survival and as a candidate for subunit vaccine against *Mycobacterium tuberculosis*. *Med Microbiol Immunol*. **202**, 365-377. doi: 10.1007/s00430-013-0299-9.

Singh, S. K., Tripathi, D. K., Singh, P. K., Sharma, S., and Srivastava, K. K. (2013). Protective and survival efficacies of Rv0160c protein in murine model of *Mycobacterium tuberculosis*. *Appl Microbiol Biotechnol*. **97**, 5825-5837. doi: 10.1007/s00253-012-4493-2.

Slama, N., Jamet, S., Frigui, W., Pawlik, A., Bottai, D., Laval, F., et al. (2016). The changes in mycolic acid structures caused by hadc mutation have a dramatic effect on the virulence of *Mycobacterium tuberculosis*. *Molecular Microbiology*. **99**, 794-807. doi: 10.1111/mmi.13266.

Smith, I. (2003). *Mycobacterium tuberculosis* pathogenesis and molecular determinants of virulence. *Clin Microbiol Rev*. **16**, 463-496.

Smith, L. J., Stapleton, M. R., Fullstone, G. J., Crack, J. C., Thomson, A. J., Le Brun, N. E., et al. (2010). *Mycobacterium tuberculosis* WhiB1 is an essential DNA-binding protein with a nitric oxide-sensitive iron-sulfur cluster. *Biochem J*. **432**, 417-427. doi: 10.1042/BJ20101440.

Sohaskey, C. D., and Wayne, L. G. (2003). Role of *narK2X* and *narGHJI* in hypoxic upregulation of nitrate reduction by *Mycobacterium tuberculosis*. *J Bacteriol*. **185**, 7247-7256.

Sreejit, G., Ahmed, A., Parveen, N., Jha, V., Valluri, V. L., Ghosh, S., et al. (2014). The ESAT-6 protein of Mycobacterium tuberculosis interacts with beta-2-microglobulin (β2M) affecting antigen presentation function of macrophage. *PLoS Pathog*. **10**, e1004446. doi: 10.1371/journal.ppat.1004446.

Tan, M. P., Sequeira, P., Lin, W. W., Phong, W. Y., Cliff, P., Ng, S. H., et al. (2010). Nitrate respiration protects hypoxic *Mycobacterium tuberculosis* against acid- and reactive nitrogen species stresses. *PLoS One*. **5**, e13356. doi: 10.1371/journal.pone.0013356.

Tiwari, B. M., Kannan, N., Vemu, L., and Raghunand, T. R. (2012). The *Mycobacterium tuberculosis* PE proteins Rv0285 and Rv1386 modulate innate immunity and mediate bacillary survival in macrophages. *PLoS One*. **7**, e51686. doi: 10.1371/journal.pone.0051686.

Touchette, M. H., Bommineni, G. R., Delle Bovi, R. J., Gadbery, J. E., Nicora, C. D., Shukla, A. K., et al. (2015). Diacyltransferase activity and chain length specificity of *Mycobacterium tuberculosis* PapA5 in the synthesis of alkyl beta-diol lipids. *Biochemistry*. **54**, 5457-5468. doi: 10.1021/acs.biochem.5b00455.

Touchette, M. H., Holsclaw, C. M., Previti, M. L., Solomon, V. C., Leary, J. A., Bertozzi, C. R., et al. (2015). The *rv1184c* locus encodes Chp2, an acyltransferase in *Mycobacterium tuberculosis* polyacyltrehalose lipid biosynthesis. *J Bacteriol*. **197**, 201-210. doi: 10.1128/JB.02015-14.

Tschumi, A., Grau, T., Albrecht, D., Rezwan, M., Antelmann, H., and Sander, P. (2012). Functional analyses of mycobacterial lipoprotein diacylglyceryl transferase and comparative secretome analysis of a mycobacterial lgt mutant. *J Bacteriol*. **194**, 3938-3949. doi: 10.1128/JB.00127-12.

Tufariello, J. M., Chapman, J. R., Kerantzas, C. A., Wong, K. W., Vilcheze, C., Jones, C. M., et al. (2016). Separable roles for *Mycobacterium tuberculosis* ESX-3 effectors in iron acquisition and virulence. *Proc Natl Acad Sci U S A*. **113**, E348-357. doi: 10.1073/pnas.1523321113.

Tullius, M. V., Harth, G., and Horwitz, M. A. (2003). Glutamine synthetase GlnA1 is essential for growth of *Mycobacterium tuberculosis* in human THP-1 macrophages and guinea pigs. *Infect Immun*. **71**, 3927-3936.

Velmurugan, K., Chen, B., Miller, J. L., Azogue, S., Gurses, S., Hsu, T., et al. (2007). *Mycobacterium tuberculosis* nuoG is a virulence gene that inhibits apoptosis of infected host cells. *PLoS Pathog*. **3**, e110. doi: 10.1371/journal.ppat.0030110.

Venugopal, A., Bryk, R., Shi, S., Rhee, K., Rath, P., Schnappinger, D., et al. (2011). Virulence of *Mycobacterium tuberculosis* depends on lipoamide dehydrogenase, a member of three multienzyme complexes. *Cell Host Microbe*. **9**, 21-31. doi: 10.1016/j.chom.2010.12.004.

Veyrier, F., Said-Salim, B., and Behr, M. A. (2008). Evolution of the mycobacterial SigK regulon. *J Bacteriol*. **190**, 1891-1899. doi: 10.1128/JB.01452-07.

Wang, C., Chen, Z., Fu, R., Zhang, Y., Chen, L., Huang, L., et al. (2011). A DNA vaccine expressing CFP21 and MPT64 fusion protein enhances BCG-induced protective immunity against *Mycobacterium tuberculosis* infection in mice. *Med Microbiol Immunol*. **200**, 165-175. doi: 10.1007/s00430-011-0188-z.

Wang, Q., Liu, S., Tang, Y., Liu, Q., and Yao, Y. (2014). MPT64 protein from *Mycobacterium tuberculosis* inhibits apoptosis of macrophages through NF-kB-miRNA21-Bcl-2 pathway. *PLoS One*. **9**, e100949. doi: 10.1371/journal.pone.0100949.

Wang, X., Wang, H., and Xie, J. (2011). Genes and regulatory networks involved in persistence of *Mycobacterium tuberculosis*. *Sci China Life Sci*. **54**, 300-310. doi: 10.1007/s11427-011-4134-5.

Wells, R. M., Jones, C. M., Xi, Z., Speer, A., Danilchanka, O., Doornbos, K. S., et al. (2013). Discovery of a siderophore export system essential for virulence of *Mycobacterium tuberculosis*. *PLoS Pathog*. **9**, e1003120. doi: 10.1371/journal.ppat.1003120.

Wilkinson, K. A., Stewart, G. R., Newton, S. M., Vordermeier, H. M., Wain, J. R., Murphy, H. N., et al. (2005). Infection biology of a novel alpha-crystallin of *Mycobacterium tuberculosis*: Acr2. *J Immunol*. **174**, 4237-4243.

Willemse, D., Weber, B., Masino, L., Warren, R. M., Adinolfi, S., Pastore, A., et al. (2018). Rv1460, a SufR homologue, is a repressor of the suf operon in *Mycobacterium tuberculosis*. *PLoS One*. **13**, e0200145. doi: 10.1371/journal.pone.0200145.

Williams, M. J., Kana, B. D., and Mizrahi, V. (2011). Functional analysis of molybdopterin biosynthesis in mycobacteria identifies a fused molybdopterin synthase in *Mycobacterium tuberculosis*. *J Bacteriol*. **193**, 98-106. doi: 10.1128/JB.00774-10.

Xue, T., Stavropoulos, E., Yang, M., Ragno, S., Vordermeier, M., Chambers, M., et al. (2004). RNA encoding the MPT83 antigen induces protective immune responses against *Mycobacterium tuberculosis* infection. *Infect Immun*. **72**, 6324-6329. doi: 10.1128/IAI.72.11.6324-6329.2004.

Yang, G., Luo, T., Sun, C., Yuan, J., Peng, X., Zhang, C., et al. (2017). PPE27 in *Mycobacterium smegmatis* enhances mycobacterial survival and manipulates cytokine secretion in mouse macrophages. *J Interferon Cytokine Res*. **37**, 421-431. doi: 10.1089/jir.2016.0126.

You, X., Li, R., Wan, K., Liu, L., Xie, X., Zhao, L., et al. (2017). Evaluation of Rv0220, Rv2958c, Rv2994 and Rv3347c of *Mycobacterium tuberculosis* for serodiagnosis of tuberculosis. *Microb Biotechnol*. **10**, 604-611. doi: 10.1111/1751-7915.12697.

Zhang, Z., Bulloch, E. M., Bunker, R. D., Baker, E. N., and Squire, C. J. (2009). Structure and function of GlmU from *Mycobacterium tuberculosis*. *Acta Crystallogr D Biol Crystallogr*. **65**, 275-283. doi: 10.1107/S0907444909001036.

Zheng, S., Zhou, Y., Fleming, J., Zhou, Y., Zhang, M., Li, S., et al. (2018). Structural and genetic analysis of START superfamily protein MSMEG_0129 from *Mycobacterium smegmatis*. *FEBS Lett*. **592**, 1445-1457. doi: 10.1002/1873-3468.13024.

Zhong, D., and Blount, P. (2013). Phosphatidylinositol is crucial for the mechanosensitivity of *Mycobacterium tuberculosis* MscL. *Biochemistry*. **52**, 5415-5420. doi: 10.1021/bi400790j.
