# Supplementary material for: Pan-Genomic Study of Mycobacterium tuberculosis Reflecting the Primary/Secondary Genes, Generality/Individuality, and the Interconversion Through Copy Number Variations
Source: Front Microbiol. 2018 Aug 17;9:1886. doi: 10.3389/fmicb.2018.01886 (PMC6109687; doi:10.3389/fmicb.2018.01886)
Supplement: Supplementary file 7 [file Table_7.DOCX]

Supplementary Table S7. Detailed information about the 28 Mtb SCGs.

| **SCG** | **Synonym** | **Average copy No.** | **Gene description** | **Function** | **Ref** |
| --- | --- | --- | --- | --- | --- |
| PE_PGRS10^a, c^ | Rv0747 | 26 | PE-PGRS family protein PE_PGRS10 |  |  |
| PE_PGRS15^a^ | Rv0872c | 2 | PE-PGRS family protein PE_PGRS15 |  |  |
| PE_PGRS18^a^ | Rv0980c | 3 | PE-PGRS family protein PE_PGRS18 | It is potentially required for endothelial-cell invasion / intracellular survival | (Yang et al., 2017) |
| PE_PGRS25^a^ | Rv1396c | 3 | PE-PGRS family protein PE_PGRS25 |  |  |
| PE_PGRS33^a, c^ | Rv1818c | 3 | PE-PGRS family protein PE_PGRS33 | It is Involved in virulence / Inactivation of PE_PGRS33 (Rv1818c) leads to loss of the aggregation, dispersed growth and reduced infection of macrophages | (Mukhopadhyay and Balaji., 2011; Akhter et al., 2012) |
| PPE19^a, c^ | Rv1361c | 2 | PPE family protein PPE19 | It contains a higher expression in macrophages / might be selectively induced in macrophages to facilitate intracellular survival of tubercle bacilli | (Dubnau and Smith 2003) |
| PPE38^a, c^ | Rv2352c | 2 | PPE family protein PPE38 | Can induce cellular immune response | (Meng et al., 2017) |
| PPE55^a, c^ | Rv3347c | 3 | PPE family protein PPE55 | It is a highly immunogenic protein that may be useful for differentiating between latent TB and incipient, subclinical TB | (Singh et al., 2005) |
| PPE66^a^ | Rv3738c | 3 | PPE family protein PPE66 | Closely linked to genes known to be important during infection and can affect the expression of adjacent genes | (Yesilkaya et al., 2006) |
| esxN^b, c^ | Rv1793 | 4 | ESAT-6 like protein EsxN |  |  |
| plcC^b^ | Rv2351c | 3 | membrane-associated phospholipase A | It plays a role in the pathogenesis | (Forrellad et al., 2013) |
| Rv3467^c^ | Rv3467 | 2 | hypothetical protein |  |  |
| Rv2512c^c, d^ | Rv2512c | 5 | insertion sequence element IS1081 transposase |  |  |
| Rv1041c^d^ | Rv1041c | 2 | IS2-like transposase |  |  |
| Rv1149^d^ | Rv1149 | 2 | transposase |  |  |
| Rv3844^d^ | Rv3844 | 2 | transposase |  |  |
| Rv3475^d^ | Rv3475 | 14 | insertion sequence element IS986/IS6110 transposase |  |  |
| Rv3474^d^ | Rv3474 | 12 | insertion sequence element IS6110 transposase(fragment) |  |  |
| vapB30 | Rv0623 | 2 | antitoxin VapB30 | It belongs to VapBC30 Toxin-antitoxin (TA) system, which is implicated in Mtb dormant state formation, virulence, and stress response. | (Lee et al., 2015) |
| fadD15 | Rv2187 | 2 | long-chain-fatty-acid--CoA ligase FadD15 | It is involved in fatty acid metabolism (NCBI) |  |
| moaE1 | Rv3119 | 2 | molybdopterin synthase catalytic subunit 1 | It is involved in molybdopterin synthesis, which is necessary for nitrate assimilatory | (Williams et al., 2011) |
| cysA3 | Rv3117 | 2 | thiosulfate sulfurtransferase | It plays a role in oxygen sensing | (Florczyk et al., 2001) |
| pks5 | Rv1527c | 2 | polyketide synthase | It is Involved in lipid metabolism | (Forrellad et al., 2013) |
| Rv2825c | Rv2825c | 2 | hypothetical protein |  |  |
| Rv2749 | Rv2749 | 2 | hypothetical protein |  |  |
| sseC1 | ERDMAN_3413 | 2 | hypothetical protein |  |  |
| Rv1148c | Rv1148c | 2 | hypothetical protein |  |  |
| ERDMAN_1749 | ERDMAN_1749 | 2 | hypothetical protein |  |  |

^a^ PE/PPE genes; ^b^ Virulence genes; ^c^ Antigen genes; ^d^ Transposase genes.

**References**

Akhter, Y., Ehebauer, M. T., Mukhopadhyay, S., and Hasnain, S. E. (2012). The *PE/PPE* multigene family codes for virulence factors and is a possible source of mycobacterial antigenic variation: Perhaps more? *Biochimie*. 94, 110-116. doi: 10.1016/j.biochi.2011.09.026.

Dubnau, E., and Smith, I. (2003). *Mycobacterium tuberculosis* gene expression in macrophages. *Microbes. Infect*. 5, 629-637. doi: 10.1016/S1286-4579(03)00090-X.

Florczyk, M. A., McCue, L. A., Stack, R. F., Hauer, C. R., and McDonough, K. A. (2001). Identification and characterization of mycobacterial proteins differentially expressed under standing and shaking culture conditions, including Rv2623 from a novel class of putative atp-binding proteins. *Infect. Immun*. 69, 5777-5785. doi: 10.1128/iai.69.9.5777-5785.2001.

Forrellad, M. A., Klepp, L. I., Gioffre, A., Garcia, J. S. Y., Morbidoni, H. R., Santangelo, M. D., et al. (2013). Virulence factors of the *Mycobacterium tuberculosis* complex. *Virulence*. 4, 3-66. doi: 10.4161/viru.22329.

Lee, I. G., Lee, S. J., Chae, S., Lee, K. Y., Kim, J. H., and Lee, B. J. (2015). Structural and functional studies of the *Mycobacterium tuberculosis* VapBC30 toxin-antitoxin system: Implications for the design of novel antimicrobial peptides. *Nucleic. Acids. Res*. 43, 7624-7637. doi: 10.1093/nar/gkv689.

Meng, L., Tong, J., Wang, H., Tao, C., Wang, Q., Niu, C., et al. (2017). PPE38 protein of *Mycobacterium tuberculosis* inhibits macrophage MHC class I expression and dampens CD8+ T cell responses. *Front. Cell. Infect. Microbiol*. 7, 68. doi: 10.3389/fcimb.2017.00068.

Mukhopadhyay, S., and Balaji, K. N. (2011). The PE and PPE proteins of *Mycobacterium tuberculosis*. *Tuberculosis*. 91, 441-447. doi: 10.1016/j.tube.2011.04.004.

Singh, K. K., Dong, Y., Patibandla, S. A., McMurray, D. N., Arora, V. K., and Laal, S. (2005). Immunogenicity of the *Mycobacterium tuberculosis* PPE55 (rv3347c) protein during incipient and clinical tuberculosis. *Infect. Immun*. 73, 5004-5014. doi: 10.1128/IAI.73.8.5004-5014.2005.

Williams, M. J., Kana, B. D., and Mizrahi, V. (2011). Functional analysis of molybdopterin biosynthesis in mycobacteria identifies a fused molybdopterin synthase in *Mycobacterium tuberculosis*. *J. Bacteriol*. 193, 98-106. doi: 10.1128/JB.00774-10.

Yang, W., Deng, W., Zeng, J., Ren, S., Ali, M. K., Gu, Y., et al. (2017). *Mycobacterium tuberculosis* PE_PGRS18 enhances the intracellular survival of *M. smegmatis* via altering host macrophage cytokine profiling and attenuating the cell apoptosis. *Apoptosis*. 22, 502-509. doi: 10.1007/s10495-016-1336-0.

Yesilkaya, H., Forbes, K. J., Shafi, J., Smith, R., Dale, J. W., Rajakumar, K., et al. (2006). The genetic portrait of an outbreak strain. *Tuberculosis*. 86, 357-362. doi: 10.1016/j.tube.2005.08.019.
