# Supplementary material for: Pan-Genomic Study of Mycobacterium tuberculosis Reflecting the Primary/Secondary Genes, Generality/Individuality, and the Interconversion Through Copy Number Variations
Source: Front Microbiol. 2018 Aug 17;9:1886. doi: 10.3389/fmicb.2018.01886 (PMC6109687; doi:10.3389/fmicb.2018.01886)
Supplement: Supplementary file 8 [file Table_8.DOCX]

**Supplementary Table S8.** Transcriptome expression data of the SCGs in the exponential and stationary phases^a^

| **SCG** | **Copy** | **Gene** | **Exponential phase, biological replicate 1** | | **Exponential phase, biological replicate 2** | | **Stationary phase, biological replicate 1** | | **Stationary phase, biological replicate 2** | |
| --- | --- | --- | --- | --- | --- | --- | --- | --- | --- | --- |
|  |  |  | **Expression value** | **TPM** | **Expression value** | **TPM** | **Expression value** | **TPM** | **Expression value** | **TPM** |
| PE_PGRS10 | Copy1 | Rv0747 | 2,818 | 35.9 | 2,941 | 39.6 | 2,325 | 32.7 | 3,576 | 37.1 |
| PE_PGRS10 | Copy2 | Rv0833 | 2,037 | 27.8 | 2,479 | 35.7 | 1,075 | 16.2 | 1,738 | 19.3 |
| PE_PGRS10 | Copy3 | Rv0278c | 866 | 9.2 | 966 | 10.9 | 766 | 9.0 | 1,115 | 9.7 |
| PE_PGRS10 | Copy4 | Rv0279c | 3,769 | 46.0 | 4,319 | 55.7 | 1,284 | 17.3 | 2,013 | 20.0 |
| PE_PGRS10 | Copy5 | Rv1759c | 672 | 7.5 | 651 | 7.7 | 3,001 | 37.0 | 4,658 | 42.4 |
| PE_PGRS10 | Copy6 | Rv0746 | 1,074 | 14.0 | 1,234 | 17.0 | 319 | 4.6 | 478 | 5.1 |
| PE_PGRS10 | Copy7 | Rv2162c | 16,562 | 317.7 | 17,438 | 353.4 | 12,757 | 270.2 | 19,548 | 305.4 |
| PE_PGRS10 | Copy8 | Rv1067c | 301 | 4.6 | 313 | 5.1 | 223 | 3.8 | 358 | 4.5 |
| PE_PGRS10 | Copy9 | Rv1087 | 2,740 | 36.5 | 2,705 | 38.0 | 3,675 | 54.0 | 5,702 | 61.8 |
| PE_PGRS10 | Copy10 | Rv1091 | 3,117 | 37.3 | 3,459 | 43.7 | 1,676 | 22.2 | 2,527 | 24.6 |
| PE_PGRS10 | Copy11 | Rv1068c | 247 | 5.4 | 279 | 6.5 | 73 | 1.8 | 122 | 2.2 |
| PE_PGRS10 | Copy12 | Rv1243c | 341 | 6.2 | 326 | 6.3 | 2,419 | 48.5 | 3,597 | 53.2 |
| PE_PGRS10 | Copy13 | Rv1452c | 302 | 4.2 | 336 | 4.9 | 228 | 3.5 | 372 | 4.2 |
| PE_PGRS10 | Copy14 | Rv0834c | 6,116 | 70.8 | 6,291 | 76.9 | 24,177 | 309.1 | 38,242 | 360.6 |
| PE_PGRS10 | Copy15 | Rv3511 | 1,433 | 20.5 | 1,620 | 24.5 | 1,171 | 18.5 | 2,044 | 23.8 |
| PE_PGRS10 | Copy16 | Rv3345c | 663 | 4.4 | 736 | 5.2 | 3,848 | 28.2 | 5,608 | 30.3 |
| PE_PGRS10 | Copy17 | Rv3508 | 15,196 | 81.7 | 17,198 | 97.7 | 2,788 | 16.5 | 4,242 | 18.6 |
| PE_PGRS10 | Copy18 | Rv3514 | 6,563 | 45.0 | 7,438 | 53.9 | 5,320 | 40.3 | 8,680 | 48.5 |
| PE_PGRS10 | Copy19 | Rv3388 | 893 | 12.5 | 949 | 14.0 | 221 | 3.4 | 369 | 4.2 |
| PE_PGRS10 | Copy20 | Rv1450c | 515 | 4.0 | 630 | 5.1 | 1,937 | 16.4 | 2,923 | 18.3 |
| PE_PGRS10 | Copy21 | Rv2490c | 345 | 2.1 | 437 | 2.8 | 645 | 4.4 | 1,000 | 5.0 |
| PE_PGRS10 | Copy22 | Rv3367 | 1,852 | 32.1 | 1,865 | 34.2 | 1,727 | 33.1 | 2,583 | 36.5 |
| PE_PGRS10 | Copy23 | Rv0578c | 12,199 | 95.4 | 11,701 | 96.7 | 16,834 | 145.4 | 24,271 | 154.6 |
| PE_PGRS10 | Copy24 | Rv3507 | 3,553 | 26.3 | 3,531 | 27.6 | 1,987 | 16.2 | 3,121 | 18.8 |
| Rv1148c | Copy1 | Rv1148c | 1,167 | 24.7 | 1,150 | 25.7 | 802 | 18.7 | 1,232 | 21.2 |
| Rv1148c | Copy2 | Rv1945 | 542 | 12.2 | 562 | 13.3 | 264 | 6.5 | 305 | 5.6 |
| moaE1 | Copy1 | Rv3119 | 1,227 | 84.8 | 1,161 | 84.7 | 141 | 10.8 | 150 | 8.4 |
| moaE1 | Copy2 | Rv3323c | 555 | 25.6 | 481 | 23.4 | 252 | 12.8 | 360 | 13.5 |
| esxN | Copy1 | Rv1793 | 3,840 | 413.2 | 3,814 | 433.6 | 1,722 | 204.6 | 2,296 | 201.2 |
| esxN | Copy2 | Rv3619c | 86 | 9.3 | 108 | 12.3 | 64 | 7.6 | 114 | 10.0 |
| esxN | Copy3 | Rv1198 | 8,678 | 933.9 | 7,745 | 880.5 | 137 | 16.3 | 168 | 14.7 |
| esxN | Copy4 | Rv2346c | 5,430 | 584.3 | 5,420 | 616.2 | 541 | 64.3 | 898 | 78.7 |
| fadD15 | Copy1 | Rv2187 | 2,996 | 51.0 | 2,968 | 53.3 | 282 | 5.3 | 402 | 5.6 |
| fadD15 | Copy2 | Rv1550 | 884 | 15.8 | 773 | 14.6 | 187 | 3.7 | 239 | 3.5 |
| plcA | Copy1 | Rv2351c | 587 | 11.7 | 602 | 12.7 | 94 | 2.1 | 114 | 1.9 |
| plcA | Copy2 | Rv2349c | 1,678 | 33.7 | 1,661 | 35.2 | 93 | 2.1 | 126 | 2.1 |
| plcA | Copy3 | Rv2350c | 1,873 | 37.3 | 1,721 | 36.2 | 122 | 2.7 | 148 | 2.4 |
| PPE55 | Copy1 | Rv3347c | 5,994 | 19.4 | 5,393 | 18.4 | 14,021 | 50.1 | 20,309 | 53.5 |
| PPE55 | Copy2 | Rv3350c | 2,091 | 5.8 | 1,951 | 5.7 | 2,083 | 6.3 | 3,230 | 7.2 |
| vapB30 | Copy1 | Rv0623 | 587 | 70.6 | 650 | 82.6 | 1,508 | 200.3 | 1,987 | 194.6 |
| vapB30 | Copy2 | Rv1740 | 346 | 49.8 | 335 | 51.0 | 330 | 52.5 | 366 | 42.9 |
| PE_PGRS15 | Copy1 | Rv0872c | 8,076 | 136.0 | 8,998 | 160.1 | 5,778 | 107.4 | 8,485 | 116.4 |
| PE_PGRS15 | Copy2 | Rv2853 | 798 | 13.2 | 792 | 13.9 | 381 | 7.0 | 555 | 7.5 |
| pks5 | Copy1 | Rv1527c | 3,797 | 18.4 | 3,513 | 18.0 | 2,142 | 11.5 | 3,157 | 12.5 |
| pks5 | Copy2 | Rv3825c | 64,417 | 309.6 | 58,983 | 299.5 | 27,729 | 147.2 | 31,137 | 121.9 |
| PPE66 | Copy1 | Rv3738c | 310 | 10.0 | 284 | 9.7 | 449 | 16.0 | 693 | 18.3 |
| PPE66 | Copy2 | Rv3018c | 107 | 2.5 | 138 | 3.4 | 170 | 4.4 | 297 | 5.7 |
| PPE66 | Copy3 | Rv0256c | 1,088 | 20.0 | 1,119 | 21.7 | 864 | 17.5 | 1,256 | 18.8 |
| PE_PGRS25 | Copy1 | Rv1396c | 12,188 | 215.9 | 11,166 | 209.0 | 20,173 | 394.6 | 30,754 | 443.8 |
| PE_PGRS25 | Copy2 | Rv0124 | 210 | 4.4 | 226 | 5.0 | 339 | 7.8 | 508 | 8.7 |
| PE_PGRS25 | Copy3 | Rv0532 | 919 | 15.8 | 1,096 | 19.9 | 193 | 3.7 | 331 | 4.6 |
| PPE38 | Copy1 | Rv2352c | 301 | 7.9 | 287 | 7.9 | 135 | 3.9 | 192 | 4.1 |
| PPE38 | Copy2 | Rv3125c | 257 | 6.7 | 222 | 6.1 | 71 | 2.0 | 121 | 2.6 |
| PPE19 | Copy1 | Rv1361c | 12,579 | 323.9 | 13,128 | 357.2 | 445 | 12.7 | 721 | 15.1 |
| PPE19 | Copy2 | Rv3478 | 34,614 | 898.1 | 34,745 | 952.4 | 1,863 | 53.4 | 2,721 | 57.5 |
| sseC1 | Copy1 | Rv3118 | 2,041 | 206.6 | 2,527 | 270.2 | 271 | 30.3 | 486 | 40.1 |
| sseC1 | Copy2 | Rv0814c | 586 | 59.3 | 162 | 17.3 | 120 | 13.4 | 76 | 6.3 |
| cysA3 | Copy1 | Rv3117 | 2,061 | 75.8 | 1,779 | 69.1 | 277 | 11.2 | 355 | 10.6 |
| cysA3 | Copy2 | Rv0815c | 2,187 | 80.4 | 1,851 | 71.9 | 272 | 11.0 | 341 | 10.2 |
| Rv2825c | Copy1 | Rv2825c | 2,768 | 131.0 | 3,080 | 154.0 | 1,931 | 100.9 | 2,635 | 101.6 |
| Rv2825c | Copy2 | Rv2828c | 525 | 29.5 | 567 | 33.6 | 210 | 13.0 | 277 | 12.7 |
| Rv3467 | Copy1 | Rv3467 | 26 | 0.8 | 47 | 1.6 | 67 | 2.4 | 121 | 3.2 |
| Rv3467 | Copy2 | Rv0094c | 34 | 1.1 | 41 | 1.4 | 81 | 2.9 | 106 | 2.8 |
| Rv2749 | Copy1 | Rv2749 | 748 | 72.8 | 741 | 76.2 | 359 | 38.6 | 434 | 34.4 |
| Rv2749 | Copy2 | Rv0793 | 23 | 2.3 | 22 | 2.3 | 84 | 9.3 | 101 | 8.2 |
| Rv2512c | Copy1 | Rv2512c | 160 | 3.9 | 158 | 4.1 | 760 | 20.6 | 1,090 | 21.8 |
| Rv2512c | Copy2 | Rv1199c | 161 | 4.0 | 163 | 4.2 | 782 | 21.2 | 1,124 | 22.5 |
| Rv2512c | Copy3 | Rv3115 | 67 | 1.6 | 68 | 1.8 | 923 | 25.0 | 1,297 | 26.0 |
| Rv2512c | Copy4 | Rv3023c | 86 | 2.1 | 55 | 1.4 | 951 | 25.8 | 1,319 | 26.4 |
| Rv2512c | Copy5 | Rv1047 | 94 | 2.3 | 68 | 1.8 | 874 | 23.7 | 1,328 | 26.6 |
| PE_PGRS18 | Copy1 | Rv0980c | 93 | 2.1 | 91 | 2.1 | 444 | 10.9 | 629 | 11.4 |
| PE_PGRS18 | Copy2 | Rv0978c | 44 | 1.4 | 27 | 0.9 | 152 | 5.2 | 218 | 5.5 |
| PE_PGRS18 | Copy3 | Rv2615c | 1,681 | 37.2 | 2,011 | 47.0 | 962 | 23.5 | 1,455 | 26.2 |
| Rv1041c | Copy1 | Rv1041c | 1,508 | 53.5 | 1,434 | 53.8 | 1,867 | 73.2 | 2,515 | 72.7 |
| Rv1041c | Copy2 | Rv1041c | 1,508 | 53.5 | 1,434 | 53.8 | 1,867 | 73.2 | 2,515 | 72.7 |
| Rv1149 | Copy1 | Rv1149 | 1,432 | 107.6 | 1,564 | 124.2 | 754 | 62.6 | 935 | 57.2 |
| Rv3844 | Copy1 | Rv3844 | 336 | 20.9 | 348 | 22.9 | 1,105 | 76.1 | 1,402 | 71.2 |
| Rv3844 | Copy2 | Rv3348 | 342 | 21.3 | 306 | 20.2 | 1,045 | 71.9 | 1,436 | 72.9 |
| PE_PGRS33 | Copy1 | Rv1818c | 3,820 | 78.3 | 4,317 | 93.4 | 1,222 | 27.6 | 1,849 | 30.9 |
| PE_PGRS33 | Copy2 | Rv2741 | 1,749 | 34.0 | 1,930 | 39.6 | 586 | 12.6 | 843 | 13.3 |
| PE_PGRS33 | Copy3 | Rv0832 | 1,175 | 87.0 | 1,241 | 97.1 | 385 | 31.5 | 491 | 29.6 |

*TPM: Transcripts Per Million.

^a^The above data was downloaded from NCBI GSE100097 (Aguilar-Ayala, D. A., Tilleman, L., Van Nieuwerburgh, F., Deforce, D., Palomino, J. C., Vandamme, P., et al. (2017). The transcriptome of *Mycobacterium tuberculosis* in a lipid-rich dormancy model through rnaseq analysis. *Sci. Rep*. **7**, 17665. doi: 10.1038/s41598-017-17751-x).
