# Supplementary material for: Pan-Genomic Study of Mycobacterium tuberculosis Reflecting the Primary/Secondary Genes, Generality/Individuality, and the Interconversion Through Copy Number Variations
Source: Front Microbiol. 2018 Aug 17;9:1886. doi: 10.3389/fmicb.2018.01886 (PMC6109687; doi:10.3389/fmicb.2018.01886)
Supplement: Supplementary file 9 [file Table_9.DOCX]

Supplementary Table S9. Average copy number of the 28 SCGs in Mtb, Mbo, and STB strains.

| **Gene** | **Mtb** | **Mbo** | **STB** |
| --- | --- | --- | --- |
| PE_PGRS10^a, c^ | 26 | 12 | 20 |
| Rv3475^d^ | 14 | 1 | 3 |
| Rv3474^d^ | 12 | 1 | 4 |
| Rv2512c^c, d^ | 5 | 4 | 2 |
| esxN^b, c^ | 4 | 1 | 5 |
| PE_PGRS18^a^ | 31 | 1 | 3 |
| plcA | 3 | 1 | 4 |
| PPE66 ^a^ | 3 | 1 | 2 |
| PE_PGRS25 ^a^ | 3 | 1 | 5 |
| PE_PGRS33 ^a, c^ | 3 | 1 | 4 |
| PPE55^a, c^ | 3 | 1 | 1 |
| Rv3467^c^ | 2 | 2 | 5 |
| ERDMAN_1749 | 2 | 3 | 0 |
| PPE38 ^a, c^ | 2 | 1 | 2 |
| Rv1041c^d^ | 2 | 2 | 1 |
| PE_PGRS15^a^ | 2 | 1 | 1 |
| Rv1149^d^ | 2 | 2 | 1 |
| vapB30 | 2 | 1 | 2 |
| Rv1148c | 2 | 1 | 3 |
| fadD15 | 2 | 1 | 2 |
| Rv2825c | 2 | 1 | 1 |
| pks5 | 2 | 1 | 4 |
| Rv2749 | 2 | 1 | 2 |
| PPE19 | 2 | 1 | 3 |
| sseC1 | 2 | 1 | 1 |
| moaE1 | 2 | 1 | 1 |
| cysA3 | 2 | 2 | 1 |
| Rv3844 ^d^ | 2 | 2 | 1 |

^a^ PE/PPE genes; ^b^ Virulence genes; ^c^ Antigen genes; ^d^ Transposase genes.
