# Supplementary material for: Pan-Genomic Study of Mycobacterium tuberculosis Reflecting the Primary/Secondary Genes, Generality/Individuality, and the Interconversion Through Copy Number Variations
Source: Front Microbiol. 2018 Aug 17;9:1886. doi: 10.3389/fmicb.2018.01886 (PMC6109687; doi:10.3389/fmicb.2018.01886)
Supplement: Supplementary file 10 [file Table_10.DOCX]

Supplementary Table S10. Detailed information about the 48 Mtb-specific single-copy core genes.

| **Gene** | **Synonym** | **Product length** | **COG** | **Annotation** | **RD No.** |
| --- | --- | --- | --- | --- | --- |
| Rv0024 | Rv0024 | 281 | COG0791M | NLP/P60 family protein |  |
| Rv0221^b^ | Rv0221 | 469 | COG4908R | diacyglycerol O-acyltransferase | RD10 |
| echA1^b^ | Rv0222 | 262 | COG1024I | enoyl-CoA hydratase EchA1 | RD10 |
| pknD^b^ | Rv0931c | 664 | COG0515RTKL | serine/threonine-protein kinase PknD |  |
| PPE17 | Rv1168c | 346 | COG5651N | PPE family protein PPE17 |  |
| Rv1255c^b^ | Rv1255c | 202 | COG1309K | HTH-type transcriptional regulator | RD13 |
| cyp130^b^ | Rv1256c | 405 | COG2124Q | cytochrome P450 Cyp130 | RD13 |
| Rv1257c | Rv1257c | 455 | COG0277C | oxidoreductase | RD13 |
| Rv1507c | Rv1507c | 231 | - | hypothetical protein | RD4 |
| Rv1508c | Rv1508c | 599 | - | membrane protein | RD4 |
| Rv1509 | Rv1509 | 293 | COG2227H | hypothetical protein | RD4 |
| Rv1510 | Rv1510 | 432 | COG2244R | hypothetical protein | RD4 |
| gmdA | Rv1511 | 340 | COG1089M | GDP-D-mannose dehydratase GmdA | RD4 |
| epiA | Rv1512 | 322 | COG0451MG | nucleotide-sugar epimerase EpiA | RD4 |
| Rv1513^b^ | Rv1513 | 243 | - | hypothetical protein | RD4 |
| Rv1514c | Rv1514c | 262 | COG0463M | glycosyltransferase | RD4 |
| Rv1515c | Rv1515c | 298 | COG2227H | hypothetical protein | RD4 |
| Rv1516c | Rv1516c | 336 | COG0463M | sugar transferase | RD4 |
| gnd1b | Rv1844c | 485 | COG0362G | 6-phosphogluconate dehydrogenase |  |
| yrbE3B^b^ | Rv1965 | 271 | COG0767Q | integral membrane protein | RD7 |
| mce3A^a^ | Rv1966 | 425 | COG1463Q | Mce family protein Mce3A | RD7 |
| mce3B^a^ | Rv1967 | 342 | COG1463Q | Mce family protein Mce3B | RD7 |
| mce3C^a^ | Rv1968 | 410 | COG1463Q | Mce family protein Mce3C | RD7 |
| mce3Da | Rv1969 | 423 | COG1463Q | Mce family protein Mce3D | RD7 |
| lprM^a^ | Rv1970 | 377 | COG1463Q | Mce family lipoprotein LprM | RD7 |
| mce3F^a^ | Rv1971 | 437 | COG1463Q | Mce family protein Mce3F | RD7 |
| Rv1972 | Rv1972 | 191 | - | Mce associated membrane protein | RD7 |
| Rv1973^a^ | Rv1973 | 160 | - | Mce associated membrane protein | RD7 |
| Rv1974 | Rv1974 | 125 | - | membrane protein | RD7 |
| Rv1975 | Rv1975 | 221 | COG2340S | hypothetical protein | RD7 |
| Rv1976^c^ | Rv1976c | 135 | - | hypothetical protein | RD7 |
| Rv1977^b^ | Rv1977 | 348 | COG0501O | hypothetical protein | RD7 |
| Rv2073c | Rv2073c | 249 | COG0300R | oxidoreductase |  |
| Rv2074^b^ | Rv2074 | 137 | - | pyridoxamine 5'-phosphate oxidase | RD9 |
| Rv2348c | Rv2348c | 108 | - | hypothetical protein | RD5 |
| Rv2645 | Rv2645 | 143 | - | hypothetical protein |  |
| Rv2646 | Rv2646 | 332 | COG4974L | integrase |  |
| Rv2654c^b^ | Rv2654c | 81 | - | antitoxin | RD11 (phiRv2) |
| Rv2655c | Rv2655c | 475 | - | prophage protein | RD11 (phiRv2) |
| Rv2657c | Rv2657c | 86 | - | prophage protein | RD11 (phiRv2) |
| Rv2658c | Rv2658c | 120 | - | prophage protein | RD11 (phiRv2) |
| Rv2659c | Rv2659c | 375 | - | prophage protein | RD11 (phiRv2) |
| cyp141 | Rv3121 | 400 | COG2124Q | cytochrome P450 Cyp141 |  |
| ephA^b^ | Rv3617 | 322 | COG0596R | epoxide hydrolase EphA | RD8 |
| Rv3618 | Rv3618 | 395 | COG2141C | monooxygenase | RD8 |
| PPE65^b^ | Rv3621c | 413 | COG5651N | PPE family protein PPE65 | RD8 |
| PE32^b^ | Rv3622c | 99 | - | PE family protein PE32 | RD8 |
| Rv3888c^a^ | Rv3888c | 341 | COG0455D | membrane protein |  |

^a^ PE/PPE genes; ^b^ Virulence genes; ^c^ Antigen genes.
