# Supplementary material for: Pan-Genomic Study of Mycobacterium tuberculosis Reflecting the Primary/Secondary Genes, Generality/Individuality, and the Interconversion Through Copy Number Variations
Source: Front Microbiol. 2018 Aug 17;9:1886. doi: 10.3389/fmicb.2018.01886 (PMC6109687; doi:10.3389/fmicb.2018.01886)
Supplement: Supplementary file 11 [file Table_11.DOCX]

Supplementary Table S11. Detailed information about the 19 Mbo-specific single-copy core genes.

| **Gene** | **Synonym** | **Product length** | **COG** | **Annotation** | **Gene** |
| --- | --- | --- | --- | --- | --- |
| Mb0227 | Mb0227 | 257 | - | hypothetical protein | Mb0227 |
| Mb0807 | Mb0807 | 191 | COG3573R | hypothetical protein | Mb0807 |
| Mb0818c | Mb0818c | 203 | COG1249C | oxidoreductase | Mb0818c |
| pknDb | Mb0954c | 369 | COG3391S | serine/threonine protein kinase D | pknDb |
| pknDa | Mb0955c | 291 | COG0515RTKL | serine/threonine protein kinase D | pknDa |
| pstBa | Mb0957 | 71 | COG1117P | phosphate ABC transporter | pstBa |
| Mb1287c | Mb1287c | 99 | - | hypothetical protein | Mb1287c |
| Mb1291 | Mb1291 | 102 | COG0654HC | oxidoreductase | Mb1291 |
| Mb1928 | Mb1928 | 107 | COG1064R | dehydrogenase | Mb1928 |
| cobLa | Mb2099c | 62 | COG2241H | precorrin-6y methyltransferase | cobLa |
| Mb2307c | Mb2307c | 87 | - | hypothetical protein | Mb2307c |
| Mb2448c | Mb2448c | 97 | - | transposase | Mb2448c |
| Mb2595 | Mb2595 | 533 | COG4196S | hypothetical protein | Mb2595 |
| Mb2770 | Mb2770 | 104 | COG1359S | hypothetical protein | Mb2770 |
| Mb2784c | Mb2784c | 43 | - | hypothetical protein | Mb2784c |
| mesTb | Mb3201c | 105 | - | epoxide hydrolase | mesTb |
| PE_PGRS50a | Mb3377c | 539 | - | hypothetical protein | PE_PGRS50a |
| PPE55a | Mb3380c | 2096 | COG5651N | hypothetical protein | PPE55a |
| cyp142b | Mb3547c | 193 | COG2124Q | cytochrome P450 monooxygenase | cyp142b |
