# Supplementary material for: Pan-Genomic Study of Mycobacterium tuberculosis Reflecting the Primary/Secondary Genes, Generality/Individuality, and the Interconversion Through Copy Number Variations
Source: Front Microbiol. 2018 Aug 17;9:1886. doi: 10.3389/fmicb.2018.01886 (PMC6109687; doi:10.3389/fmicb.2018.01886)
Supplement: Supplementary file 12 [file Table_12.DOCX]

Supplementary Table S12. Detailed information about the L2/L4-specific single-copy core genes.

|  | **Gene** | **Product length** | **Annotation** |
| --- | --- | --- | --- |
| L2 specific genes | dosTa | 301 | Partial of two component sensor histidine kinase DosT |
|  | dosTb | 255 | Partial of two component sensor histidine kinase DosT |
|  | PPE57a | 83 | Partial of PPE family protein PPE57 |
|  | RN10_0073 | 47 | hypothetical protein |
|  | RN10_0122 | 60 | hypothetical protein |
| L4 specific genes | mmpL13a | 303 | Partial of transmembrane transport protein MmpL13 |
|  | Rv0325 | 74 | hypothetical protein |
|  | Rv2292c | 74 | hypothetical protein |
