# Supplementary material for: Pan-Genomic Study of Mycobacterium tuberculosis Reflecting the Primary/Secondary Genes, Generality/Individuality, and the Interconversion Through Copy Number Variations
Source: Front Microbiol. 2018 Aug 17;9:1886. doi: 10.3389/fmicb.2018.01886 (PMC6109687; doi:10.3389/fmicb.2018.01886)
Supplement: Supplementary file 13 [file Table_13.DOCX]

**Supplementary Table S13.** Functional analysis of specific genes in the Mtb strains.

| **Gene_ID** | **COG** | **Annotation** |
| --- | --- | --- |
| Erdman-ATCC-35801-NC_020559_prot_01235 | COG5164K | PE-PGRS family protein PE_PGRS22 |
| 2242_prot_03769 | COG5651N | PPE family protein PPE54 |
| 2242_prot_02010 | COG0342U | Probable protein-export membrane protein SecD |
| 2242_prot_02791 | COG5651N | PPE family protein PPE34 |
| 2242_prot_01232 | COG4907S | PE-PGRS family protein PE_PGRS21 |
| 2242_prot_00227 | COG2197TK | Possible two component transcriptional regulatory protein (probably LuxR-family) |
| 2242_prot_03768 | COG5651N | PPE family protein PPE54 |
| 2279_prot_02132 | COG5651N | PPE family protein PPE34 |
| 2279_prot_00057 | COG0596R | Possible hydrolase |
| 2279_prot_03961 | COG0318IQ | Probable fatty-acid-CoA ligase FadD18 (fragment) (fatty-acid-CoA synthetase) (fatty-acid-CoA synthase) |
| 2279_prot_03676 | COG2897P | Probable thiosulfate sulfurtransferase SseA (rhodanese) (thiosulfate cyanide transsulfurase) (thiosulfate thiotransferase) |
| 2279_prot_02466 | COG0723C | Probable rieske iron-sulfur protein QcrA |
| 2279_prot_03165 | COG4293S | Conserved hypothetical protein |
| 2279_prot_03383 | COG3328L | Probable transposase |
| 2279_prot_01493 | COG0296G | 1,4-alpha-glucan branching enzyme GlgB (glycogen branching enzyme) |
| 7199-99-NC_020089_prot_00566 | COG2409R | Probable conserved transmembrane transport protein MmpL2 |
| 7199-99-NC_020089_prot_02518 | COG0306P | Putative phosphate-transport permease PitB |
| 7199-99-NC_020089_prot_02883 | COG4262R | Probable spermidine synthase SpeE (putrescine aminopropyltransferase) (aminopropyltransferase) (SPDSY) |
| 22103_prot_02977 | COG1055P | Probable arsenic-transport integral membrane protein ArsB1 |
| 22103_prot_01888 | COG3246S | Conserved hypothetical protein |
| 22103_prot_03817 | COG1674D | ESX conserved component EccC4. ESX-4 type VII secretion system protein. Probable membrane protein. |
| 22103_prot_03840 | COG0028EH | Probable acetolactate synthase (large subunit) IlvB2 (AHAS) (acetohydroxy-acid synthase large subunit) (ALS) |
| 22103_prot_03137 | COG0534V | Possible DNA-damage-inducible protein F DinF |
| 22103_prot_00984 | COG3021S | Probable exported protein |
| 22103_prot_00086 | COG1136V | Probable glutamine-transport ATP-binding protein ABC transporter |
| 22103_prot_03258 | COG4584L | Probable transposase for insertion sequence element IS1533 |
| 22103_prot_00087 | COG1136V | Probable glutamine-transport ATP-binding protein ABC transporter |
| 22103_prot_00726 | COG3670Q | Probable dioxygenase |
| 22103_prot_00635 | COG3324R | Conserved protein TB27.3 |
| 22103_prot_00090 | COG1168E | Probable aminotransferase |
| 22115_prot_01342 | COG0596R | Unknown protein |
| 22115_prot_00436 | COG1252C | Probable membrane NADH dehydrogenase NdhA |
| 22115_prot_01566 | COG5164K | PE-PGRS family protein PE_PGRS28 |
| 22115_prot_00435 | COG1252C | Probable membrane NADH dehydrogenase NdhA |
| 22115_prot_01486 | COG2114T | Possible adenylate cyclase (ATP pyrophosphate-lyase) (adenylyl cyclase) |
| 22115_prot_03874 | COG5651N | PE family protein PPE60 |
| 22115_prot_00215 | COG1472G | Probable beta-glucosidase BglS (gentiobiase) (cellobiase) (beta-D-glucoside glucohydrolase) |
| 22115_prot_02621 | COG3511M | Probable phospholipase C 3 PlcC |
| 22115_prot_01769 | COG4653R | Probable PhiRv1 phage protein |
| 22115_prot_00906 | COG2897P | Probable thiosulfate sulfurtransferase CysA3 (rhodanese-like protein) (thiosulfate cyanide transsulfurase) (thiosulfate thiotr> |
| 22115_prot_02631 | COG5651N | PPE family protein PPE38 |
| 22115_prot_00642 | COG3832S | Probable transcriptional regulatory protein (possibly ArsR-family) |
| 22115_prot_03751 | COG1357S | Conserved protein |
| 22115_prot_00905 | COG2897P | Probable thiosulfate sulfurtransferase CysA3 (rhodanese-like protein) (thiosulfate cyanide transsulfurase) (thiosulfate thiotr> |
| 22115_prot_01768 | COG4653R | Probable PhiRv1 phage protein |
| 26105_prot_01315 | COG1051F | Probable mutator protein MutT2 (7,8-dihydro-8-oxoguanine-triphosphatase) (8-oxo-dGTPase) |
| 26105_prot_01412 | COG2814G | Probable drug-transport integral membrane protein |
| 26105_prot_04177 | COG1964R | Possible transferase |
| 26105_prot_03697 | COG3119P | Probable arylsulfatase AtsB (aryl-sulfate sulphohydrolase) (sulfatase) |
| 26105_prot_02331 | COG1429H | Cobalamin biosynthesis protein CobN |
| 26105_prot_03945 | COG1024I | Possible enoyl-CoA hydratase EchA19 (enoyl hydrase) (unsaturated acyl-CoA hydratase) (crotonase) |
| 26105_prot_04097 | COG4962U | Conserved hypothetical protein |
| 26105_prot_02359 | COG5665K | Conserved hypothetical protein |
| 26105_prot_01923 | COG1878R | Conserved hypothetical protein |
| 26105_prot_01020 | COG3670Q | Possible dioxygenase |
| 26105_prot_01865 | COG3321Q | Probable polyketide synthase Pks8 |
| 26105_prot_00164 | COG3315Q | Possible S-adenosylmethionine-dependent methyltransferase |
| 26105_prot_02654 | COG3511M | Membrane-associated phospholipase C 1 PlcA (MTP40 antigen) |
| 37004_prot_02058 | COG3064M | PE-PGRS family protein PE_PGRS34 |
| 37004_prot_03281 | COG3321Q | Probable polyketide synthase Pks1 |
| 37004_prot_01535 | COG3903R | Probable transcriptional regulatory protein |
| 37004_prot_04267 | COG2452L | Possible resolvase |
| 37004_prot_00508 | COG3564S | Conserved hypothetical protein |
| 37004_prot_02633 | COG5651N | PPE family protein PPE38 |
| 37004_prot_00139 | COG0583K | Oxidative stress response regulatory protein OxyS |
| 37004_prot_01696 | COG0463M | Probable glycosyltransferase |
| 96075-CP009426_prot_00096 | COG0651CP | Probable oxidoreductase |
| 96075-CP009426_prot_01181 | COG1024I | Probable enoyl-CoA hydratase EchA8 (enoyl hydrase) (unsaturated acyl-CoA hydratase) (crotonase) |
| 96075-CP009426_prot_02603 | COG3511M | Membrane-associated phospholipase C 2 PlcB |
| 96121-CP009427_prot_01212 | COG4907S | PE-PGRS family protein PE_PGRS21 |
| 96121-CP009427_prot_01300 | COG5373S | Conserved ala-, pro-rich protein |
| 96121-CP009427_prot_01474 | COG3464L | Probable transposase |
| 96121-CP009427_prot_00330 | COG5651N | PPE family protein PPE4 |
| 96121-CP009427_prot_01850 | COG3321Q | Probable polyketide synthase Pks7 |
| 96121-CP009427_prot_02855 | COG1982E | Probable amino acid decarboxylase |
| 96121-CP009427_prot_01706 | COG1819GC | Probable glycosyltransferase |
| 96121-CP009427_prot_00991 | COG3903R | Possible transcriptional regulatory protein (possibly LuxR-family) |
| 96121-CP009427_prot_03126 | COG0455D | Conserved hypothetical alanine rich protein |
| 96121-CP009427_prot_03541 | COG1231E | Probable flavin-containing monoamine oxidase AofH (amine oxidase) (MAO) |
| 96121-CP009427_prot_02735 | COG0714R | Conserved hypothetical protein |
| 96121-CP009427_prot_00390 | COG1113E | Possible L-asparagine permease AnsP2 (L-asparagine transport protein) |
| 96121-CP009427_prot_02945 | COG4762S | Conserved protein |
| 96121-CP009427_prot_03540 | COG1231E | Probable flavin-containing monoamine oxidase AofH (amine oxidase) (MAO) |
| 96121-CP009427_prot_04233 | COG3464L | Probable transposase |
| 96121-CP009427_prot_01738 | COG2409R | Probable conserved transmembrane transport protein MmpL4 |
| 96121-CP009427_prot_03131 | COG1321K | Probable transcriptional repressor SirR |
| 96121-CP009427_prot_01703 | COG1819GC | Probable glycosyltransferase |
| 96121-CP009427_prot_00037 | COG0156H | Possible 8-amino-7-oxononanoate synthase BioF2 (AONS) (8-amino-7-ketopelargonate synthase) (7-keto-8-amino-pelargonic acid syn> |
| 96121-CP009427_prot_01420 | COG0842T | ABC transporter, ATP-binding component [Rhodococcus jostii RHA1] |
| 323_CP010873.1_v2_prot_02343 | COG5665K | Conserved hypothetical protein |
| 323_CP010873.1_v2_prot_01849 | COG3321Q | Probable polyketide synthase Pks7 |
| 323_CP010873.1_v2_prot_02534 | COG0443O | Conserved hypothetical proline rich protein |
| 323_CP010873.1_v2_prot_03826 | COG0516F | Probable inosine-5'-monophosphate dehydrogenase GuaB3 (imp dehydrogenase) (inosinic acid dehydrogenase) (inosinate dehydrogena> |
| 323_CP010873.1_v2_prot_02716 | COG4552R | Enhanced intracellular survival protein Eis,GCN5-related N-acetyltransferase |
| 323_CP010873.1_v2_prot_02207 | COG0501O | Conserved protein |
| 323_CP010873.1_v2_prot_02342 | COG5665K | Conserved hypothetical protein |
| 323_CP010873.1_v2_prot_03624 | COG1752R | Probable conserved transmembrane transport protein |
| 323_CP010873.1_v2_prot_01964 | COG5164K | PE-PGRS family protein PE_PGRS31 |
| 323_CP010873.1_v2_prot_03190 | COG0174E | Probable glutamine synthetase GlnA4 (glutamine synthase) (GS-II) |
| 323_CP010873.1_v2_prot_00745 | COG1024I | Probable enoyl-CoA hydratase EchA5 (enoyl hydrase) (unsaturated acyl-CoA hydratase) (crotonase) |
| 323_CP010873.1_v2_prot_03745 | COG5651N | PPE family protein PPE54 |
| 323_CP010873.1_v2_prot_01966 | COG2234R | Conserved protein |
| 323_CP010873.1_v2_prot_03588 | COG1309K | Probable transcriptional regulatory protein (probably TetR-family) |
| NITR203-NC_021054_prot_04226 | COG1752R | Probable conserved two-domain membrane protein |
| NITR203-NC_021054_prot_00610 | COG0463M | Probable dolichyl-phosphate sugar synthase (dolichol-phosphate sugar synthetase) (dolichol-phosphate sugar transferase) (sugar> |
| NITR203-NC_021054_prot_03676 | COG0653U | Probable preprotein translocase SecA1 1 subunit |
| NITR203-NC_021054_prot_01997 | COG0393S | Unknown protein |
| NITR203-NC_021054_prot_04439 | COG0706U | Probable conserved transmembrane protein |
| NITR203-NC_021054_prot_02997 | COG0586S | Possible transmembrane protein DedA |
| NITR203-NC_021054_prot_02998 | COG0586S | Possible transmembrane protein DedA |
| NITR203-NC_021054_prot_02264 | COG0531E | Probable conserved integral membrane protein |
| NITR203-NC_021054_prot_03501 | COG1960I | Conserved hypothetical protein |
| NITR203-NC_021054_prot_00735 | COG0383G | Alpha-mannosidase |
| NITR203-NC_021054_prot_04036 | COG2852S | Conserved protein |
| NITR203-NC_021054_prot_03186 | COG3267U | Conserved hypothetical protein |
| NITR203-NC_021054_prot_01693 | COG1585OU | Conserved membrane protein |
| NITR203-NC_021054_prot_02446 | COG0769M | Probable UDP-N-acetylmuramoylalanyl-D-glutamate-2, 6-diaminopimelate ligase MurE |
| NITR203-NC_021054_prot_03040 | COG1232H | Probable protoporphyrinogen oxidase HemY (protoporphyrinogen-IX oxidase) (protoporphyrinogenase) (PPO) |
| NITR203-NC_021054_prot_00381 | COG0247C | Probable iron-sulfur-binding reductase |
| NITR203-NC_021054_prot_00827 | COG5662K | Anti-sigma factor RslA |
| NITR203-NC_021054_prot_03039 | COG1232H | Probable protoporphyrinogen oxidase HemY (protoporphyrinogen-IX oxidase) (protoporphyrinogenase) (PPO) |
| NITR203-NC_021054_prot_00302 | COG0146EQ | Probable 5-oxoprolinase OplA (5-oxo-L-prolinase) (pyroglutamase) (5-OPASE) |
| NITR203-NC_021054_prot_01443 | COG0515RTKL | Probable transmembrane serine/threonine-protein kinase H PknH (protein kinase H) (STPK H) |
| NITR203-NC_021054_prot_01745 | COG1063ER | Probable alcohol dehydrogenase Adh |
| NITR203-NC_021054_prot_01538 | COG1132V | Iron-regulated transporter IrtA |
| NITR203-NC_021054_prot_03192 | COG1343L | Conserved hypothetical protein |
| NITR203-NC_021054_prot_02370 | COG0006E | Dipeptidase PepE |
| NITR203-NC_021054_prot_00982 | COG2896H | Probable molybdenum cofactor biosynthesis protein A2 MoaA2 |
| NITR203-NC_021054_prot_04252 | COG0590FJ | Possible cytidine/deoxycytidylate deaminase |
| BT1-CP002883_prot_04306 | COG1674D | ESX conserved component EccC2. ESX-2 type VII secretion system protein. Possible membrane protein. |
| BT1-CP002883_prot_02515 | COG1293K | Conserved hypothetical protein |
| BT1-CP002883_prot_00880 | COG3547L | Putative transposase for insertion sequence element IS1547 |
| BT1-CP002883_prot_03291 | COG1181M | Probable D-alanine--D-alanine ligase DdlA (D-alanylalanine synthetase) (D-ala-D-ala ligase) |
| BT1-CP002883_prot_04002 | COG4842S | ESAT-6 like protein EsxJ (ESAT-6 like protein 2) |
| BT2-CP002882_prot_02206 | COG3945S | Unknown protein |
| BT2-CP002882_prot_04090 | COG0554C | Probable glycerol kinase GlpK (ATP:glycerol 3-phosphotransferase) (glycerokinase) (GK) |
| BT2-CP002882_prot_03071 | COG0686E | Secreted L-alanine dehydrogenase Ald (40 kDa antigen) (TB43) |
| BT2-CP002882_prot_01432 | COG2303E | Probable dehydrogenase FAD flavoprotein GMC oxidoreductase |
| BT2-CP002882_prot_02267 | COG1335Q | Pyrazinamidase/nicotinamidase PncA (PZase) |
| CCDC5079-NC_021251_prot_04243 | COG3321Q | Polyketide synthase Pks2 |
| CCDC5079-NC_021251_prot_00404 | COG3552R | Conserved hypothetical protein |
| CCDC5079-NC_021251_prot_03259 | COG3321Q | Probable polyketide synthase Pks15 |
| CCDC5079-NC_021251_prot_04153 | COG2217P | Probable cation transporter P-type ATPase CtpJ |
| CCDC5180-CP002885_prot_01979 | COG5651N | PPE family protein PPE28 |
| CDC1551-NC_002755_prot_02984 | COG1131V | Antibiotic-transport ATP-binding protein ABC transporter |
| CDC1551-NC_002755_prot_00655 | COG0392S | Probable conserved integral membrane protein |
| CDC1551-NC_002755_prot_03848 | COG4653R | Probable PhiRv1 phage protein |
| CDC1551-NC_002755_prot_01330 | COG1061KL | Unknown protein |
| CDC1551-NC_002755_prot_00299 | COG0146EQ | Probable 5-oxoprolinase OplA (5-oxo-L-prolinase) (pyroglutamase) (5-OPASE) |
| CDC1551-NC_002755_prot_01596 | COG0657I | Probable esterase LipO |
| CDC1551-NC_002755_prot_00548 | COG2303E | Probable oxidoreductase GMC-type |
| CDC1551-NC_002755_prot_01699 | COG2244R | Conserved probable membrane protein |
| CDC1551-NC_002755_prot_01700 | COG2244R | Conserved probable membrane protein |
| CDC1551-NC_002755_prot_03139 | COG1653G | Probable Sn-glycerol-3-phosphate-binding lipoprotein UgpB |
| CDC1551-NC_002755_prot_00103 | COG3261C | Possible formate hydrogenase HycE (FHL) |
| CDC1551-NC_002755_prot_03847 | COG4653R | Probable PhiRv1 phage protein |
| CDC1551-NC_002755_prot_00605 | COG5283S | Probable conserved integral membrane protein |
| CDC1551-NC_002755_prot_00944 | COG0031E | Possible cysteine synthase a CysK2 (O-acetylserine sulfhydrylase) (O-acetylserine (thiol)-lyase) (CSASE) |
| CDC1551-NC_002755_prot_01720 | COG3321Q | Probable polyketide synthase Pks5 |
| CDC1551-NC_002755_prot_00946 | COG2211G | Probable conserved integral membrane transport protein |
| CDC1551-NC_002755_prot_02622 | COG4842S | ESAT-6 like protein EsxJ (ESAT-6 like protein 2) |
| CDC1551-NC_002755_prot_03675 | COG1816F | Probable adenosine deaminase Add (adenosine aminohydrolase) |
| CDC1551-NC_002755_prot_01975 | COG5164K | PE-PGRS family protein PE_PGRS31 |
| CDC1551-NC_002755_prot_03953 | COG0436E | Possible aspartate aminotransferase AspB (transaminase A) (ASPAT) (glutamic--oxaloacetic transaminase) (glutamic--aspartic tra> |
| CTRI-2-NC_017524_prot_01336 | COG0318IQ | Probable fatty-acid-CoA ligase FadD36 (fatty-acid-CoA synthetase) (fatty-acid-CoA synthase) |
| CTRI-2-NC_017524_prot_02049 | COG0830O | Urease accessory protein UreF |
| CTRI-2-NC_017524_prot_03430 | COG2141C | Hypothetical oxidoreductase |
| CTRI-2-NC_017524_prot_01834 | COG3321Q | Probable polyketide synthase Pks7 |
| CTRI-2-NC_017524_prot_03665 | COG3119P | Probable arylsulfatase AtsB (aryl-sulfate sulphohydrolase) (sulfatase) |
| CTRI-2-NC_017524_prot_01121 | COG1947I | Probable 4-diphosphocytidyl-2-C-methyl-D-erythritol kinase IspE (CMK) (4-(cytidine-5'-diphospho)-2-C-methyl-D-erythritol kinas> |
| CTRI-2-NC_017524_prot_02509 | COG0443O | Conserved hypothetical proline rich protein |
| CTRI-2-NC_017524_prot_01337 | COG0318IQ | Probable fatty-acid-CoA ligase FadD36 (fatty-acid-CoA synthetase) (fatty-acid-CoA synthase) |
| EAI5-NC_021740_prot_03278 | COG1819GC | Possible glycosyl transferase |
| EAI5-NC_021740_prot_03277 | COG1819GC | Possible glycosyl transferase |
| EAI5-NC_021740_prot_01698 | COG3321Q | Probable polyketide synthase Pks5 |
| EAI5-NITR206-NC_021194_prot_03512 | COG2896H | Probable molybdenum cofactor biosynthesis protein A MoaA1 |
| EAI5-NITR206-NC_021194_prot_00312 | COG3285L | Conserved hypothetical protein |
| EAI5-NITR206-NC_021194_prot_04270 | COG1960I | Probable acyl-CoA dehydrogenase FadE35 |
| EAI5-NITR206-NC_021194_prot_00307 | COG2223P | Probable integral membrane nitrite extrusion protein NarU (nitrite facilitator) |
| EAI5-NITR206-NC_021194_prot_01686 | COG1240H | Probable membrane protein |
| EAI5-NITR206-NC_021194_prot_02592 | COG0775F | Conserved hypothetical protein |
| EAI5-NITR206-NC_021194_prot_04004 | COG2070R | Possible oxidoreductase |
| EAI5-NITR206-NC_021194_prot_01109 | COG1960I | Acyl-CoA dehydrogenase FadE12 |
| EAI5-NITR206-NC_021194_prot_01407 | COG2951M | Possible membrane protein |
| EAI5-NITR206-NC_021194_prot_01172 | COG2205T | Probable sensor protein KdpD |
| EAI5-NITR206-NC_021194_prot_03567 | COG1008C | Probable NADH dehydrogenase I (chain M) NUOK (NADH-ubiquinone oxidoreductase chain M) |
| EAI5-NITR206-NC_021194_prot_04198 | COG1752R | Probable conserved two-domain membrane protein |
| EAI5-NITR206-NC_021194_prot_03294 | COG1228Q | Conserved protein |
| EAI5-NITR206-NC_021194_prot_02681 | COG0319R | Conserved hypothetical protein |
| EAI5-NITR206-NC_021194_prot_04124 | COG1123R | Probable dipeptide-transport ATP-binding protein ABC transporter DppD |
| EAI5-NITR206-NC_021194_prot_03787 | COG5651N | PPE family protein PPE56 |
| EAI5-NITR206-NC_021194_prot_03982 | COG0119E | Probable 4-hydroxy-2-oxovalerate aldolase (HOA) |
| EAI5-NITR206-NC_021194_prot_00239 | COG2409R | Probable conserved transmembrane transport protein MmpL11 |
| EAI5-NITR206-NC_021194_prot_03277 | COG1526C | Possible FdhD protein homolog |
| EAI5-NITR206-NC_021194_prot_01860 | COG1190J | Lysyl-tRNA synthetase 2 LysX |
| EAI5-NITR206-NC_021194_prot_00438 | COG0543HC | Probable monooxygenase |
| EAI5-NITR206-NC_021194_prot_00081 | COG0112E | Serine hydroxymethyltransferase GlyA2 (serine methylase 2) (SHMT 2) |
| EAI5-NITR206-NC_021194_prot_01646 | COG0364G | Probable glucose-6-phosphate 1-dehydrogenase Zwf2 (G6PD) |
| EAI5-NITR206-NC_021194_prot_00082 | COG0112E | Serine hydroxymethyltransferase GlyA2 (serine methylase 2) (SHMT 2) |
| EAI5-NITR206-NC_021194_prot_04197 | COG1752R | Probable conserved two-domain membrane protein |
| EAI5-NITR206-NC_021194_prot_02376 | COG4581L | ATP-dependent DNA helicase HelY |
| EAI5-NITR206-NC_021194_prot_02479 | COG1022I | Long-chain-fatty-acid-CoA ligase FadD15 (fatty-acid-CoA synthetase) (fatty-acid-CoA synthase) |
| EAI5-NITR206-NC_021194_prot_03586 | COG0300R | Probable short-chain dehydrogenase/reductase |
| EAI5-NITR206-NC_021194_prot_01792 | COG4653R | Probable PhiRv1 phage protein |
| EAI5-NITR206-NC_021194_prot_01038 | COG0183I | Possible lipid carrier protein or keto acyl-CoA thiolase |
| EAI5-NITR206-NC_021194_prot_01067 | COG3285L | ATP dependent DNA ligase LigD (ATP dependent polydeoxyribonucleotide synthase) (thermostable DNA ligase) (ATP dependent polynu> |
| EAI5-NITR206-NC_021194_prot_02655 | COG0358L | Probable DNA primase DnaG |
| EAI5-NITR206-NC_021194_prot_03432 | COG2226H | Conserved protein |
| EAI5-NITR206-NC_021194_prot_02964 | COG1560M | Probable acyltransferase |
| EAI5-NITR206-NC_021194_prot_01111 | COG4770I | Probable acetyl-/propionyl-coenzyme A carboxylase alpha chain (alpha subunit) AccA2: biotin carboxylase + biotin carboxyl carr> |
| EAI5-NITR206-NC_021194_prot_03158 | COG2452L | Possible resolvase |
| EAI5-NITR206-NC_021194_prot_03983 | COG0119E | Probable 4-hydroxy-2-oxovalerate aldolase (HOA) |
| EAI5-NITR206-NC_021194_prot_02844 | COG1788I | Probable succinyl-CoA:3-ketoacid-coenzyme A transferase (alpha subunit) ScoA (3-oxo acid:CoA transferase) (OXCT A) (succinyl-C> |
| EAI5-NITR206-NC_021194_prot_04416 | COG0706U | Probable conserved transmembrane protein |
| EAI5-NITR206-NC_021194_prot_03157 | COG2452L | Possible resolvase |
| EAI5-NITR206-NC_021194_prot_01956 | COG0277C | Probable oxidoreductase |
| EAI5-NITR206-NC_021194_prot_04244 | COG0079E | Probable histidinol-phosphate aminotransferase HisC2 (imidazole acetol-phosphate transaminase) (imidazolylacetolphosphate amin> |
| EAI5-NITR206-NC_021194_prot_02721 | COG0555O | Probable sulfate-transport integral membrane protein ABC transporter CysT |
| EAI5-NITR206-NC_021194_prot_02545 | COG4799I | Acetyl/propionyl-CoA carboxylase (beta subunit) AccD6 |
| EAI5-NITR206-NC_021194_prot_01594 | COG1198L | Putative primosomal protein N' PriA (replication factor Y) |
| EAI5-NITR206-NC_021194_prot_01039 | COG0183I | Possible lipid carrier protein or keto acyl-CoA thiolase |
| EAI5-NITR206-NC_021194_prot_01791 | COG4653R | Probable PhiRv1 phage protein |
| EAI5-NITR206-NC_021194_prot_01643 | COG3429G | Putative OXPP cycle protein OpcA |
| EAI5-NITR206-NC_021194_prot_02908 | COG2256L | Conserved hypothetical alanine leucine valine rich protein |
| EAI5-NITR206-NC_021194_prot_00905 | COG1362E | Probable aminopeptidase PepC |
| EAI5-NITR206-NC_021194_prot_01874 | COG1364E | Probable glutamate N-acetyltransferase ArgJ |
| EAI5-NITR206-NC_021194_prot_00020 | COG0515RTKL | Transmembrane serine/threonine-protein kinase A PknA (protein kinase A) (STPK A) |
| EAI5-NITR206-NC_021194_prot_01684 | COG1721R | Conserved protein |
| EAI5-NITR206-NC_021194_prot_03989 | COG2030I | Probable dehydrogenase. Possible 2-enoyl acyl-CoA hydratase. |
| EAI5-NITR206-NC_021194_prot_02006 | COG3616E | Conserved protein |
| EAI5-NITR206-NC_021194_prot_00240 | COG2409R | Probable conserved transmembrane transport protein MmpL11 |
| EAI5-NITR206-NC_021194_prot_02144 | COG3315Q | Conserved hypothetical protein |
| EAI5-NITR206-NC_021194_prot_00316 | COG1960I | Probable acyl-CoA dehydrogenase FadE6 |
| EAI5-NITR206-NC_021194_prot_01084 | COG0739M | Conserved hypothetical protein |
| EAI5-NITR206-NC_021194_prot_03920 | COG1752R | Possible transmembrane protein |
| EAI5-NITR206-NC_021194_prot_03739 | COG0005F | Probable purine nucleoside phosphorylase DeoD (inosine phosphorylase) (PNP) |
| EAI5-NITR206-NC_021194_prot_00401 | COG0443O | Probable chaperone protein DnaK (heat shock protein 70) (heat shock 70 kDa protein) (HSP70) |
| EAI5-NITR206-NC_021194_prot_02397 | COG0638O | Proteasome beta subunit PrcB; assembles with alpha subunit PrcA. |
| EAI5-NITR206-NC_021194_prot_01669 | COG1960I | Probable acyl-CoA dehydrogenase FadE15 |
| EAI5-NITR206-NC_021194_prot_00601 | COG1333O | Probable conserved transmembrane protein |
| Erdman-ATCC-35801-NC_020559_prot_00077 | COG1615S | Probable conserved transmembrane protein |
| Erdman-ATCC-35801-NC_020559_prot_01110 | COG4591M | Probable adhesion component transport transmembrane protein ABC transporter |
| Erdman-ATCC-35801-NC_020559_prot_03249 | COG3653Q | Possible D-amino acid aminohydrolase (D-amino acid hydrolase) |
| Erdman-ATCC-35801-NC_020559_prot_01100 | COG3391S | PE-PGRS family protein PE_PGRS18 |
| Erdman-ATCC-35801-NC_020559_prot_00376 | COG0436E | Probable aspartate aminotransferase AspC (transaminase A) (ASPAT) |
| Erdman-ATCC-35801-NC_020559_prot_03140 | COG1332L | Hypothetical protein |
| Erdman-ATCC-35801-NC_020559_prot_01348 | COG0596R | Conserved hypothetical protein |
| Erdman-ATCC-35801-NC_020559_prot_02830 | COG1715V | Probable restriction system protein Mrr |
| Erdman-ATCC-35801-NC_020559_prot_00906 | COG2897P | Probable thiosulfate sulfurtransferase CysA3 (rhodanese-like protein) (thiosulfate cyanide transsulfurase) (thiosulfate thiotr> |
| Erdman-ATCC-35801-NC_020559_prot_00889 | COG3547L | Putative transposase for insertion sequence element IS1547 |
| Erdman-ATCC-35801-NC_020559_prot_02446 | COG0791M | Conserved hypothetical protein |
| Erdman-ATCC-35801-NC_020559_prot_03706 | COG0314H | Probable MoaD-MoaE fusion protein MoaX |
| Erdman-ATCC-35801-NC_020559_prot_02026 | COG1252C | Probable dehydrogenase |
| Erdman-ATCC-35801-NC_020559_prot_03509 | COG1894C | Probable NADH dehydrogenase I (chain F) NuoF (NADH-ubiquinone oxidoreductase chain F) |
| F1_prot_01214 | COG4907S | PE-PGRS family protein PE_PGRS21 |
| F1_prot_03383 | COG1104E | Cysteine desulfurase IscS (NIFS protein homolog) (nitrogenase metalloclusters biosynthesis protein NIFS) |
| F1_prot_03384 | COG0204I | Conserved hypothetical protein |
| F1_prot_03379 | COG0482J | Probable tRNA (5-methylaminomethyl-2-thiouridylate)-methyltransferase TrmU |
| F1_prot_03385 | COG0204I | Conserved hypothetical protein |
| F1_prot_03395 | COG0476H | Probable molybdenum cofactor biosynthesis protein MoeB2 (MPT-synthase sulfurylase) (molybdopterin synthase sulphurylase) |
| F1_prot_00540 | COG5002T | Putative two component sensor histidine kinase SenX3 |
| F1_prot_03380 | COG1104E | Cysteine desulfurase IscS (NIFS protein homolog) (nitrogenase metalloclusters biosynthesis protein NIFS) |
| F11-NC_009565_prot_01496 | COG0031E | Cysteine synthase B CysM (CSASE B) (O-phosphoserine sulfhydrylase B) (O-phosphoserine (thiol)-lyase B) |
| F11-NC_009565_prot_02544 | COG2134I | Probable CDP-diacylglycerol pyrophosphatase Cdh (CDP-diacylglycerol diphosphatase) (CDP-diacylglycerol phosphatidylhydrolase) |
| F11-NC_009565_prot_01983 | COG2124Q | Probable cytochrome P450 144 Cyp144 |
| F11-NC_009565_prot_02545 | COG2134I | Probable CDP-diacylglycerol pyrophosphatase Cdh (CDP-diacylglycerol diphosphatase) (CDP-diacylglycerol phosphatidylhydrolase) |
| F28_prot_03894 | COG1463Q | Mce-family protein Mce4D |
| F28_prot_00647 | COG3537G | Possible conserved exported protein |
| F28_prot_01732 | COG1053C | Probable fumarate reductase [flavoprotein subunit |
| F28_prot_00517 | COG2230M | Mycolic acid synthase PcaA (cyclopropane synthase) |
| F28_prot_03872 | COG5651N | PPE family protein PPE19 |
| F28_prot_00516 | COG2230M | Mycolic acid synthase PcaA (cyclopropane synthase) |
| F28_prot_03871 | COG5651N | PPE family protein PPE19 |
| H37Ra-NC_009525_prot_03730 | COG5651N | PPE family protein PPE56 |
| H37Ra-NC_009525_prot_01337 | COG5651N | PPE family protein PPE18 |
| H37Ra-NC_009525_prot_00117 | COG1020Q | Probable peptide synthetase Nrp (peptide synthase) |
| Haarlem-NC_022350_prot_03630 | COG1201R | Probable ATP-dependent helicase Lhr (large helicase-related protein) |
| Haarlem-NC_022350_prot_01535 | COG0458EF | Probable carbamoyl-phosphate synthase large chain CarB (carbamoyl-phosphate synthetase ammonia chain) |
| Haarlem-NC_022350_prot_03414 | COG0668M | Possible conserved transmembrane protein |
| Haarlem-NC_022350_prot_01694 | COG2409R | Probable conserved transmembrane transport protein MmpL12 |
| Haarlem-NC_022350_prot_01531 | COG0044F | Probable dihydroorotase PyrC (DHOase) |
| Haarlem-NC_022350_prot_01186 | COG3846U | PE-PGRS family protein PE_PGRS20 |
| HKBS1-CP002871_prot_01410 | COG3629T | Probable transcriptional regulatory protein EmbR |
| HKBS1-CP002871_prot_01266 | COG0644C | Possible oxidoreductase |
| HKBS1-CP002871_prot_01267 | COG0644C | Possible oxidoreductase |
| HKBS1-CP002871_prot_01268 | COG0644C | Possible oxidoreductase |
| K-CP007803_prot_01073 | COG4770I | Probable acetyl-/propionyl-coenzyme A carboxylase alpha chain (alpha subunit) AccA2: biotin carboxylase + biotin carboxyl carr> |
| KIT87190-CP007809_prot_04223 | COG2220R | Unknown protein |
| KIT87190-CP007809_prot_00697 | COG4398S | Conserved hypothetical protein |
| KIT87190-CP007809_prot_04190 | COG0782K | Hypothetical protein |
| KIT87190-CP007809_prot_03339 | COG5651N | PPE family protein PPE46 |
| KIT87190-CP007809_prot_01188 | COG3391S | PE-PGRS family protein PE_PGRS17 |
| KIT87190-CP007809_prot_00698 | COG4398S | Conserved hypothetical protein |
| KIT87190-CP007809_prot_04084 | COG1721R | Possible conserved membrane protein |
| KIT87190-CP007809_prot_04006 | COG2968S | Probable conserved lipoprotein LpqG |
| Kurono-NZ_AP014573_prot_01589 | COG2508TQ | Conserved protein |
| Kurono-NZ_AP014573_prot_04274 | COG0455D | Conserved protein |
| Kurono-NZ_AP014573_prot_02028 | COG0789K | Conserved protein |
| KZN-605-NC_018078_prot_03026 | COG0624E | Probable succinyl-diaminopimelate desuccinylase DapE |
| KZN-605-NC_018078_prot_02488 | COG1386K | Possible segregation and condensation protein ScpB |
| KZN-605-NC_018078_prot_00066 | COG0305L | Probable replicative DNA helicase DnaB |
| KZN-605-NC_018078_prot_02489 | COG1386K | Possible segregation and condensation protein ScpB |
| KZN-605-NC_018078_prot_01595 | COG2030I | Possible oxidase regulatory-related protein |
| KZN-605-NC_018078_prot_03245 | COG1197LK | Probable transcription-repair coupling factor Mfd (TRCF) |
| KZN-605-NC_018078_prot_03107 | COG0620E | Probable 5-methyltetrahydropteroyltriglutamate--homocysteine methyltransferase MetE (methionine synthase, vitamin-B12 independ> |
| KZN-605-NC_018078_prot_01928 | COG0115EH | Branched-chain amino acid transaminase IlvE |
| KZN-1435-NC_012943_prot_03020 | COG0294H | Dihydropteroate synthase 2 FolP2 (DHPS 2) (dihydropteroate pyrophosphorylase 2) |
| KZN-1435-NC_012943_prot_01890 | COG0304IQ | 3-oxoacyl-[acyl-carrier protein |
| KZN-1435-NC_012943_prot_03232 | COG0642T | Two component sensor histidine kinase TrcS |
| KZN-1435-NC_012943_prot_03322 | COG0739M | Conserved hypothetical protein |
| KZN-1435-NC_012943_prot_03390 | COG2072P | Probable monooxygenase |
| KZN-1435-NC_012943_prot_03323 | COG0739M | Conserved hypothetical protein |
| KZN-1435-NC_012943_prot_03026 | COG3899R | Conserved hypothetical protein |
| KZN-1435-NC_012943_prot_03025 | COG3899R | Conserved hypothetical protein |
| KZN-1435-NC_012943_prot_00589 | COG1575H | 1,4-dihydroxy-2-naphthoate octaprenyltransferase MenA (DHNA-octaprenyltransferase) |
| KZN-1435-NC_012943_prot_03047 | COG2508TQ | Conserved protein |
| KZN-1435-NC_012943_prot_03426 | COG2378K | Conserved protein |
| KZN-1435-NC_012943_prot_02410 | COG1640G | Probable 4-alpha-glucanotransferase MalQ (amylomaltase) (disproportionating enzyme) (D-enzyme) |
| KZN-4207-NC_016768_prot_00267 | COG1309K | Probable transcriptional regulatory protein (probably TetR/AcrR-family) |
| ZMC13-88-CP009101_prot_02613 | COG4842S | ESAT-6 like protein EsxK (ESAT-6 like protein 3) |
| ZMC13-88-CP009101_prot_02153 | COG0543HC | Possible oxygenase |
| ZMC13-88-CP009101_prot_03468 | COG0314H | Probable molybdenum cofactor biosynthesis protein E MoaE1 (molybdopterin converting factor large subunit) (molybdopterin [MPT]> |
| ZMC13-88-CP009101_prot_01079 | COG4799I | Probable acetyl-/propionyl-CoA carboxylase (beta subunit) AccD2 |
| ZMC13-88-CP009101_prot_01338 | COG4842S | ESAT-6 like protein EsxK (ESAT-6 like protein 3) |
| ZMC13-88-CP009101_prot_01078 | COG4770I | Probable acetyl-/propionyl-coenzyme A carboxylase alpha chain (alpha subunit) AccA2: biotin carboxylase + biotin carboxyl carr> |
| ZMC13-88-CP009101_prot_03469 | COG0314H | Probable molybdenum cofactor biosynthesis protein E MoaE1 (molybdopterin converting factor large subunit) (molybdopterin [MPT]> |
| ZMC13-88-CP009101_prot_00431 | COG0626E | Probable O-succinylhomoserine sulfhydrylase MetZ (OSH sulfhydrylase) |
| ZMC13-88-CP009101_prot_01340 | COG3328L | Probable transposase |
| ZMC13-88-CP009101_prot_00209 | COG1741R | Conserved hypothetical protein |
| ZMC13-88-CP009101_prot_02152 | COG0543HC | Possible oxygenase |
| ZMC13-264-CP009100_prot_02264 | COG2312R | Conserved protein |
| ZMC13-264-CP009100_prot_00043 | COG0318IQ | Probable fatty-acid-CoA ligase FadD34 (fatty-acid-CoA synthetase) (fatty-acid-CoA synthase) |
| ZMC13-264-CP009100_prot_02576 | COG2128S | Hypothetical protein |
| ZMC13-264-CP009100_prot_00902 | COG0115EH | Probable amino acid aminotransferase |
| ZMC13-264-CP009100_prot_01488 | COG3191EQ | Probable hydrolase |
| ZMC13-264-CP009100_prot_03835 | COG5178A | Conserved hypothetical alanine and proline rich protein |
