# Supplementary material for: Pan-Genomic Study of Mycobacterium tuberculosis Reflecting the Primary/Secondary Genes, Generality/Individuality, and the Interconversion Through Copy Number Variations
Source: Front Microbiol. 2018 Aug 17;9:1886. doi: 10.3389/fmicb.2018.01886 (PMC6109687; doi:10.3389/fmicb.2018.01886)
Supplement: Supplementary file 14 [file Data_Sheet_1.PDF]

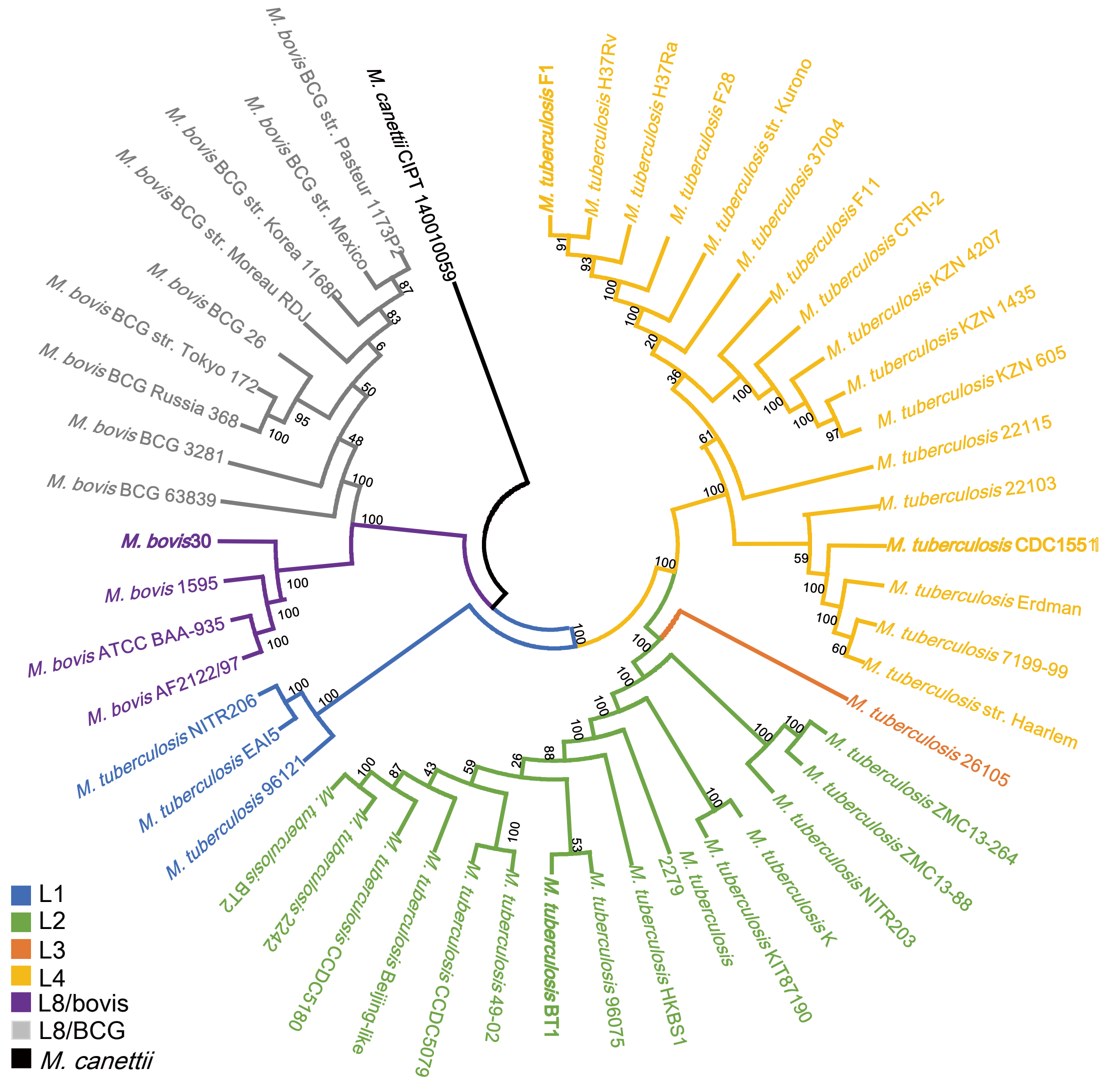

Supplementary Figure S1. Phylogenetic analysis of the 36 Mtb and 13 Mbo strains. The tree is rooted with *M. canettii* CIPT 140010059. Different lineage strains are marked with different colors: L1 strains, blue; L2 strains, green; L3 strains, salmon; L4 strains, gold; L8/bovis strains, purple; L8/BCG strains, grey. Numbers on the branches represent the bootstrap values.
