# Supplementary material for: Pan-Genomic Study of Mycobacterium tuberculosis Reflecting the Primary/Secondary Genes, Generality/Individuality, and the Interconversion Through Copy Number Variations
Source: Front Microbiol. 2018 Aug 17;9:1886. doi: 10.3389/fmicb.2018.01886 (PMC6109687; doi:10.3389/fmicb.2018.01886)
Supplement: Supplementary file 15 [file Data_Sheet_2.PDF]

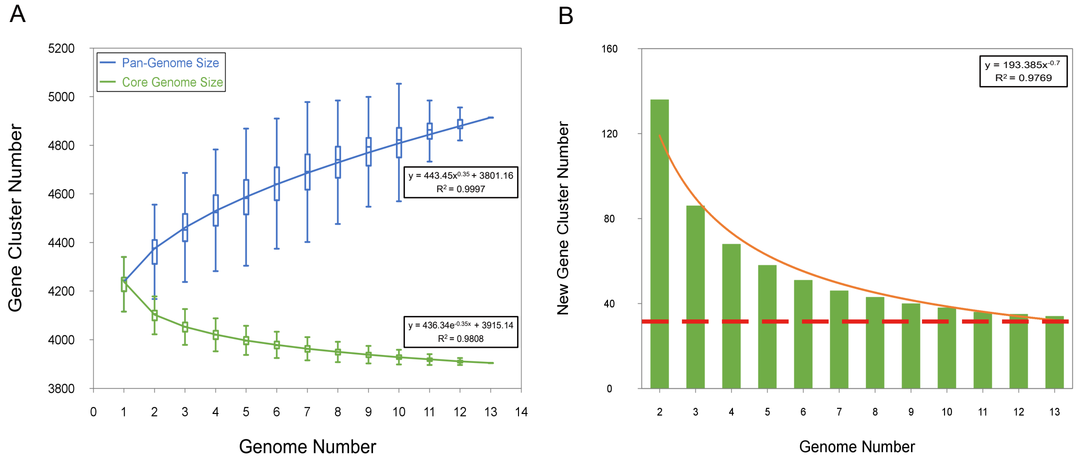

Supplementary Figure S2. The pan-genome of Mbo. (A) Gene accumulation curves of the pan-genome (blue) and the core-genome (green). The blue boxes denote the Mbo pan-genome size for each genomic comparison. The green boxes show the Mbo core genome size for each genomic comparison. The curve is the least squares fit of the power law for the average values. (B) Curve (red) showing the number of new genes against increase in the number of Mbo genomes.
