# Supplementary material for: Pan-Genomic Study of Mycobacterium tuberculosis Reflecting the Primary/Secondary Genes, Generality/Individuality, and the Interconversion Through Copy Number Variations
Source: Front Microbiol. 2018 Aug 17;9:1886. doi: 10.3389/fmicb.2018.01886 (PMC6109687; doi:10.3389/fmicb.2018.01886)
Supplement: Supplementary file 16 [file Data_Sheet_3.PDF]

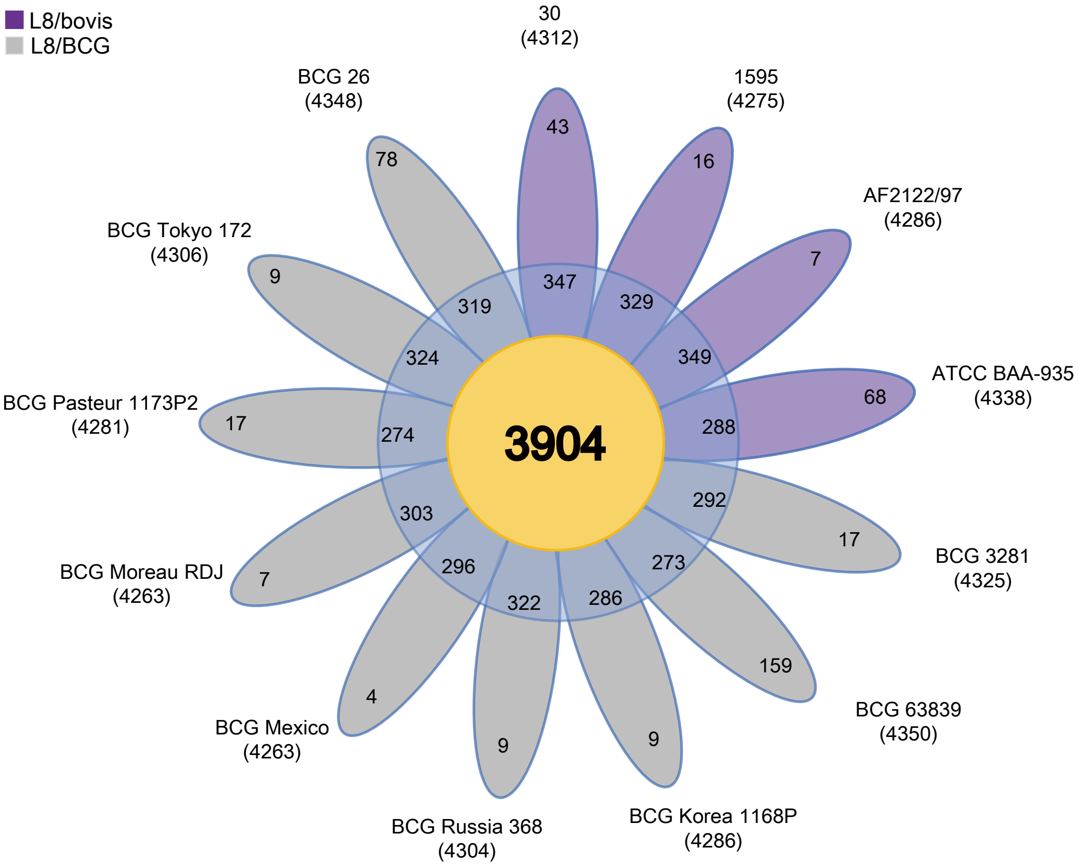

Supplementary Figure S3. Flower plot showing the core, dispensable, and strain-specific genes of the 13 Mtb strains. The flower plot shows the core gene number (in the center), dispensable gene number (in the annulus), and the strain-specific gene number (in the petals) for the 13 Mbo strains. The numbers under the strain name denote the total number of related genes. Purple indicates L8/bovis strains and grey represents L8/BCG strains.
