# Supplementary material for: Pan-Genomic Study of Mycobacterium tuberculosis Reflecting the Primary/Secondary Genes, Generality/Individuality, and the Interconversion Through Copy Number Variations
Source: Front Microbiol. 2018 Aug 17;9:1886. doi: 10.3389/fmicb.2018.01886 (PMC6109687; doi:10.3389/fmicb.2018.01886)
Supplement: Supplementary file 17 [file Data_Sheet_4.PDF]

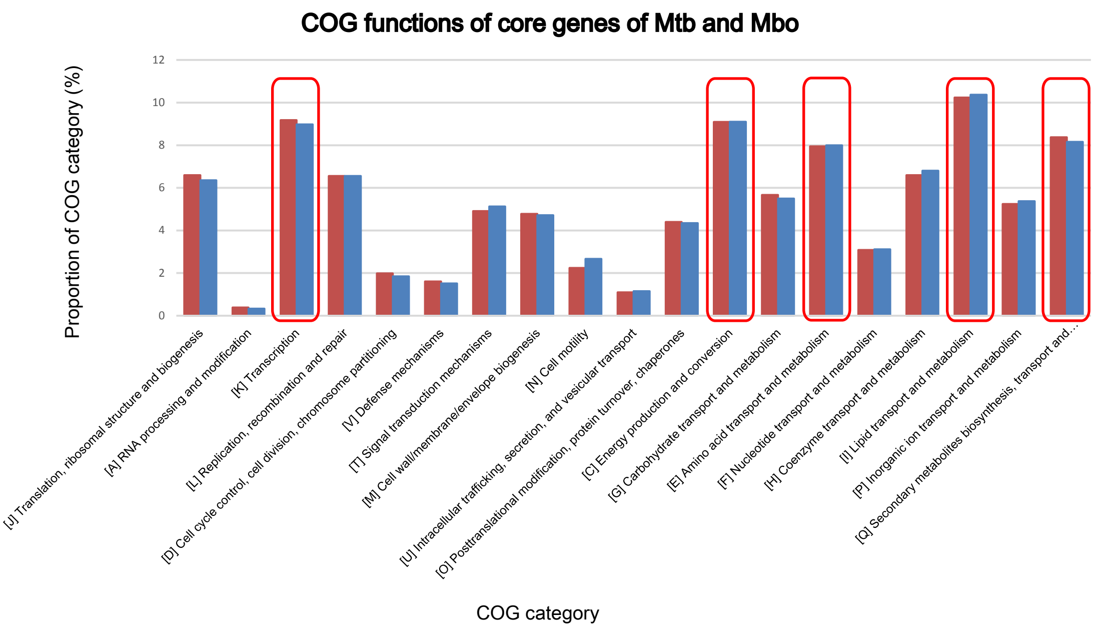

Supplementary Figure S4. Functional classes of Mtb (red) and Mbo core genes (blue) across COG categories. The COG functional categories are shown on the x-axis. The percentage of related genes for each COG functional category is shown on the y-axis.
