# Supplementary material for: Pan-Genomic Study of Mycobacterium tuberculosis Reflecting the Primary/Secondary Genes, Generality/Individuality, and the Interconversion Through Copy Number Variations
Source: Front Microbiol. 2018 Aug 17;9:1886. doi: 10.3389/fmicb.2018.01886 (PMC6109687; doi:10.3389/fmicb.2018.01886)
Supplement: Supplementary file 18 [file Data_Sheet_5.PDF]

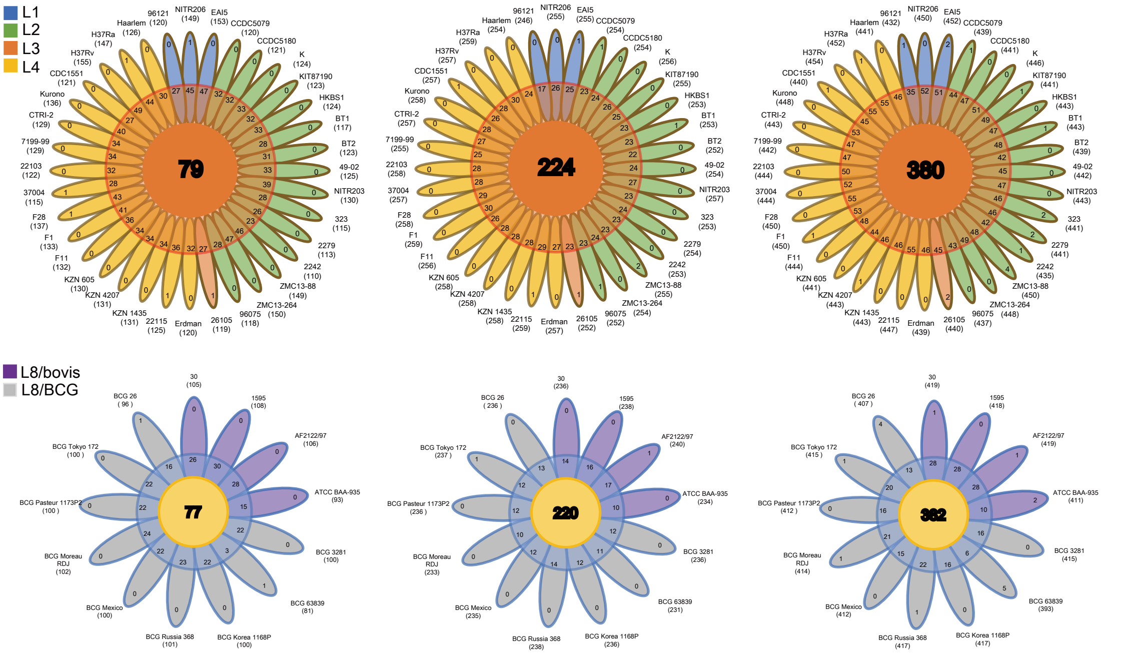

Supplementary Figure S5. Flower plot showing the core, dispensable, and strain-specific PE/PPE, VF, and antigen genes in Mtb and Mbo strains. (A) Flower plots showing the core PE/PPE protein number (in the center), dispensable PE/PPE protein number (in the annulus), and strain-specific PE/PPE protein number (in the petals) for the 36 Mtb strains. (B) Flower plots showing the core VF number (in the center), dispensable VF number (in the annulus), and strain-specific VF number (in the petals) for the 36 Mtb strains. (C) Flower plots showing the core antigen number (in the center), dispensable antigen number (in the annulus), and strain-specific antigen number (in the petals) for the 36 Mtb strains. (D) Flower plots showing the core PE/PPE protein number (in the center), dispensable PE/PPE protein number (in the annulus), and strain-specific PE/PPE protein number (in the petals) for the 13 Mbo strains. (E) Flower plots showing the core VF number (in the center), dispensable VF number (in the annulus), and strain-specific VF number (in the petals) for the 13 Mbo strains. (F) Flower plots showing the core antigen number (in the center), dispensable antigen number (in the annulus), and strain-specific antigen number (in the petals) for the 13 Mbo strains. The numbers under the strain names represent the total number of related genes. Different colors indicate different lineages: L1 strains, blue; L2 strains, green; L3 strains, salmon; L4 strains, gold; L8/bovis strains, purple; L8/bovis strains, grey.
