# Supplementary material for: Pan-Genomic Study of Mycobacterium tuberculosis Reflecting the Primary/Secondary Genes, Generality/Individuality, and the Interconversion Through Copy Number Variations
Source: Front Microbiol. 2018 Aug 17;9:1886. doi: 10.3389/fmicb.2018.01886 (PMC6109687; doi:10.3389/fmicb.2018.01886)
Supplement: Supplementary file 19 [file Data_Sheet_6.PDF]

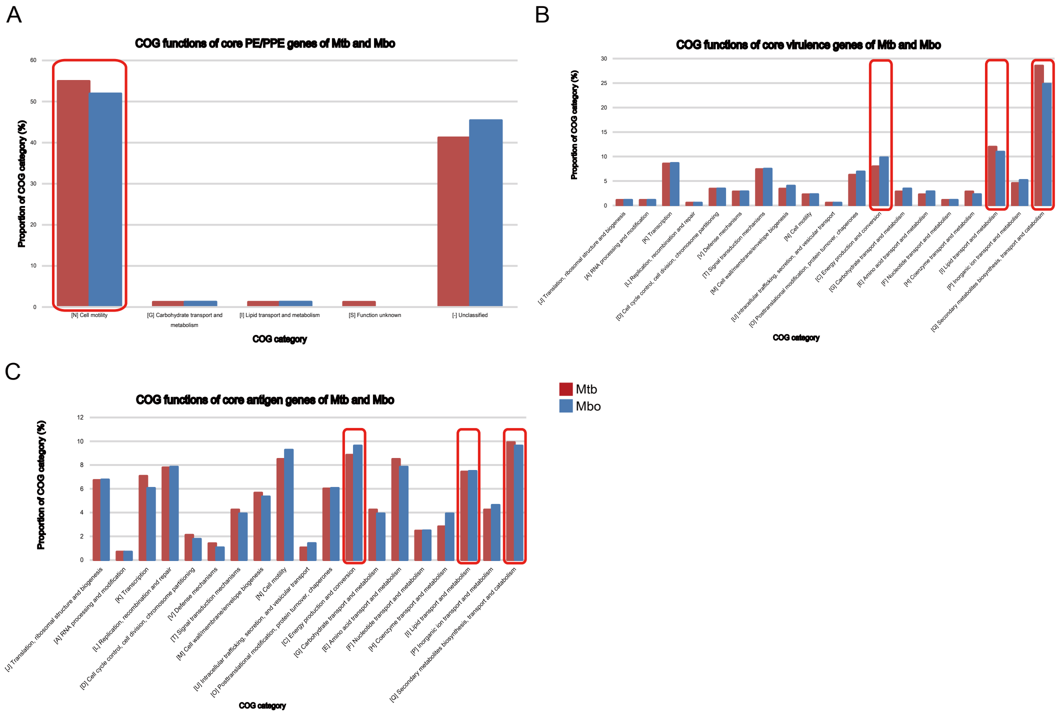

Supplementary Figure S6. Functional classes of the core PE/PPE, virulence, and antigen genes for Mtb/Mbo strains. (A) Functional classes of Mtb and Mbo core PE/PPE genes across COG categories. (B) Functional classes of Mtb and Mbo core virulence genes across COG categories. (C) Functional classes of Mtb and Mbo core antigen genes across COG categories. Red indicates Mtb genes and blue represents Mbo genes. The COG functional categories are shown on the x-axis. The percentage of related genes in the COG functional category are shown on the y-axis.
