# Supplementary material for: Pan-Genomic Study of Mycobacterium tuberculosis Reflecting the Primary/Secondary Genes, Generality/Individuality, and the Interconversion Through Copy Number Variations
Source: Front Microbiol. 2018 Aug 17;9:1886. doi: 10.3389/fmicb.2018.01886 (PMC6109687; doi:10.3389/fmicb.2018.01886)
Supplement: Supplementary file 22 [file Data_Sheet_9.PDF]

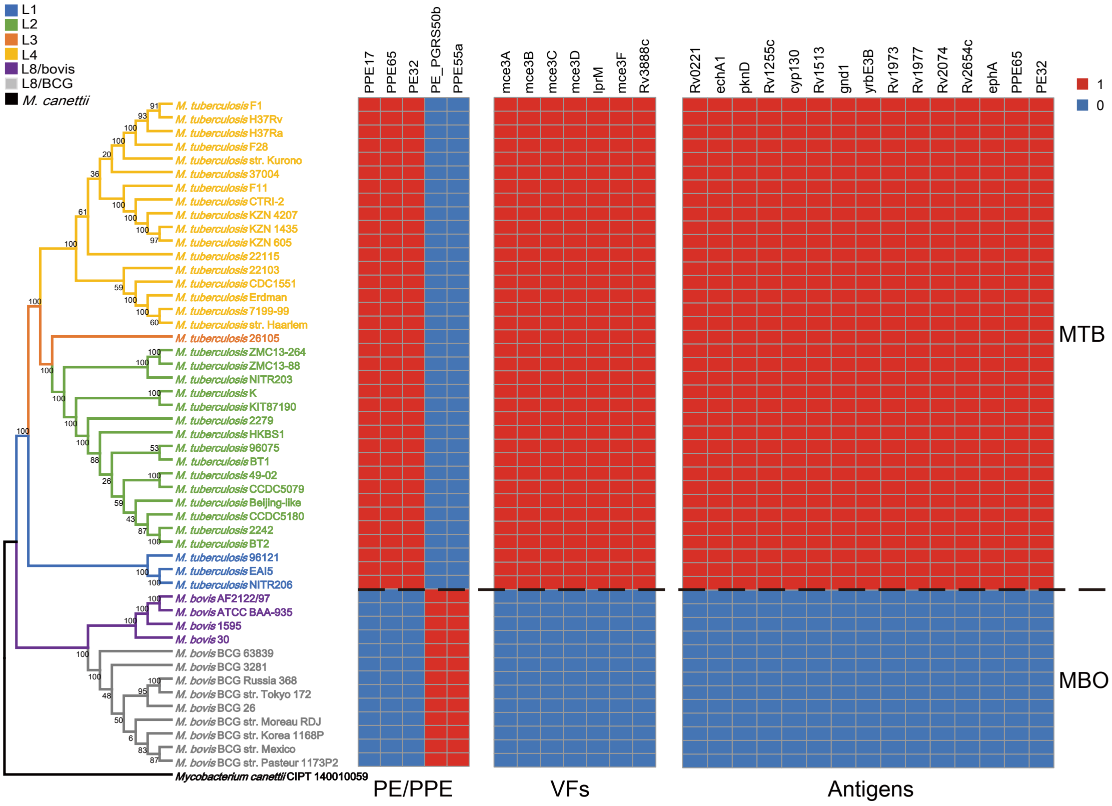

Supplementary Figure S9. Mtb and Mbo specific single-copy core PE/PPE, virulence, and antigen genes. The schematic diagram shows the 23 Mtb and two Mbo specific single-copy core PE/PPE, virulence, and antigen genes. Rows represent the 36 Mtb and 13 Mbo strains; columns represent the Mtb and Mbo specific single-copy core PE/PPE, virulence, and antigen genes.
