# Supplementary material for: Pan-Genomic Study of Mycobacterium tuberculosis Reflecting the Primary/Secondary Genes, Generality/Individuality, and the Interconversion Through Copy Number Variations
Source: Front Microbiol. 2018 Aug 17;9:1886. doi: 10.3389/fmicb.2018.01886 (PMC6109687; doi:10.3389/fmicb.2018.01886)
Supplement: Supplementary file 25 [file Data_Sheet_12.PDF]

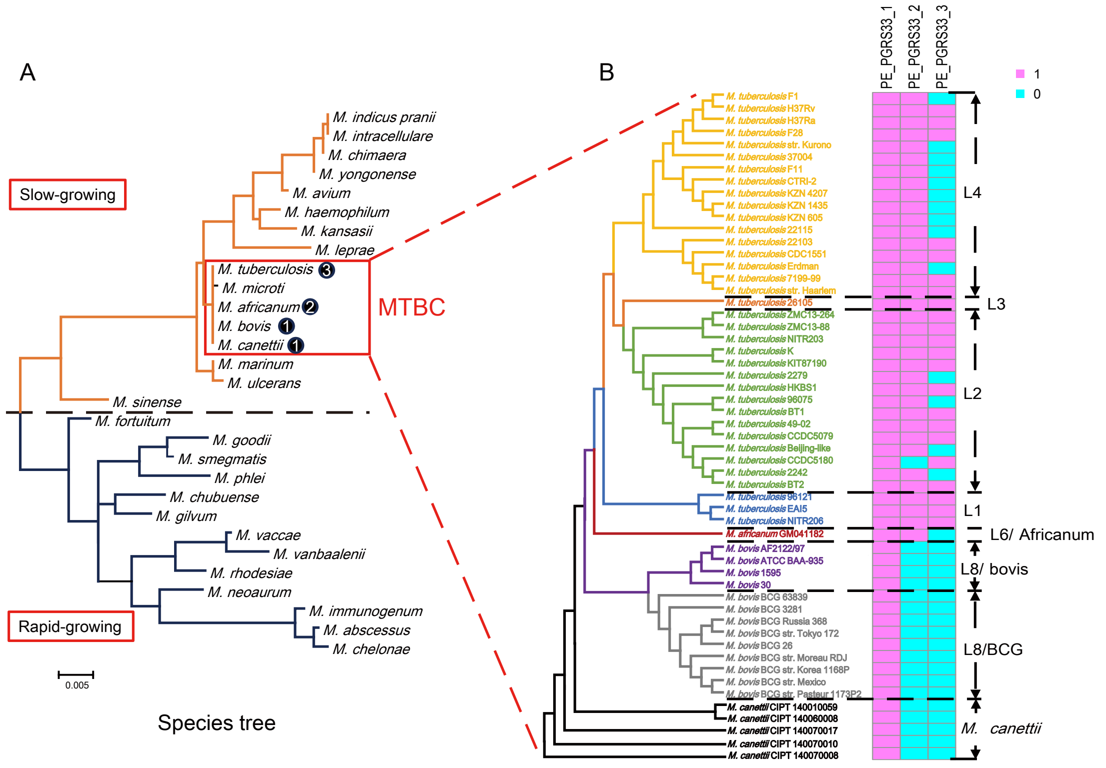


**Supplementary Figure S12.** The evolution of three copies of *PE_PGRS33*. (A) The tree was constructed on the basis of the 16S rRNA genes using the maximum likelihood method. The numbers in black circles indicate the copy number of *PE_PGRS33* for each species. (B) A schematic diagram showing the distribution of the three *PE_PGRS33* copies in MTBC strains. The rows show the 36 Mtb, one *M. africanum*, 13 Mbo, and five STB strains. The columns show the three *PE_PGRS33* copies.
