# Supplementary material for: Pan-Genomic Study of Mycobacterium tuberculosis Reflecting the Primary/Secondary Genes, Generality/Individuality, and the Interconversion Through Copy Number Variations
Source: Front Microbiol. 2018 Aug 17;9:1886. doi: 10.3389/fmicb.2018.01886 (PMC6109687; doi:10.3389/fmicb.2018.01886)
Supplement: Supplementary file 26 [file Data_Sheet_13.PDF]

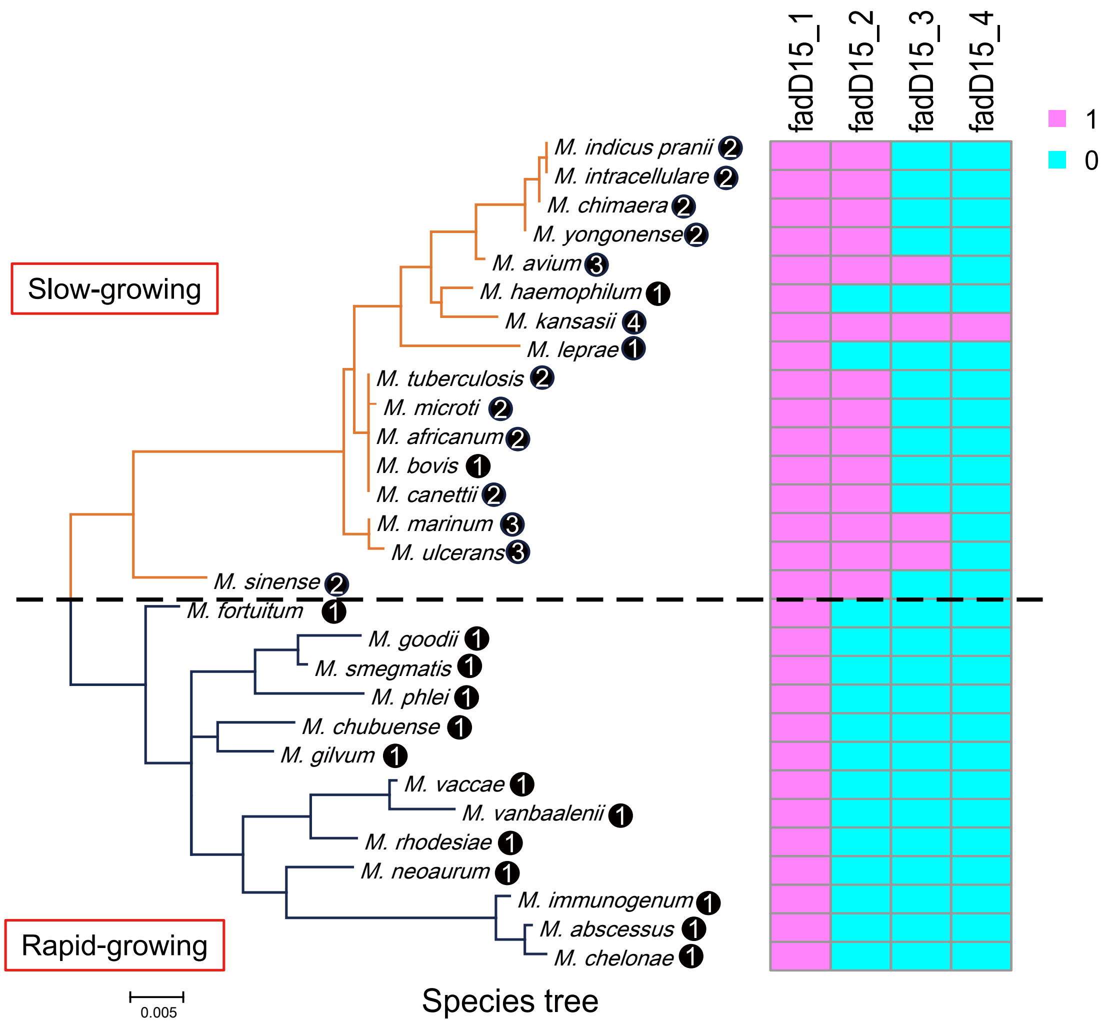

Supplementary Figure S13. An evolutionary tree of the four *fadD15* copies in mycobacteria species (including fast-/slow-growing mycobacteria). The schematic diagram shows the distribution of the four *fadD15* copies across mycobacteria strains. The rows show the 29 mycobacteria strains. The columns show the four *fadD15* copies. The numbers in black circles represent the copy number of *fadD15* for each species.
