# Supplementary material for: Pan-Genomic Study of Mycobacterium tuberculosis Reflecting the Primary/Secondary Genes, Generality/Individuality, and the Interconversion Through Copy Number Variations
Source: Front Microbiol. 2018 Aug 17;9:1886. doi: 10.3389/fmicb.2018.01886 (PMC6109687; doi:10.3389/fmicb.2018.01886)
Supplement: Supplementary file 27 [file Data_Sheet_14.PDF]

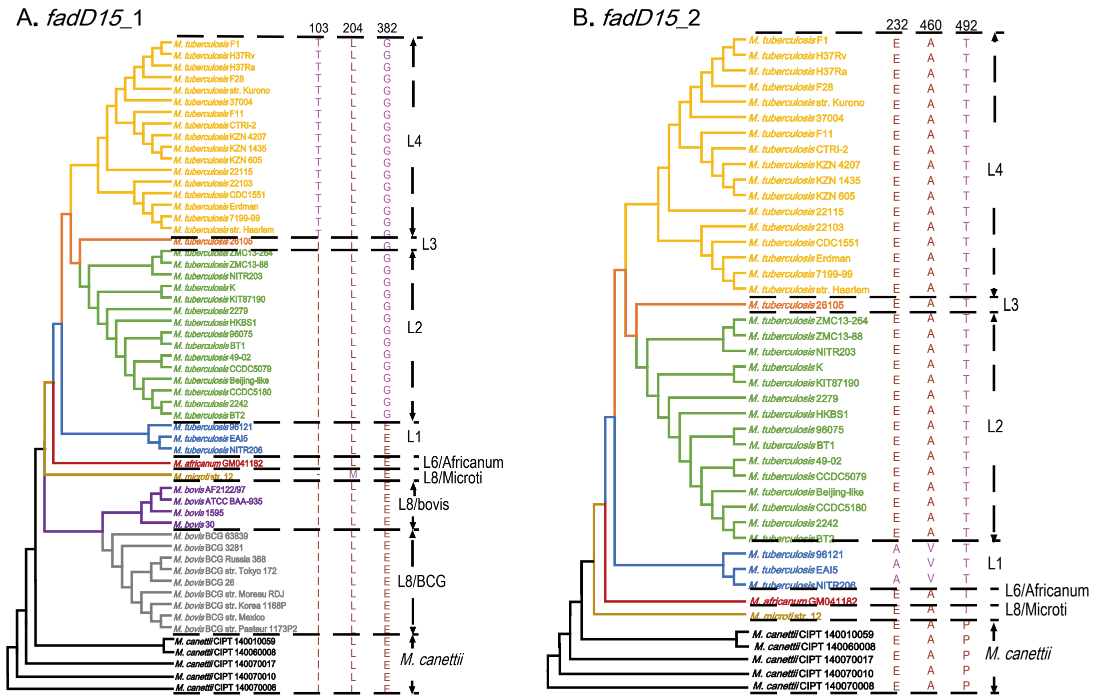

Supplementary Figure S14. The lineage-specific amino acid substitutions in *fadD15*_1 (A) and *fadD15*_2 (B) found in MTBC strains. Different colors indicate different MTBC lineage strains. The substituted amino acids are displayed in pink, and the corresponding STB controls are shown in brown.
