# Supplementary material for: Pan-Genomic Study of Mycobacterium tuberculosis Reflecting the Primary/Secondary Genes, Generality/Individuality, and the Interconversion Through Copy Number Variations
Source: Front Microbiol. 2018 Aug 17;9:1886. doi: 10.3389/fmicb.2018.01886 (PMC6109687; doi:10.3389/fmicb.2018.01886)
Supplement: Supplementary file 21 [file Data_Sheet_8.PDF]

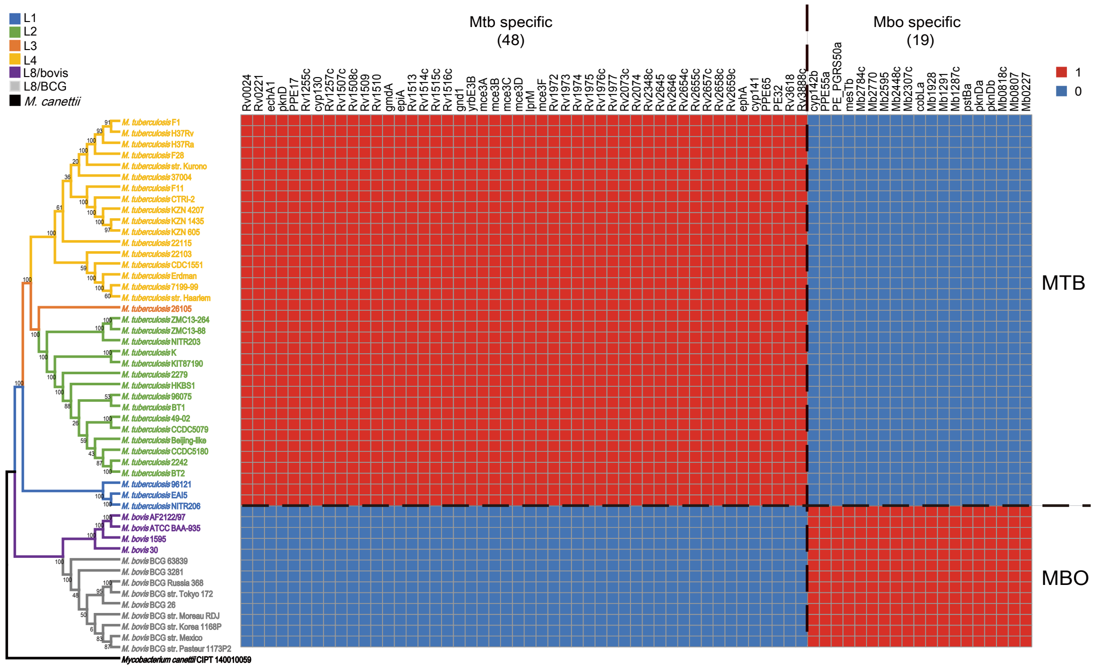

Supplementary Figure S8. Schematic diagram showing the single-copy core genes specific to Mtb/Mbo strains. Rows represent the 36 Mtb and 13 Mbo strains; columns represent the Mtb- and Mbo-specific genes.
